# Blockade of exosome generation by GW4869 inhibits the education of M2 macrophages in prostate cancer

**DOI:** 10.1186/s12865-022-00514-3

**Published:** 2022-08-08

**Authors:** Yilin Peng, Min Zhao, Yinying Hu, Hongyan Guo, Yanyan Zhang, Yanqin Huang, Lin Zhao, Yong Chai, Zhigang Wang

**Affiliations:** 1 Jiangxi Academy of Medical Sciences, and Jiangxi Provincial Key Laboratory of Immunotherapy, Nanchang, 330006 Jiangxi China; 2 Medical College of Nanchang University, Nanchang, 330006 Jiangxi China; 3 Department of Ophthalmology, the Affiliated Children’s Hospital of Nanchang University, Nanchang, 330006 Jiangxi China; 4 Department of Immunology and Rheumatology, the First Affiliated Hospital of Nanchang University, Nanchang, 330006 Jiangxi China

**Keywords:** Exosome, GW4869, Macrophage polarization, Prostate cancer

## Abstract

**Background:**

Tumor-associated macrophages are considered to be a major contributor affecting the development of tumors. Recently, numerous studies have shown that tumor cells were able to educate their microenvironment by delivering a significant amount of exosomes, however, the mechanism that exosomes from PCa cells work in macrophage polarization remains obscure. Therefore, we sought to determine whether blockade of exosome generation by GW4869, an inhibitor of exosome biogenesis, would impede macrophages from differentiating into M2 cells.

**Results:**

In this study, we first obtained exosomes from the supernatant media of PCa cells cultured with exosome-free serum using the Magcapture™ Exosome Isolation Kit PS, and then investigated their effects on macrophages. Our data confirmed that exosomes released by prostate cancer cells can induce macrophages to differentiate into M2 cells. Mechanistically speaking, exosomes exert their effects on macrophages through activating the AKT and STAT3 signaling pathways. Importantly, treatment with GW4869 significantly inhibited the release of exosomes from PCa cells, and further impaired M2 differentiation of macrophages and their pro-tumor activity. We also demonstrated that GW4869 was able to inhibit the education of M2 macrophages, and then inhibit the progression of prostate cancer in vivo.

**Conclusions:**

In brief, our findings indicated that GW4869 impeded the PCa exosome-induced M2 differentiation of macrophages and the progression of prostate cancer, suggesting that GW4869 could play an important role in the treatment of prostate cancer metastasis as an inhibitor of tumor exosome secretion.

**Supplementary Information:**

The online version contains supplementary material available at 10.1186/s12865-022-00514-3.

## Background

Currently, prostate cancer remains one of the main causes of male cancer-related death worldwide, and in particular, metastatic prostate cancer is challenging to manage due to the lack of effective therapeutic methods [1]. Hence, exploring the molecular mechanisms of prostate cancer is of great significance for its diagnosis and treatment.

Macrophages in a tumor microenvironment, tumor-associated macrophages (TAMs), are considered a major contributor to promoting the progression of multiple tumors. Multiple studies have supported the hypothesis that TAMs in tumor tissues and adjacent tissues, similar to M2 macrophages, show pro-tumor phenotypes in terms of promoting tumor cell proliferation, invasion, and metastasis [2, 3]. Moreover, several reports have confirmed that TAM infiltration is an independent risk factor for prostate cancer recurrence [4], and the prognosis of patients with decreased TAM numbers is significantly better than that of those with higher numbers, indicating that the number of TAMs is related to the prognosis of PCa [5].

Macrophages usually can be divided into M1 (classically activated macrophages) and M2 (alternatively activated macrophages) phenotypes [6]. The former secretes a variety of inflammatory factors and enzyme molecules, including iNOS and TNF-α, to kill infectious pathogens and tumor cells, while the latter secretes a variety of tumor cell growth promoting factors, vasoactive substances, and some matrix metalloproteinases (MMPs) to promote tumor growth, migration and invasion. Macrophage polarization is a dynamic process of phenotype transition. It has been accepted that many factors affect the M1/M2 transition of macrophages, such as key cytokines in the tumor microenvironment and the activation of signal transduction pathways in macrophages [7–9]. Recently, evidence has indicated that exosomes, a class of 30–150 nm extracellular vesicles, can act as a carrier for intercellular signal exchange and play an important role in educating tumor the micro-environment [10]. By fusing with the cell membrane of other cells, exosomes can easily selectively deliver bioactive substances such as proteins, lipids and RNAs to recipient cells, communicate information, and then affect the physiological process of recipient cells [11, 12]. Many studies have confirmed that tumor cells can regulate immune cells and cultivate a tumor microenvironment suitable for their growth and metastasis by secreting more exosomes than normal cells [13]. For example, the exosomes of gastric cancer cells promote liver metastasis in gastric cancer [14], and the exosomes of pancreatic cancer cells can induce macrophages to exhibit M2 phenotypes in vitro [15]. Recent research has demonstrated that prostate cancer exosomes can promote the proliferation and metastasis of prostate cancer [16], and induce macrophages to differentiate into the M2 phenotype, however, the molecular mechanisms through which PCa exosomes mediate macrophage differentiation have not been fully clarified.

In 2010, GW4869, a neutral sphingomyelinase inhibitor [17], was first utilized by Kosaka et al. [18] to successfully inhibit the release of mature exosomes from multivesicular bodies (MVBs) in HEK293 cells. Therefore, we sought to determine whether the blockade of exosome release in PCa cells would be able to affect macrophage polarization.

In this study, we confirmed that exosomes from prostate cancer cells could induce macrophages to differentiate into the M2 phenotype through the STAT3 signaling pathway. In particular, this induction could be inhibited by GW4869, which reversed this macrophage polarization.

## Materials and methods

### Experimental cell lines

PC-3M-2B4 (a human prostate cancer low metastatic cell line) cells and PC-3M-1E8 (a human prostate cancer high metastatic cell line) cells were obtained from the National Infrastructure of Cell Line Resource (NICR). THP-1 (a human acute mononuclear leukemia cell line) and HUVEC (a human umbilical vein endothelial cell line) cells were purchased from ATCC.

### THP-1 cell differentiation

THP-1 cells was cultured in RPMI1640 with 10% exosome-free FBS and 0.05 mmol/L β-mercaptoethanol and then induced to differentiate into macrophages with 10 ng/mL PMA for 72 h. After that, the macrophages were treated with IFN-γ (50 ng/mL) plus LPS (15 ng/mL) or IL-4 (25 ng/mL) and differentiated into M1 or M2 macrophages, respectively, and untreated cells were used as a control group (M0). Exosomes or cell supernatant from the PCa cells treated with GW4869 were also added to the macrophages to observe their effects on macrophage function.

### PCa cells treated with GW4869

PC-3M-2B4 and PC-3M-1E8 cells were cultured in RPMI1640 with 10% exosome-free FBS. When the growth density of PC-3M-2B4 and PC-3M-1E8 cells reached 70%, GW4869 was added to these cells at 10 μM. After 48 h, the cell supernatant was collected and centrifuged at 400 g for 10 min to remove precipitates, then the supernatant was filtered through a 0.22 μm pore size filter. Based on the specific cell source, these supernatants were named 2B4-GW4869 or 1E8-GW4869.

### Extraction of exosomes

After filtration, 80 mL of cell supernatant was filtered using an Amicon^®^ Ultra filter device by centrifugation at 4000 g for 30–80 min, and PBS was added to the filter to continue centrifugation to obtain a suspension of 1.5 mL of prostate cancer exosomes (PCa-exos) [19]. A pipette was inserted into the bottom of the filter, and the sample was swept back and forth at the bottom to ensure complete exosome recovery. Afterwards, these exosomes were further purified using the Magcapture™ Exosome Isolation Kit PS (Fujifilm Wako, Osaka, Japan) according to the instructions. The obtained exosomes were stored at − 20 °C.

### Transmission electron microscopy

10 μL of PCa-exos were pipetted onto a copper network and precipitated for 1 min. Then the floating liquid was removed, and 1% phosphotungstic acid dyed droplets were added to the copper net to stain the exosomes for 5 min. After the excess dye was removed, the sample was dried and observed on an electron microscope.

### Nanoparticle size analysis

Exosomes from PC-3-M-2B4 or PC-3-M-1E8 were isolated and purified, and then resuspended in 3 ml of PBS. After shaking and mixing, the diameter of these exosomes was determined by using a Zetasizer Nano ZS9003030810 analyzer (Malvern Panalytical, Malvern, UK).

### Immunofluorescence staining

The macrophages in 96-well plates were washed with PBS 3 times and fixed at room temperature for 15 min using 200 μL of 4% paraformaldehyde. After blocking with 2% BSA for 30 min at room temperature, CD206 primary antibody (Abcam, USA) was added to the cells and incubated at 4 °C overnight. The next day, these cells were washed 3 times with PBS, and then photographed on a fluorescence microscope.

### Western blotting

Cells were lysed with RIPA buffer containing PMSF and phosphatase inhibitor (100:1:1) on ice for 5 min and then collected immediately into a 1.5 mL Eppendorf tube. After shaking for 30 min in an ice box, the cell debris was removed by centrifugation at 12,000 g for 10 min at 4 °C. The supernatant was collected and stored at − 20 °C; 30 μg of the total protein was applied to 10% SDS-PAGE and then transferred to a PVDF membrane. According to the molecular weight difference of the target molecules, the PVDF membrane was cut into multiple horizontal stripes containing specified target antigens. After blocking with 5% skim milk for 2 h, the proteins in the PVDF stripes were incubated with the following primary antibodies: β-actin, AKT, P-AKT, STAT3, and P-STAT3 (Abcam, USA), at a ratio of 1:1000 at 4 °C overnight. After washing with TBST 3 times, the corresponding secondary antibody was incubated for 2 h and the target protein was detected using ECL chemiluminescence.

### Enzyme-linked immunosorbent assay (ELISA)

We followed the instructions of the ELISA kit, and the samples from each group were added to a 96-well plate. The ELISA kits (Lianke Bio, China) were used to detect the concentration of specific cytokines. The OD value of each well was determined using a SpectraMax M4 Multimode Microplate reader (Molecular Devices, USA), and the concentration of cytokines was calculated by comparing their OD value with the corresponding standard curve.

### Flow cytometric analysis

After PCa cells were treated with GW4869, flow cytometric analysis was performed to detect PCa exosomes in the CM from these cells. We followed the instructions of the Exosome Isolation and Analysis Kit-Flow Cytometry (ab267478, Abcam). PCa exosomes were captured using a bead-bound anti-CD63 antibody, and then incubated with anti-CD9 PE for 1 h at 4 °C. Next, the amount of PCa exosomes in the CM was determined using a CytoFLEX (Beckman Coulter, Inc., Brea, CA, USA).

### Transwell assay

After a transwell chamber was placed in a 24-well plate, PC-3M-2B4 or PC-3M-1E8 cells (3 × 10^6^) were resuspended in 100 μl of CM from different macrophages and inoculated into the upper chamber which had been coated in matrigel. Next, 500 ul of RPMI-1640 media was added to the lower part of the chamber. The 24-well plate with transwells was placed in an incubator at 37 °C for 48 h. The cells in the transwell chamber were fixed and stained with 0.1% crystal violet for 10 min, washed three times with PBS, and observed under an inverted microscope.

### In vitro angiogenesis experiments

HUEVC and prostate cancer cells (PC-3M-2B4 or PC-3M-1E8) were resuspended with CM from different macrophages and mixed at a ratio of 1:1. Afterwards, 100 μl of these mixed cells (3.0 × 10^4^/well) were inoculated into a 96-well plate pre-coated with Matrigel, and then cultured for 24 h. The tubular structure of the endothelial cells was observed using an inverted microscope equipped with CCD optics and a digital analysis system (Olympus, Tokyo, Japan). The results were evaluated through counting the joint or vessel numbers in three fields per well.

### Subcutaneous tumorigenesis assay

Male BALB/c-nu mice (4 weeks old) were purchased from Changsha Laboratory Animal Co., Ltd. (Changsha, Hu Nan, China) and bred in the Laboratory Animal Resources at the Jiangxi Academy of Medical Sciences. Our study was approved by the Ethics Committee of Jiangxi Academy of Medical Sciences (protocol No. JXAMS-EWC-2019012).All animal experiments were performed in compliance with the guidelines for handling animal experimentation-based research in China and the ARRIVE guidelines. The PC-3M-1E8 cell line was cultured in RPMI-1640 (10% exosome-free serum). After mixing with 10 uM GW4869, 5 × 10^6^ PC-3M-1E8 cells were injected subcutaneously into the right flanks of these mice. Tumor development was observed every other day post-injection. After tumor formation, tumor volume was measured every five days and calculated using the equation: tumor volume (mm^3^) = shorter diameter^2^ × longer diameter/2, and the CD206 expression in tumor tissues was detected using immunohistochemistry.

### Immunohistochemistry

Mouse tumor tissues were prepared for generating 3 mm paraffin-embedded sections, and then immersed in citrate buffer (pH 6.0) to retrieve antigens. After endogenous peroxidase activity and nonspecific binding sites were blocked with 3% hydrogen peroxide or 5% bovine serum albumin, respectively, sections were incubated with CD206 antibody at 4 °C overnight. Then, the appropriate secondary antibodies were incubated for 1 h at room temperature. Diaminobenzidine tetrahydrochloride was used as a substrate to evaluate the immunoreactivity of these sections.

### Statistical analysis

Statistical analysis was performed using Prism 5.0 statistical software. The measurement data was expressed as the mean ± standard deviation, and a *t* test was used for comparison between the two groups with normal distributions and overall variance. A Mann–Whitney U test was used for comparison between two groups with non-normal distributions. *p* < 0.05 indicates that the difference was statistically significant.

## Results

### PCa exosome characteristics

**Fig. 1:**
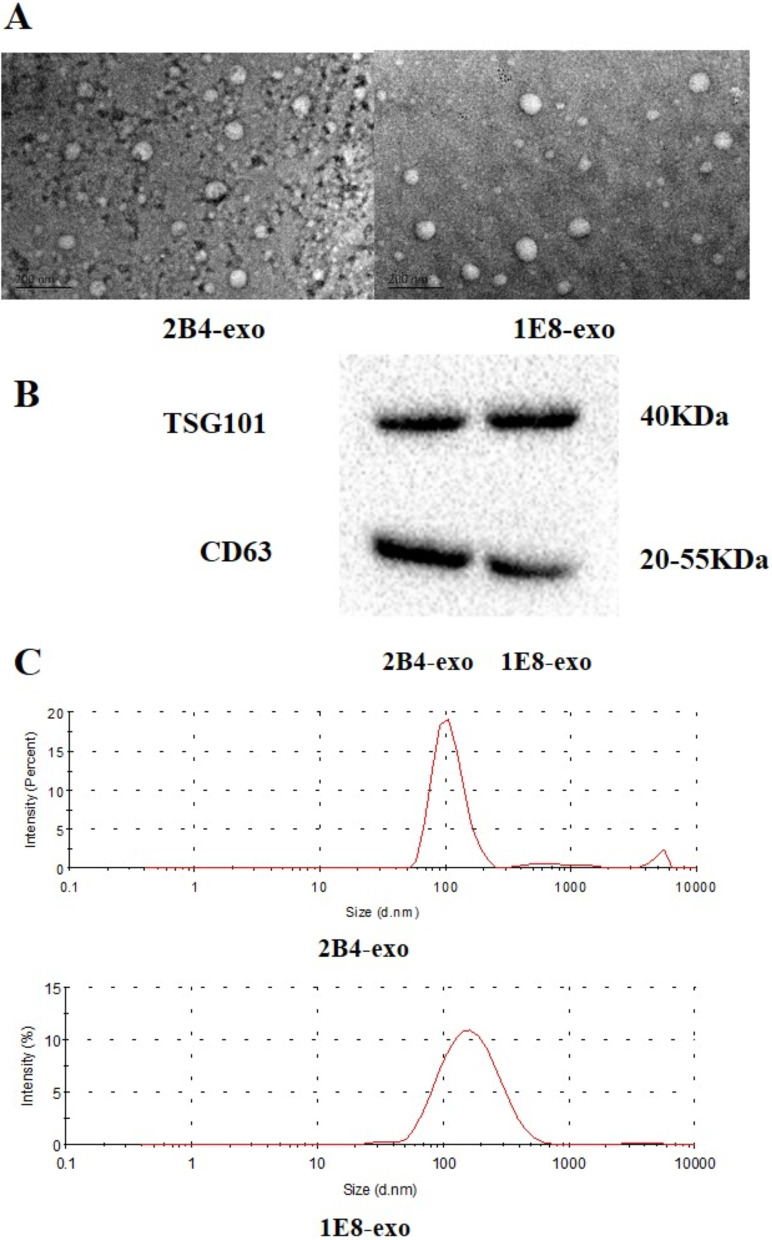
Identification of PCa exosomes. **A** Images of exosomes from PC-3-M-2B4 or PC-3-M-1E8 cells by transmission electron microscopy. **B** The expression of the specific molecular markers TSG101 and CD63 on exosome membranes were determined by Western blotting. The data was analyzed using Bio-Rad image Lab 3.0 (beta 3). Full-length blots are presented in Additional file 1: Fig. S1 and Raw data. **C** Diameter profiles of PCa exosomes. After Pca exosomes were obtained, the diameter of PCa exosomes was determined using a nanoparticle size analyzer

Exosomes are an important means for prostate cancer cells to regulate their microenvironment. In order to obtain PCa exosomes, two prostate cancer cell lines, 2B4 and 1E8, were cultured with exosome-free serum, and 80 ml of supernatant culture media was collected from each. The culture media was passed through a Millipore ultrafiltration tube (Amicon Ultra-15, Millipore, USA), concentrated down to 1 ml, and then these PCa exosomes were sorted using the magcapture ™ Exosome Isolation Kit PS (Fujifilm Wako, Osaka, Japan). The electron microscopy results show these PCa exosomes were round and had an obvious saucer-like structure. Nanoparticle size analysis showed that the diameters of 2B4-exos and IE8-exos were approximately 90–135 nm. In addition, the Western blot analysis indicated the expression of the secretion of specific molecular markers (TSG101 and CD63) (Fig. 1).

### PCa exosomes induced macrophages to differentiate into M2 macrophages

**Fig. 2:**
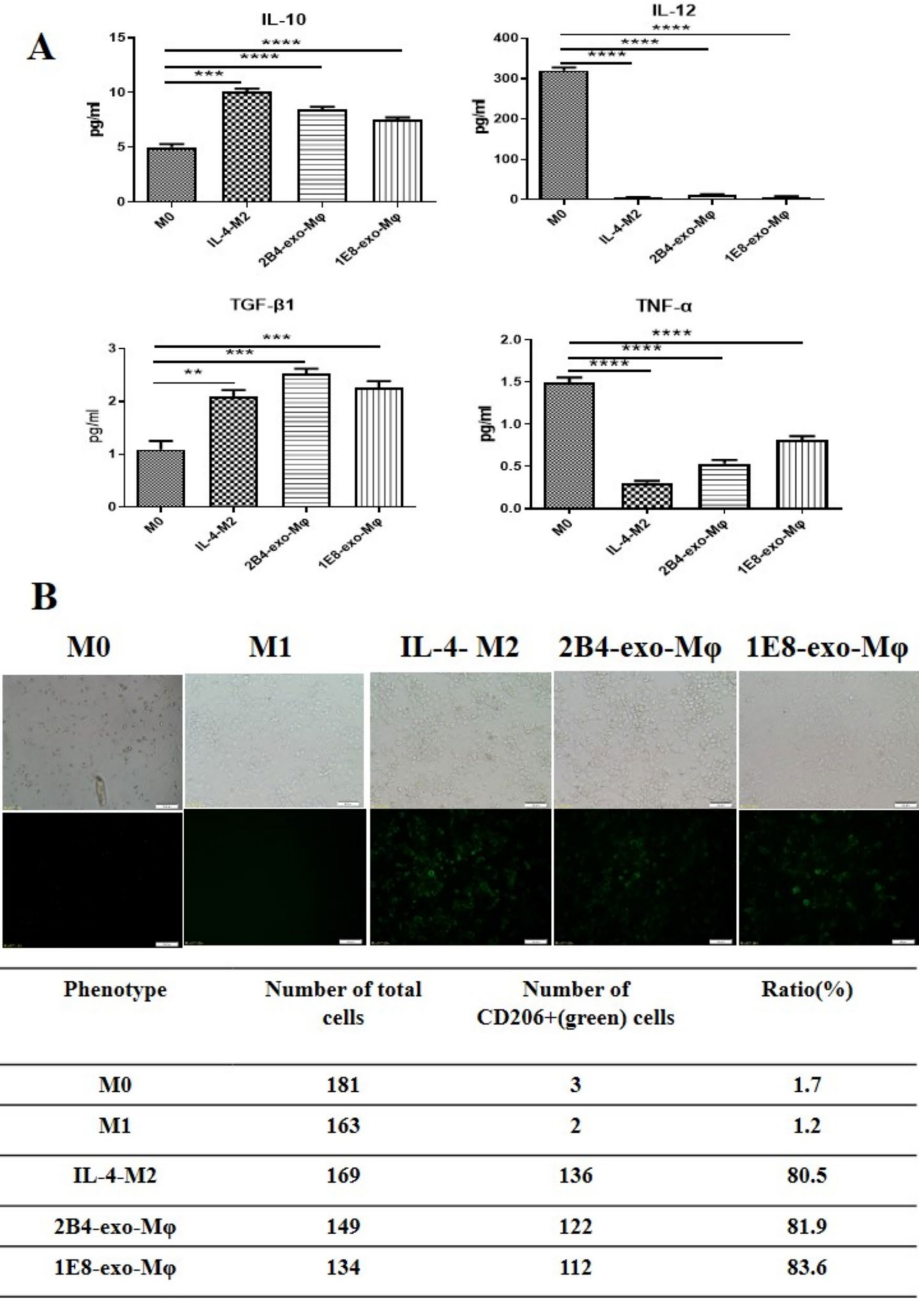
Macrophages induced by PCa exosomes show characteristics similar to M2 cells. **A** Cytokine profiles of macrophages induced by PCa exosomes. After treatment of PCa exosomes, the concentration of IL-12, TNF-α, TGF-β1, and IL-10 secreted by macrophages was measured by ELISA. *p ≤ 0.05, **p ≤ 0.01, ***p ≤ 0.001, ****p ≤ 0.0001. **B** PCa exosomes increased CD206 expression in macrophages. After THP1 cells were induced to macrophages with PMA, macrophages were co-cultured with PCa exosomes. The number of CD206+ macrophages was determined with an anti-CD-206-FITC (green) using a High Throughput Connotation of Imaging System (original magnification, 100×)

Current studies have suggested that tumor cells regulate the polarization process of macrophages through a variety of mechanisms. To understand the role of prostate cancer exosomes in macrophage polarization, we first induced THP1 cells into macrophages with 10 ng/ml PMA for 72 h [20], and then these macrophages were treated with IFN-γ (50 ng/mL), IL-4 (25 ng/mL), 2B4-exos (100 μg/ml), and IE8-exos (100 μg/ml) [21]. After culturing them for 48 h, we observed that, similar to the macrophages induced by IL-4, macrophages treated by 2B4-exos or IE8-exos significantly over-expressed CD206, a molecular marker of the M2 phenotype, IL-10 (M0 vs. 2B4-exo-Mφ, *p* < 0.0001; M0 vs.1E8-exo-Mφ, *p* = 0.0004) and TGF-β1 (M0 vs. 2B4-exo-Mφ, *p* = 0.0001; M0 vs. 1E8-exo-Mφ, *p* = 0.0004) (Fig. 2). These results confirmed that exosomes from prostate cancer cells could induce macrophages to differentiate into M2 cells.

### *GW4869 decreased the secretion of PCa exosomes and the number of CD206*+ *macrophages*

**Fig. 3:**
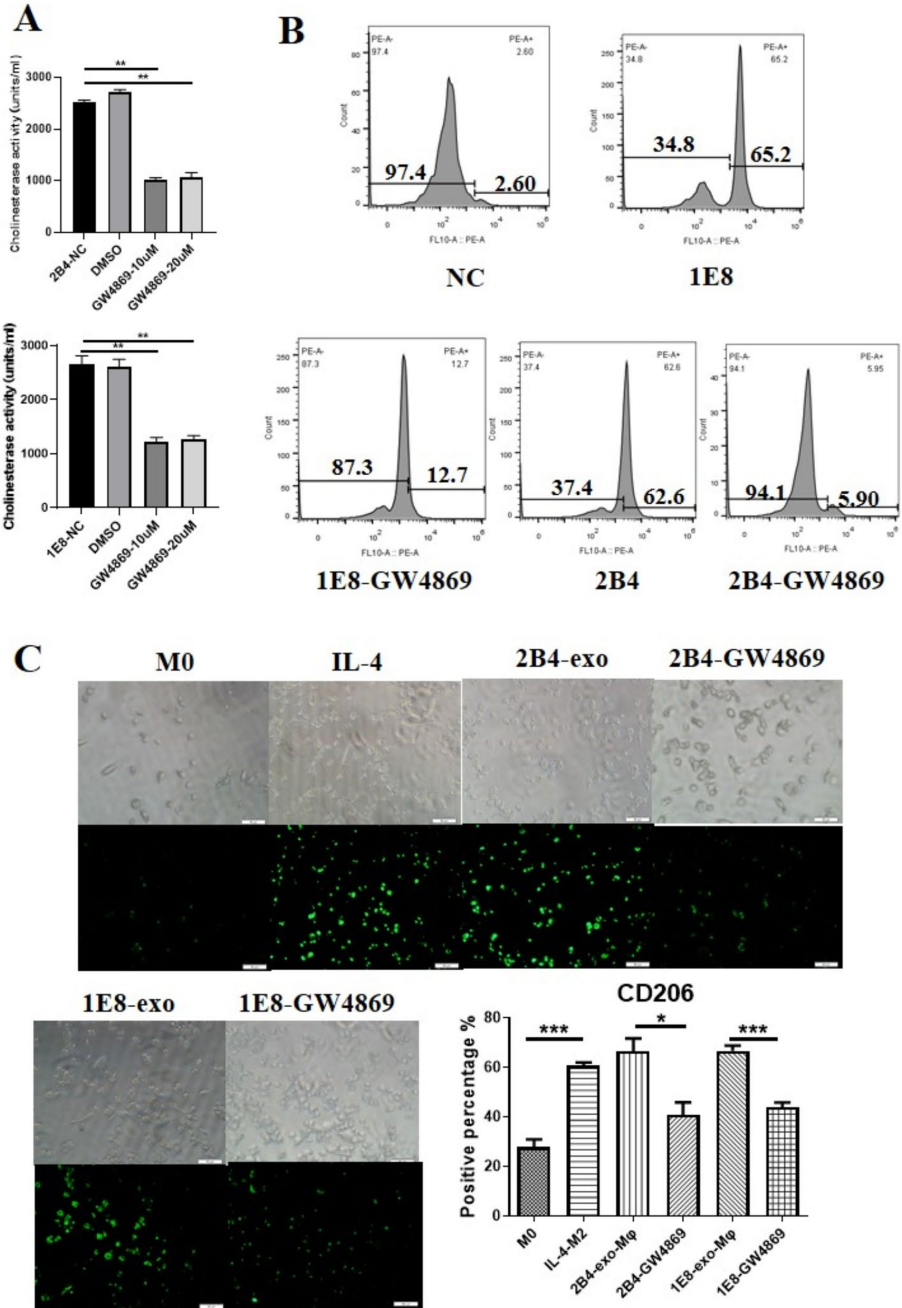
Effect of GW4869 on the secretion of exosomes and number of CD206+ macrophages. **A** Effect of GW4869 on AChE activity. After PCa cells were treated with GW4869 for 48 h, CM from PC-3-M-2B4 or PC-3-M-1E8 was collected, and then the AChE activity in the above CM was detected by ELISA. *p ≤ 0.05, **p ≤ 0.01, ***p ≤ 0.001, ****p ≤ 0.0001. **B** Effect of GW4869 on the release of PCa exosomes. After GW4869 treatment, the amount of exosomes in the CM from PC-3-M-2B4 or PC-3-M-1E8 cells was evaluated using flow cytometry. **C** CM from PCa cells treated with GW4869 reduced the CD206 expression in macrophages. After macrophages were co-cultured with CM-2B4-GW4869 or CM-1E8-GW4869, respectively, we determined the CD206 expression of subtypes of macrophages with an anti-CD-206-FITC antibody (green)

GW4869 is thought to inhibit neuraminidase activity in cells, thereby inhibiting the secretion of tumor cell exosomes. In order to understand the effect of GW4869 on prostate cancer cells, we treated the prostate cancer cell lines PC-3-M-2B4 and PC-3-M-1E8 with 10 and 20 μM GW4869, respectively. After 48 h, we measured the cholinesterase activity in the supernatant of the culture medium from these two cell lines. Cholinesterase is abundant in exosomes, and its activity is considered to represent the number of exosomes. As shown in Fig. 3A, the cholinesterase activity in the PCa cell culture supernatant decreased significantly after GW4869 treatment (GW4869-10uM/20uM vs. 2B4-NC, *p* = 0.0010/*p* = 0.0022; GW4869-10uM/20uM vs. 1E8-NC, *p* < 0.0001/*p* < 0.0001) which indirectly confirmed that GW4869 inhibited the secretion of PCa cell exosomes, and there was no observed difference between the 10 and 20 μM treatments. Subsequently, we further analyzed the amount of exosomes in the supernatant from PCa cells treated with 10 μM GW4869 using flow cytometric analysis. After treatment with GW4869, the number of exosomes from PC-3-M-2B4 or PC-3-M-1E8 cells decreased by 56.7% and 52.5%, respectively (Fig. 3B). Finally, we observed the effect of GW4869 on macrophage polarization mediated by prostate cancer cells. The results from Fig. 3C demonstrate that 10 μM GW4869 significantly decreased the number of CD206+ macrophages (2B4-GW4869 vs. 2B4-exo-Mφ*, p* = 0.0182*;* 1E8-GW4869 vs. 1E8-exo-Mφ, *p* = 0.0003), suggesting that GW4869 could inhibit macrophage differentiation into M2 cells through blocking the secretion of exosomes from PCa cells.

### Blocking the release of PCa exosomes with GW4869 reduced the tumor-promoting ability of tumor-associated macrophages

**Fig. 4:**
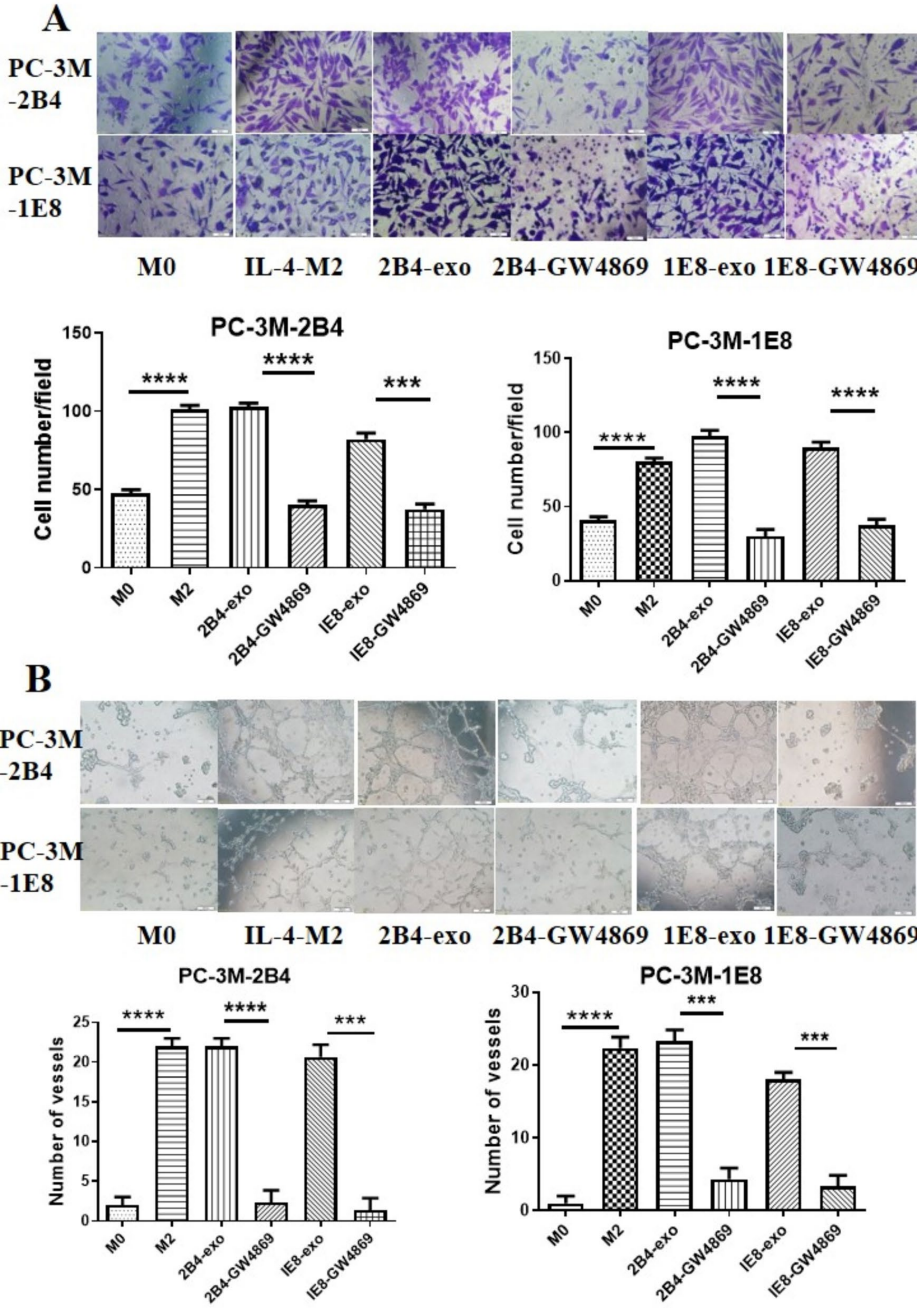
Blocking the release of PCa exosomes with GW4869 affected the tumor-promoting ability of tumor-associated macrophages. **A** Macrophages induced with CM from PCa treated with GW4869 decreased the invasive ability of PCa cells. Macrophages were incubated with PCa-exos, CM-2B4-GW4869/CM-1E8-GW4869, and IL-4 for 48 h. CM from each group was collected and added to PCa cells in transwell chambers. Cells in these transwell chambers were stained and observed from three randomly chosen fields (original magnification, 100×) 48 h later. **B** Macrophages induced with CM from PCa cells treated with GW4869 suppresses pro-angiogenic effects of PCa cells. HUVECs and PC-3M-2B4 or PC-3M-1E8 cells were inoculated into a 96 well plates pre-coated with matrigel, followed by the addition of CM from the above macrophages for 24 h. The tubular structures of endothelial cells were imaged with an inverted microscope equipped with CCD optics and a digital analysis system. Results were evaluated through counting the joint or vessel numbers in three fields per well (original magnification, 100×). *p ≤ 0.05, **p ≤ 0.01, ***p ≤ 0.001, ****p ≤ 0.0001

Macrophages in the tumor microenvironment promote the occurrence and development of tumors. To verify the effect of GW4869 on the function of macrophages regulated by PCa exosomes, we isolated and purified the exosomes from the supernatant of PCa cells treated with GW4869. Next, these exosomes were incubated with macrophages, and the conditioned media was collected to observe the effects on prostate cancer cells. Our data (Fig. 4A) showed that after treatment of GW4869, the supernatant of PCa cells significantly decreased the ability of macrophages in promoting PC-3-2B4 (2B4-GW4869 vs. 2B4-exo, *p* < 0.0001; 1E8-GW4869 vs.1E8-exo, *p* = 0.0001) and PC-3-1E8 cell invasion (2B4-GW4869 vs. 2B4-exo, *p* < 0.0001; 1E8-GW4869 vs. 1E8-exo, *p* = 0.0001). Likewise, the ability of macrophages in promoting PC-3-2B4 (2B4-GW4869 vs. 2B4-exo, *p* < 0.0001; 1E8-GW4869 vs.1E8-exo, *p* = 0.0001) and PC-3-1E8 angiogenesis (2B4-GW4869 vs. 2B4-exo, *p* = 0.0001; 1E8-GW4869 vs. 1E8-exo, *p* = 0.0002)  also was significantly decreased (Fig. 4B).

### Blocking the release of PCa exosomes with GW4869 inactivated the AKT and STAT3 signaling pathways in macrophages

**Fig. 5:**
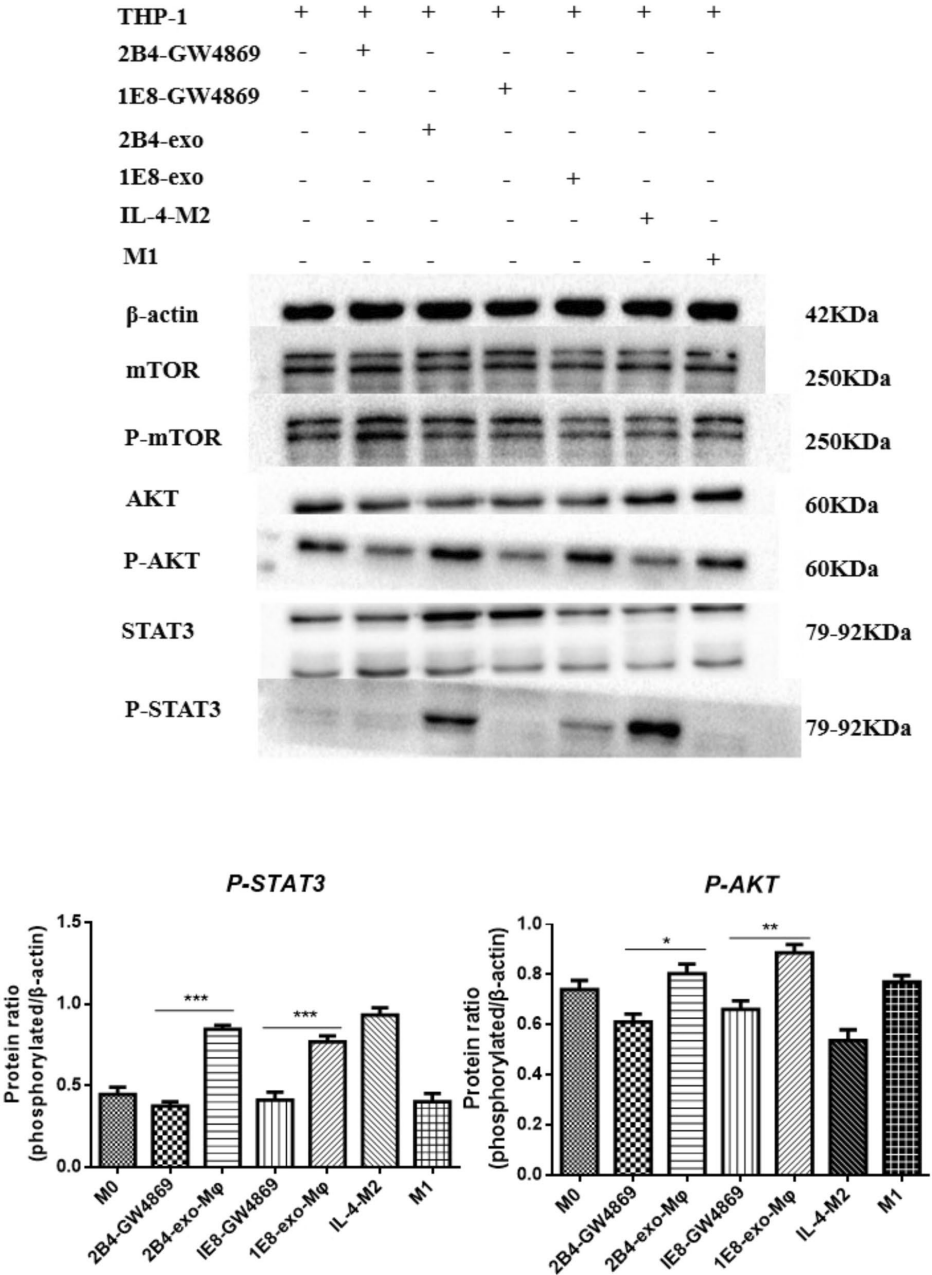
Blocking the release of PCa exosomes with GW4869 inactivates the AKT and STAT3 signaling pathways in macrophages. Macrophages were co-cultured with PCa exosomes or CM-2B4-GW4869/CM-1E8-GW4869 for 48 h. Cells from each group were harvested and lysed with RIPA buffer, and then the protein concentration was determined using the Bio-Rad protein assay. We used 20 µg of the cell lysates to analyze the activity of the AKT/STAT3 signaling pathways in different macrophage cells by Western blotting. The data was analyzed using Bio-Rad image Lab 3.0 (beta 3). Full-length blots are presented in Additional file 1: Fig. S2 and Raw data

The underlying mechanisms through which GW4869 affects the differentiation of tumor-associated macrophages in prostate cancer still remains unclear. Therefore, we induced macrophages with culture supernatant from PCa cells treated by GW4869 or PCa exosomes, and then analyzed their molecular changes. The results showed that exosomes mainly induced M2 differentiation of macrophages by activating the AKT and STAT3 signaling pathways. In contrast, AKT and STAT3 were inhibited in macrophages treated with PCa supernatant after the GW4869 treatment, suggesting that GW4869 inhibited the activation of the AKT (2B4-GW4869 vs. 2B4-exo-Mφ, *p* = 0.0182; 1E8-GW4869 vs. 1E8-exo-Mφ, *p* = 0.0092) and STAT3 (2B4-GW4869 vs. 2B4-exo-Mφ, *p* < 0.0001; 1E8-GW4869 vs. 1E8-exo-Mφ, *p* = 0.0005) signaling pathways by reducing the secretion of PCa exosomes (Fig. 5).

### GW4869 inhibited the differentiation of tumor-associated macrophages into the M2 phenotype in vivo

**Fig. 6:**
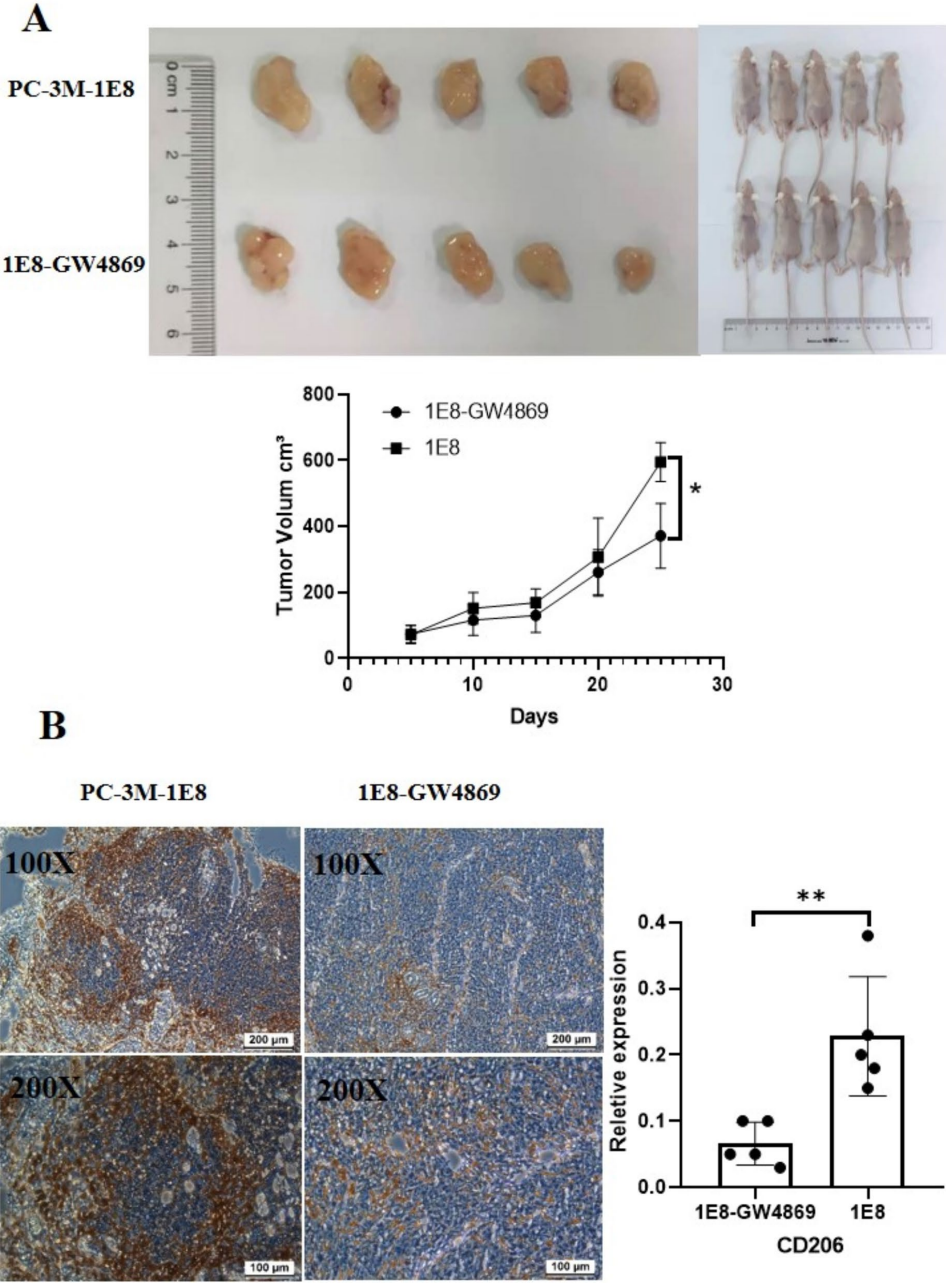
GW4869 inhibited the differentiation of tumor-associated macrophages into M2 phenotype in vivo. **A** The effect of GW4869 on the growth of prostate cancer in tumor xenograft mice (n = 5). After the subcutaneous injection of 5 × 10^6^ PC-3-M-1E8 cells or PC-3-M-1E8 cells treated with GW4869 in nude mice, tumor volume was measured every five days, where tumor volume (mm^3^) = shorter diameter^2^ × longer diameter/2. **B** GW4869 reduced number of CD206+ macrophages in PCa tissues. After tumor formation, CD206 expression in tumor tissue was determined using immunohistochemical analysis. *p ≤ 0.05, **p ≤ 0.01, ***p ≤ 0.001, ****p ≤ 0.0001

In order to further confirm the effect of GW4869 on tumor-associated macrophages in prostate cancer, we established a nude mouse model of prostate cancer. After PCa cells were treated with GW4869, they were injected into nude mice to observe the effects of GW4869 on macrophage differentiation. As shown in Fig. 6A, compared with the control group, the tumor size of the GW4869 treatment group was significantly reduced (Day = 25, *p* = 0.0071). Moreover, the number of CD206+ macrophages in the tumor tissue from the GW4869 treatment group decreased by 3.45 fold (*p* = 0.0053) (Fig. 6B). These results further confirmed that GW4869 inhibited the differentiation of tumor-associated macrophages into the M2 phenotype.

## Discussion

Macrophages in a tumor microenvironment have a characterized phenotype and function as M2 cells, and these TAMs promote the secretion of tumor growth factors and enhance the invasion and metastasis of prostate tumor cells [22, 23]. Significantly, tumor-derived exosomes, as a main source of environmental signals, drive macrophages to acquire the M2 phenotype [24, 25]. Therefore, the blockade of exosome release is able to inhibit the high efficiency communication between tumor cells and stromal cells. In this study, we demonstrated that PCa-exos could effectively induce macrophages to differentiate into M2 cells through the AKT and STAT3 pathways. Importantly, we found that GW4869 effectively inhibited the generation of exosomes in PCa cells and blocked the induction of M2 macrophages by prostate cancer cells in vitro and in vivo. As a result, treatment with GW4869 attenuated the pro-tumor ability of these TAMs induced by PCa-exos.

Our previous studies have shown that CM from prostate cancer cells can induce blood monocytes to differentiate into M2 cells [21]. We speculated that, in addition to some cytokines in CM, exosomes from PCa cells may also play an important role in macrophage polarization. To this end, we obtained exosomes from the PCa cell lines PC-3M-2B4 and 1E8 cells by PS affinity chromatography and analyzed their characteristics. As shown in Fig. 1, most of the exosomes from prostate cancer cells were round, with typical saucer-like structures, a diameter of approximately 40–160 nm, and the specific molecular markers CD63 and tgs101 were expressed on the exosome membrane. These results show that through the above methods, we obtained PCa exosomes that met our experimental requirements.

In order to understand the effects of PCa exosomes on macrophages, we induced THP1 cells into macrophages with PMA and cocultured the above exosomes with these induced macrophages. Our data showed that under the action of exosomes, CD206, TGF-β, and IL-10 in macrophages were significantly increased, while TNF-α decreased (Fig. 2). The increased expression of IL-10, TGF, and CD206 is considered to be an important marker of M2 macrophages. Our results confirmed that PCa exosomes have an induction effect similar to IL-4 that can promote M2 differentiation of macrophages.

Essandoh et al. reported that after treatment with GW4869, RAW264.7 cells significantly decreased the number of exosomes produced, which seriously affected the secretion of cytokines such as TNF alpha, IL-1beta and IL-6 [26]. In order to further understand the effect of GW4869 on prostate associated macrophages, first, we evaluated the effect of GW4869 on PCa exosome generation. We treated PC-3M-2B4 and PC-3M-1E8 cells with 10 μM GW4869 for 48 h, and then collected CM from these cells. Since cholinesterase is often located in exosomes and is considered an indirect indicator of exosome content [27], we determined the activity of cholinesterase in the above CM. As was expected, 10 μM GW4869 had a significant inhibitory effect on exosome secretion, up to 65% (Fig. 3A). We also analyzed the amount of exosomes in the supernatant from PCa cells treated with 10 μM GW4869 using flow cytometric analysis. After treatment with GW4869, the number of exosomes from PC-3-M-2B4 or PC-3-M-1E8 cells decreased by 57.7% and 52.5%, respectively (Fig. 3B). Second, we observed the effects of GW4869 on macrophage polarization mediated by prostate cancer cells. The results from Fig. 3C confirmed that 10 μM GW4869 significantly decreased the number of CD206+ macrophages, suggesting that GW4869 could inhibit macrophage differentiation into M2 cells through blocking the secretion of exosomes from PCa cells. Lastly, we incubated macrophages with PCa exosomes or CM from PCa cells treated GW4869, respectively. After 48 h, we collected CM from these macrophages and investigated its effects on prostate cancer cells. As shown in Fig. 4, macrophages induced by PCa-exos could significantly promote the invasion and angiogenesis of prostate cancer cells, but GW4869-CM had no such effect. Our results suggested that GW4869 could affect the differentiation of tumor-associated macrophages and inhibit tumor progression in vitro.

We tried to determine the mechanism of PCa-exos/GW4869 on macrophages. Our data showed (Fig. 5) that PCa-exos, like IL-4, could significantly activate the AKT and STAT3 signaling pathways in macrophages, while the phosphorylation levels of these two signaling proteins were inhibited in GW4869-CM. The AKT and STAT3 pathways are closely related to the polarization process of macrophages [28–31], and our data further confirmed that prostate cancer cells can activate the AKT and STAT3 pathways through exosomes to cultivate M2 macrophages. This regulation of the tumor microenvironment can be inhibited by GW4869. Exosomal specific molecules have been reported to mediate macrophage polarization by the PTEN/PI3Kγ pathway [32] or gp130/STAT3 signaling [33]. Nevertheless, this result was more likely to be the result of the joint action of multiple molecules in exosomes. In this respect, GW4869 should receive increased attention as a tool for the regulation of the tumor microenvironment.

Finally, we observed the effect of GW4869 on macrophage polarization using a nude mouse model. The results of in vivo experiments further confirmed that the number of M2 macrophages in tumor tissue and the size of tumors in vivo were significantly reduced in our GW4869 treated prostate cancer model nude mice (Fig. 6). Some researchers have reported that treatment with GW4869 can inhibit tumor growth in nude mice, which may be related to the regulation of GW4869 on the tumor microenvironment through the inhibition of the secretion of tumor cell exosomes [34, 35]. Our data further confirm that GW4869 could inhibit the education of M2 macrophages in prostate cancer, and then inhibit the progression of tumors in vivo.

In conclusion, our study shows that exosomes derived from prostate cancer cells can induce macrophages into the M2 subtype by activating the AKT/STAT3 signaling pathway and promote the occurrence and progression of PCa, but this process can be inhibited by GW4869. GW4869 may play an important role in the clinical treatment of prostate cancer, especially advanced prostate cancer, and even have similar effects in the treatment of other tumors, but this needs more experimental and clinical studies to confirm.

## Supplementary Information

**Additional file 1.** Supplementary materials.

## Data Availability

The datasets generated and/or analysed during the current study are available in supplementary file raw data.

## Abbreviations

TME: Tumor microenvironment
TAM: Tumor-associated macrophages
PCa: Prostate cancer
ELISA: Enzyme-linked immunosorbent assay
MMP: Matrix metalloproteinase
STAT: Signal transducer and activator of transcription
PCa-exo: Prostate cancer exosome
CM-2B4-GW4869: Conditioned media from PC-3-M-2B4 cells treated with GW4869
CM-1E8-GW4869: Conditioned media from PC-3-M-1E8 cells treated with GW4869
