# Supplementary material for: Blockade of exosome generation by GW4869 inhibits the education of M2 macrophages in prostate cancer
Source: BMC Immunol. 2022 Aug 8;23:37. doi: 10.1186/s12865-022-00514-3 (PMC9361607; doi:10.1186/s12865-022-00514-3)
Supplement: Supplementary file 1 — Additional file 1. Supplementary materials. [file 12865_2022_514_MOESM1_ESM.docx]

**Figure 1. Identification of Pca exosomes**

A.Images of exosomes from PC-3-M-2B4 or PC-3-M-1E8 cells by transmission electron microscopy.



1E8-exo





2B4-exo

Fig S1. **The detection of TSG101 and CD63 on exosome membranes.** After PCa exosomes were obtained, the expression of the specific molecular markers TSG101 and CD63 on exosome membranes were determined using Western blotting.

Lane 1 2

2B4-exo 1E8-exo

**TSG101**
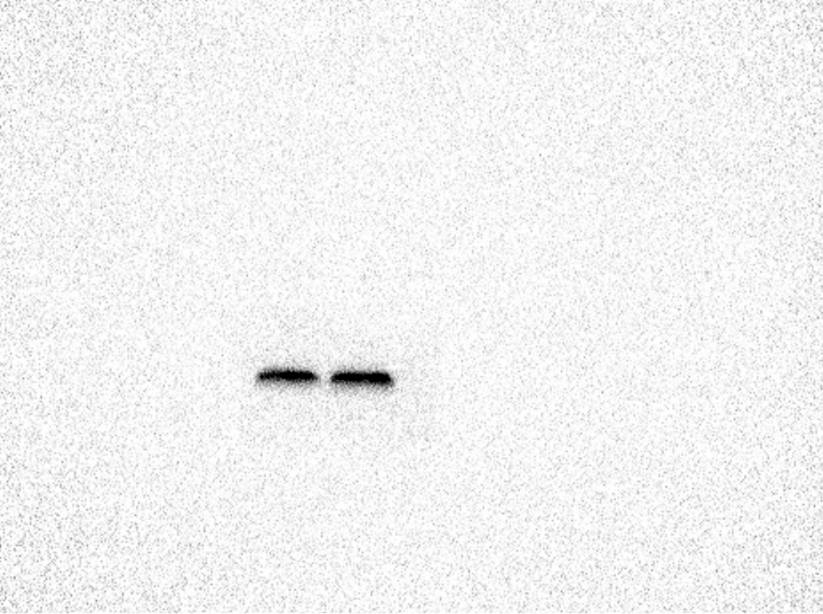
 **40KDa**

Exposure_1.0sec


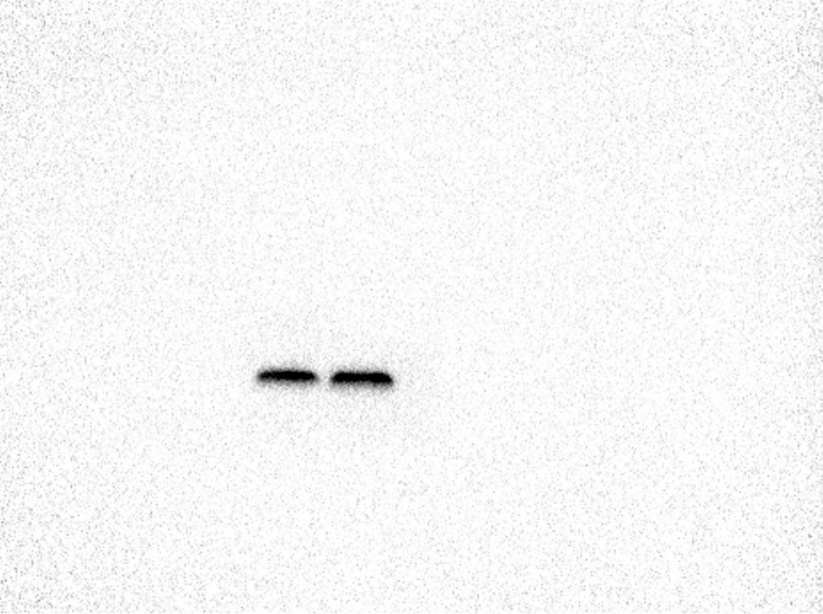


Exposure_4.1sec


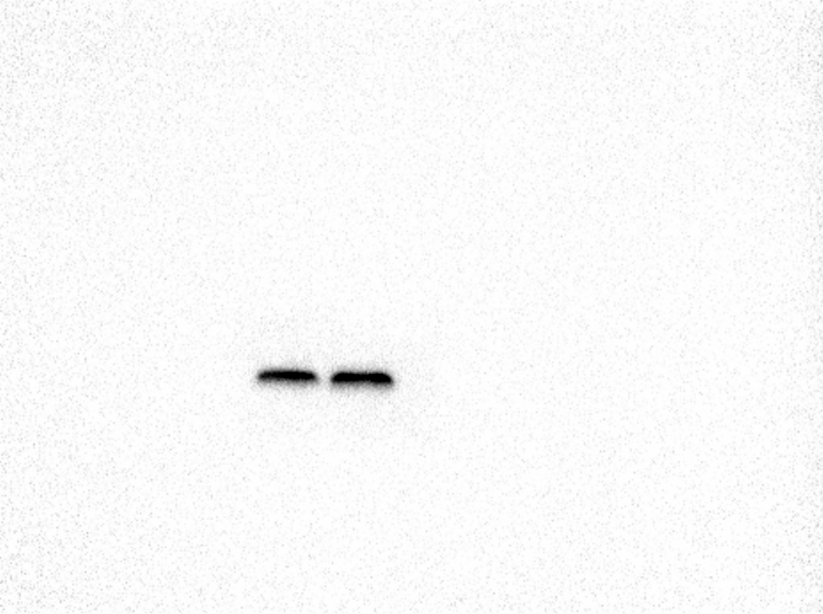


Exposure_8.8sec

Lane 1 2

1E8-exo 2B4-exo

**CD63**
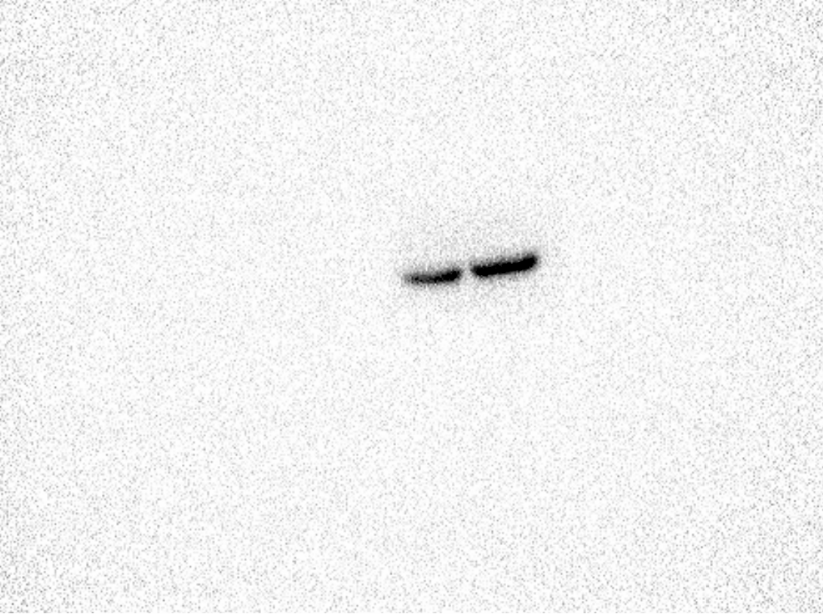
 **20-55KDa**

Exposure_1.0sec


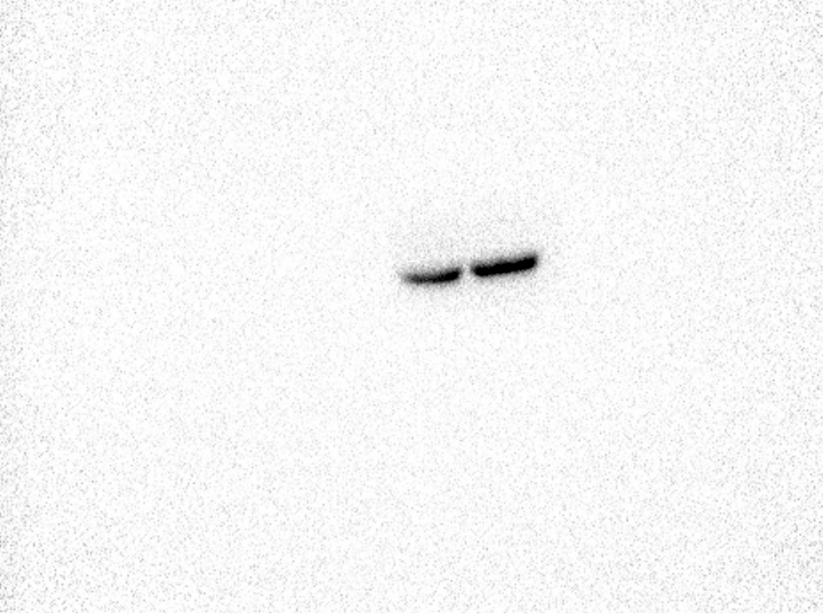


Exposure_4.1sec


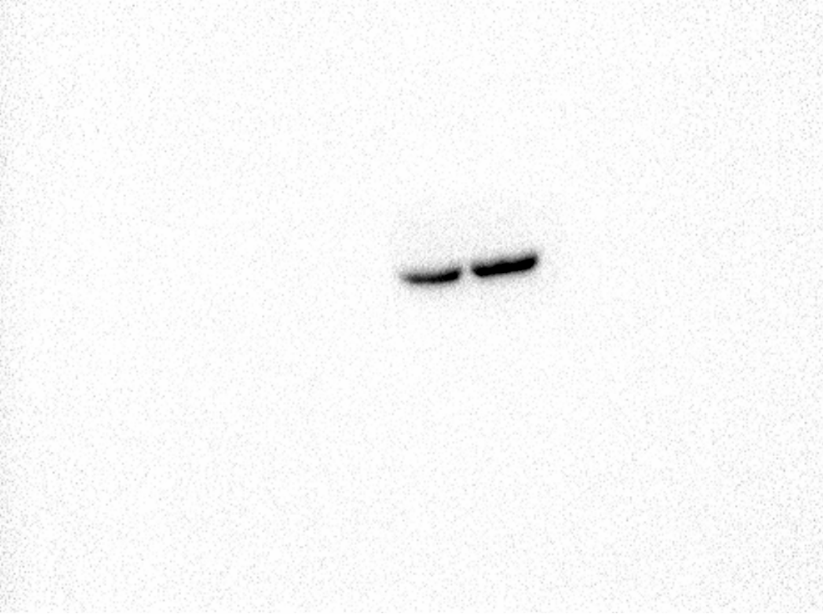


Exposure_8.8sec

C. Diameter profiles of Pca exosomes. After Pca exosomes were obtained, the diameter of Pca exosomes was determined using a nanoparticle size analyzer.

Table 1 Diameter profiles of PC-3-M-1E8 exosomes.

Table 2 Diameter profiles of PC-3-M-2B4 exosomes.

**Figure 2. Macrophages induced by Pca exosomes show characteristics similar to M2 cells.**

A. Cytokine profiles of macrophages induced by PCa exosomes. After treatment of PCa exosomes, the concentration of IL-12, TNF-α, TGF-β1, and IL-10 secreted by macrophages was measured by ELISA. * P < 0.05; ** P < 0.01.

Table 3 The concentration of IL-10(pg/ml)

| Sample name | M1 | IL-4-M2 | 2B4-exo-Mφ | 1E8-exo-Mφ |
| --- | --- | --- | --- | --- |
|  | 5 | 10 | 8.8 | 7.1 |
|  | 4.5 | 10.6 | 8.6 | 7.5 |
|  | 5.5 | 9.7 | 7.94 | 7.9 |

Table 4 The concentration of IL-12(pg/ml)

| Sample name | M1 | IL-4-M2 | 2B4-exo-Mφ | 1E8-exo-Mφ |
| --- | --- | --- | --- | --- |
|  | 305 | 2 | 9 | 10 |
|  | 325 | 5 | 15 | 7 |
|  | 330 | 7 | 10 | 3 |

Table 5 The concentration of TNF-α(pg/ml)

| Sample name | M1 | IL-4-M2 | 2B4-exo-Mφ | 1E8-exo-Mφ |
| --- | --- | --- | --- | --- |
|  | 1.37 | 2.3 | 2.5 | 2.2 |
|  | 1.1 | 2.1 | 2.7 | 2.5 |
|  | 0.8 | 1.9 | 2.4 | 2.1 |

Table 6 The concentration of TGF-β1 (pg/ml)

| Sample name | M1 | IL-4-M2 | 2B4-exo-Mφ | 1E8-exo-Mφ |
| --- | --- | --- | --- | --- |
|  | 1.37 | 0.3 | 0.6 | 0.9 |
|  | 1.5 | 0.35 | 0.55 | 0.8 |
|  | 1.6 | 0.27 | 0.45 | 0.75 |

B. PCa exosomes increased CD206 expression in macrophages. After THP1 cells were induced to macrophages with PMA, macrophages were co-cultured with PCa exosomes. The number of CD206+ macrophages was determined with an anti-CD-206-FITC (green) using a High Throughput Connotation of Imaging System (original magnification, 100×).


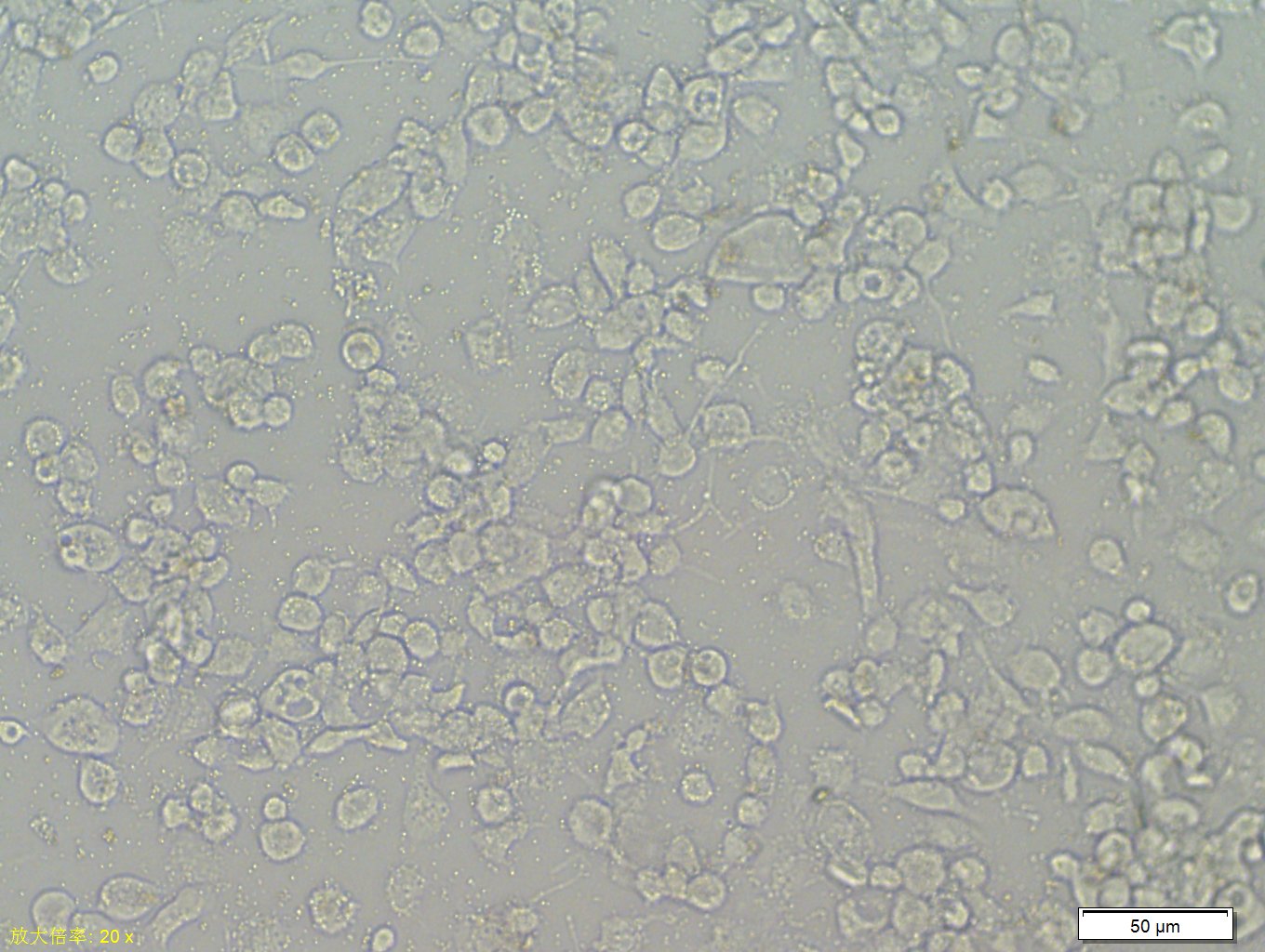


M0


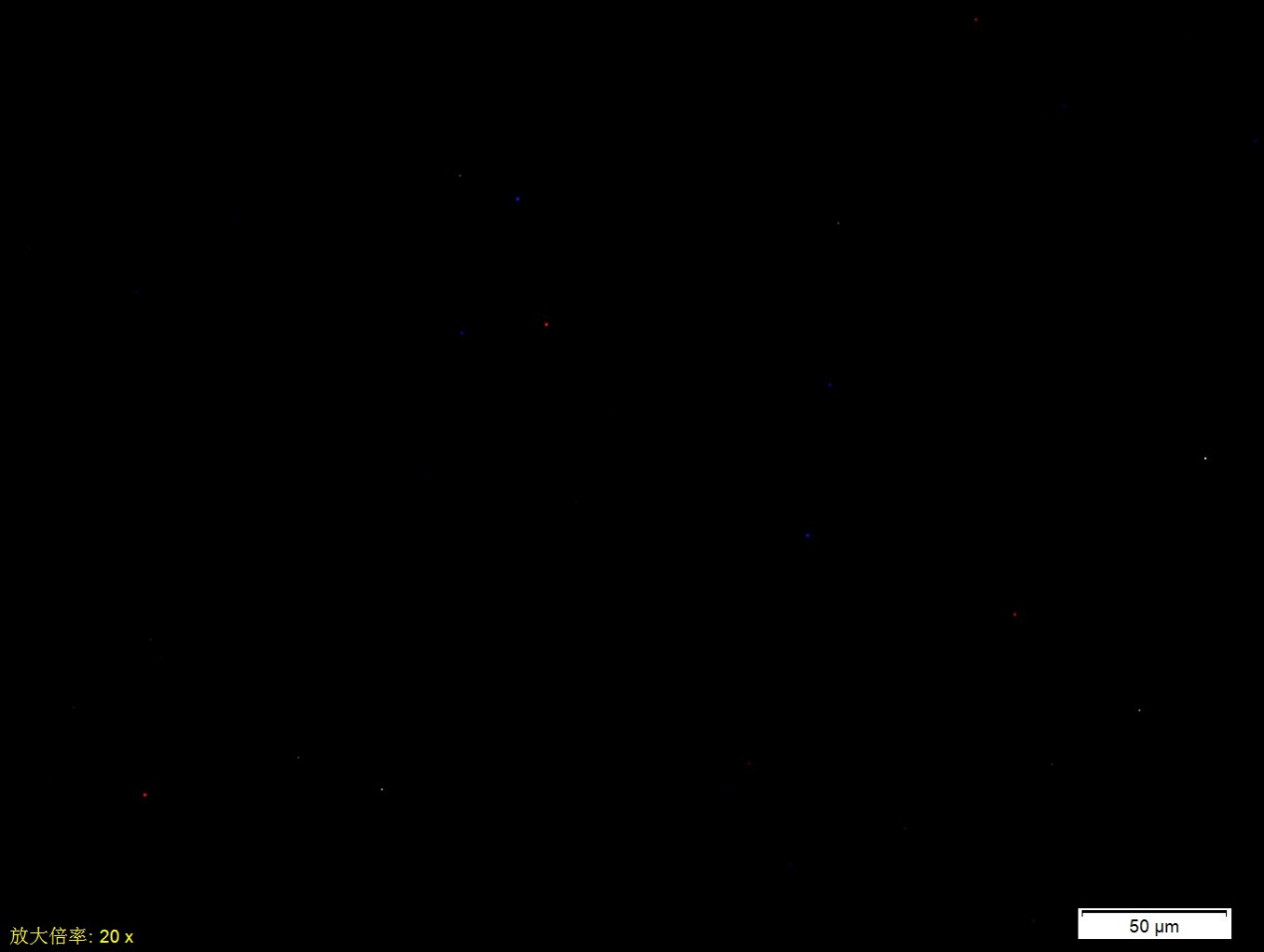


M0 (CD-206-FITC)


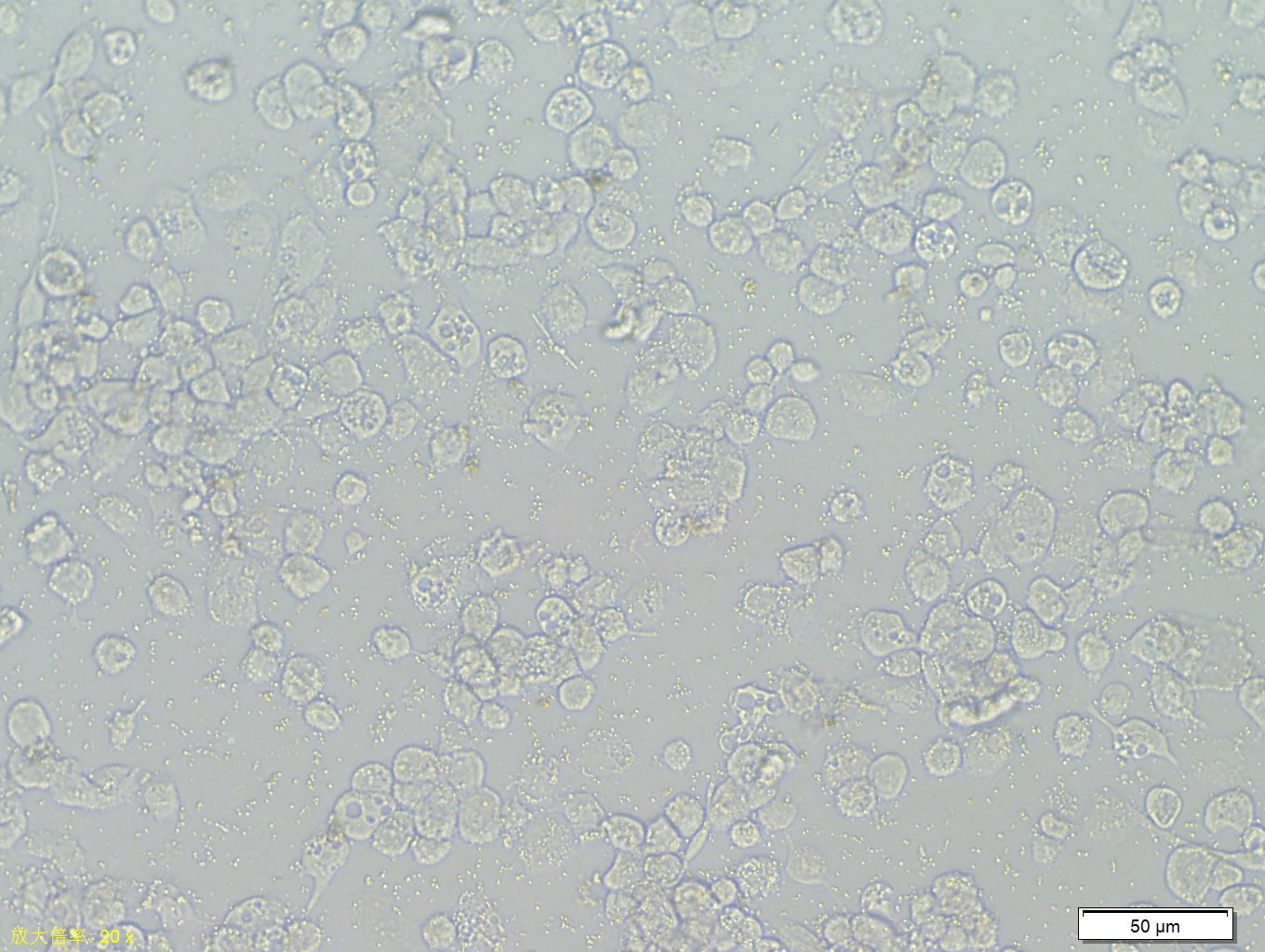


M1


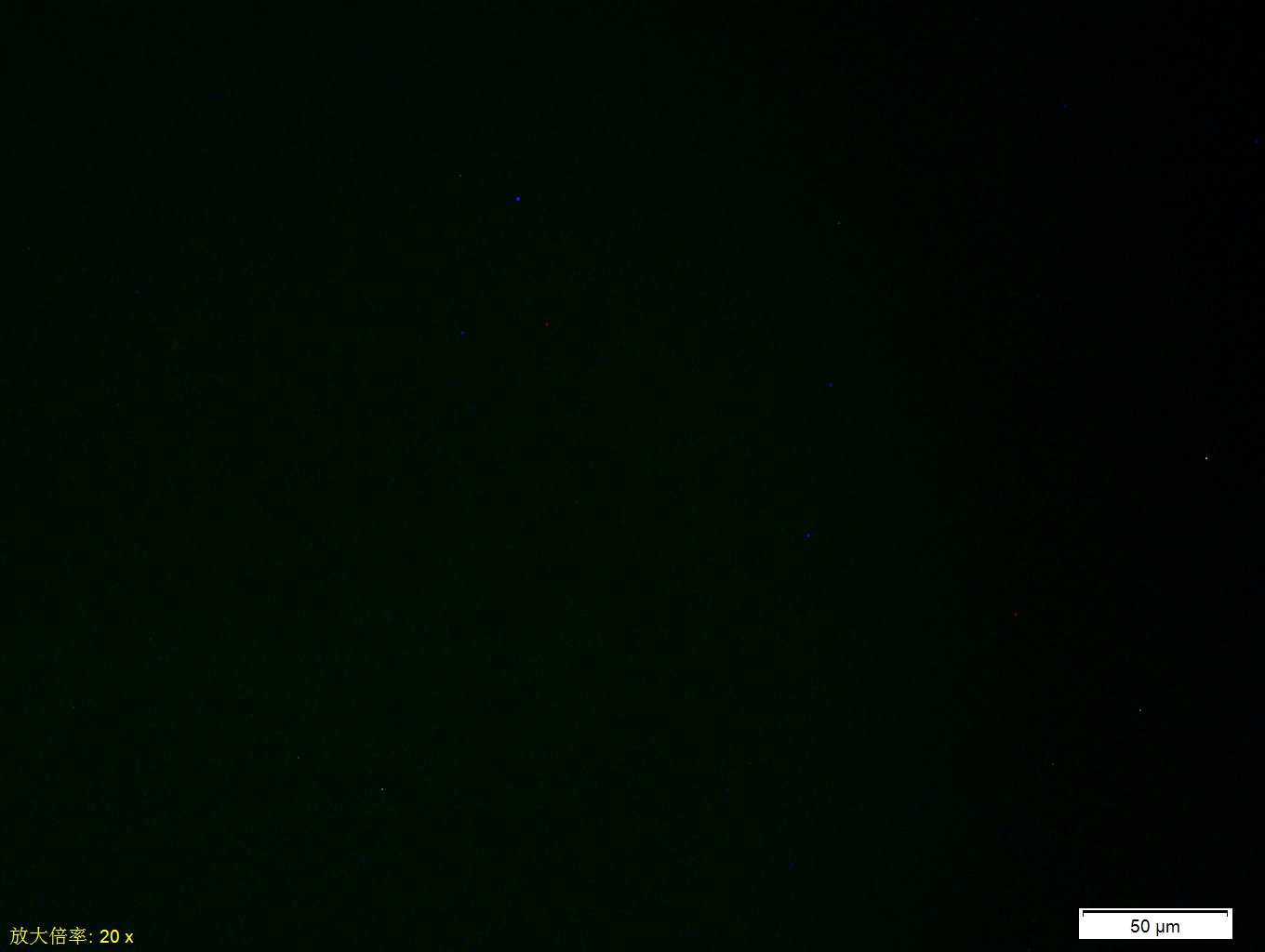


M1 (CD-206-FITC)


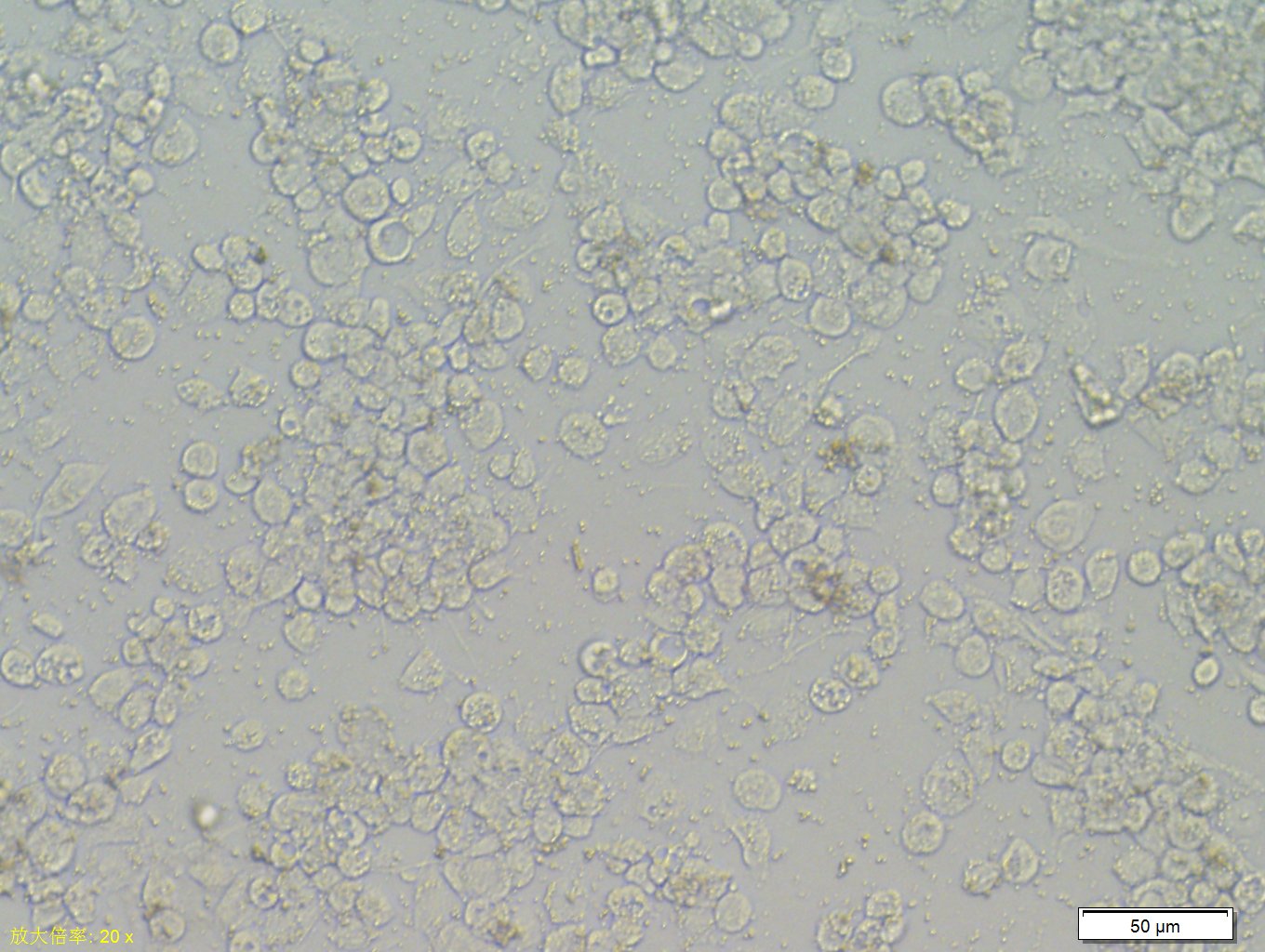


IL-4-M2


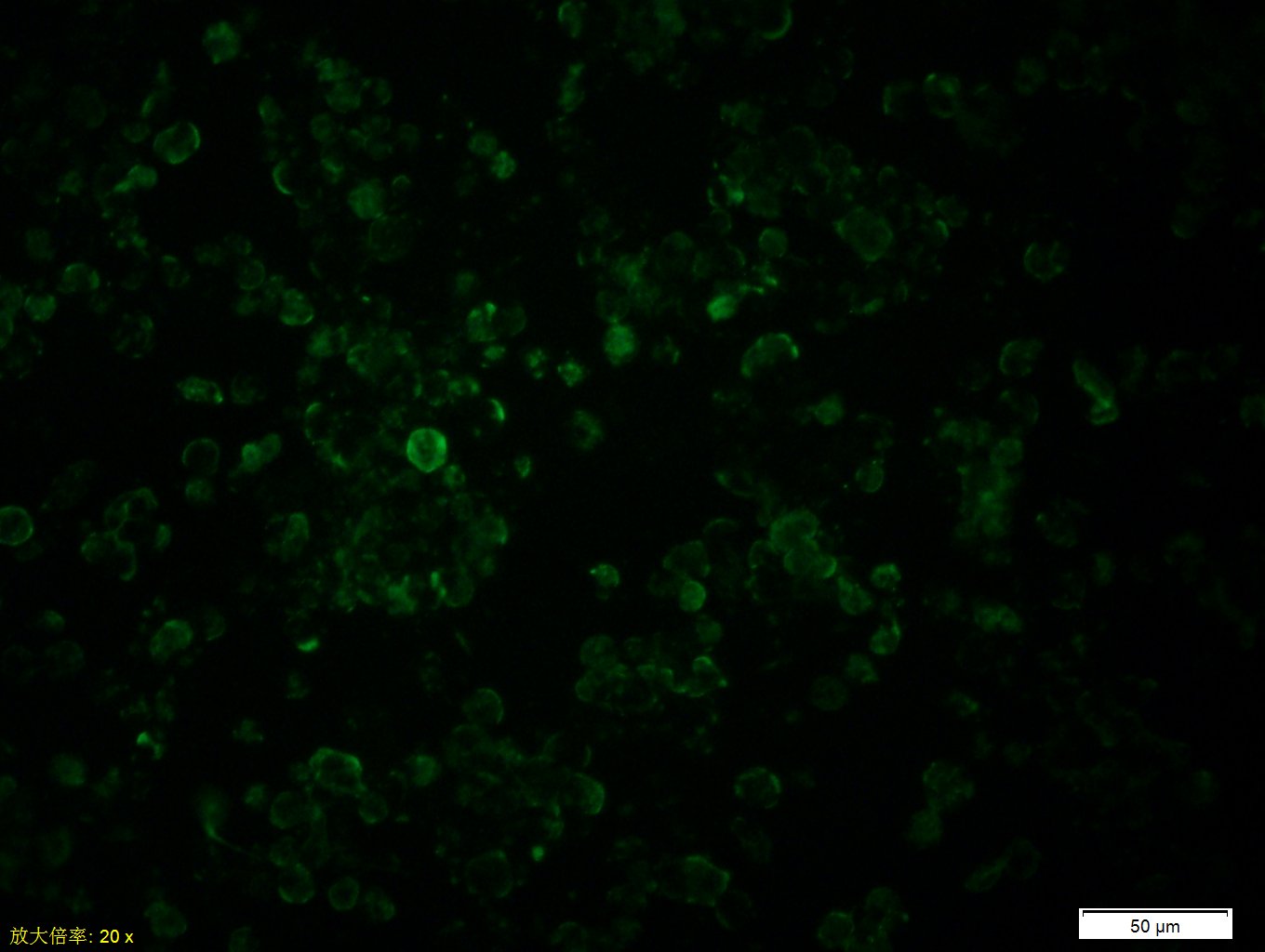


IL-4-M2 (CD-206-FITC)


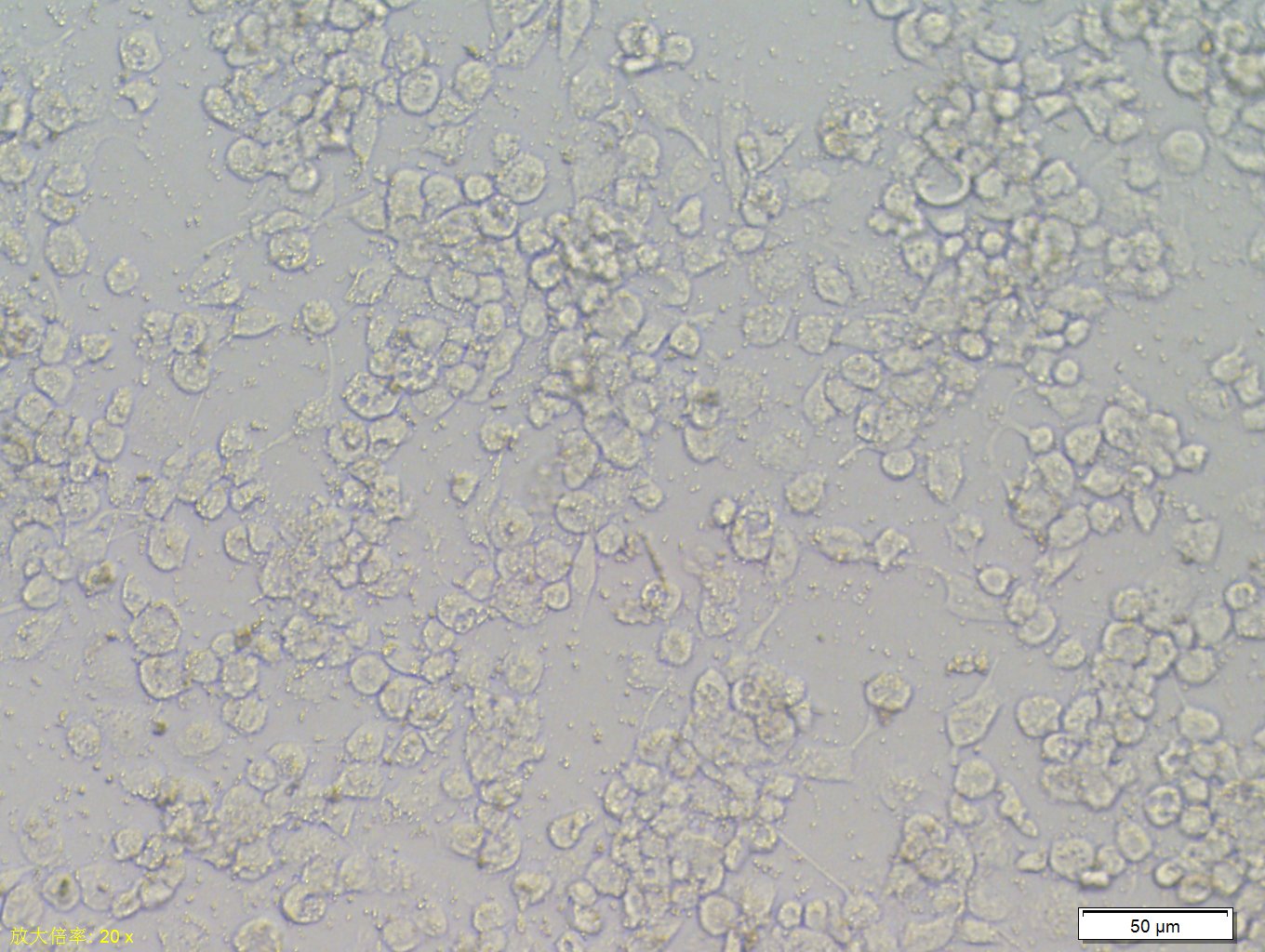


2B4-exo-Mφ


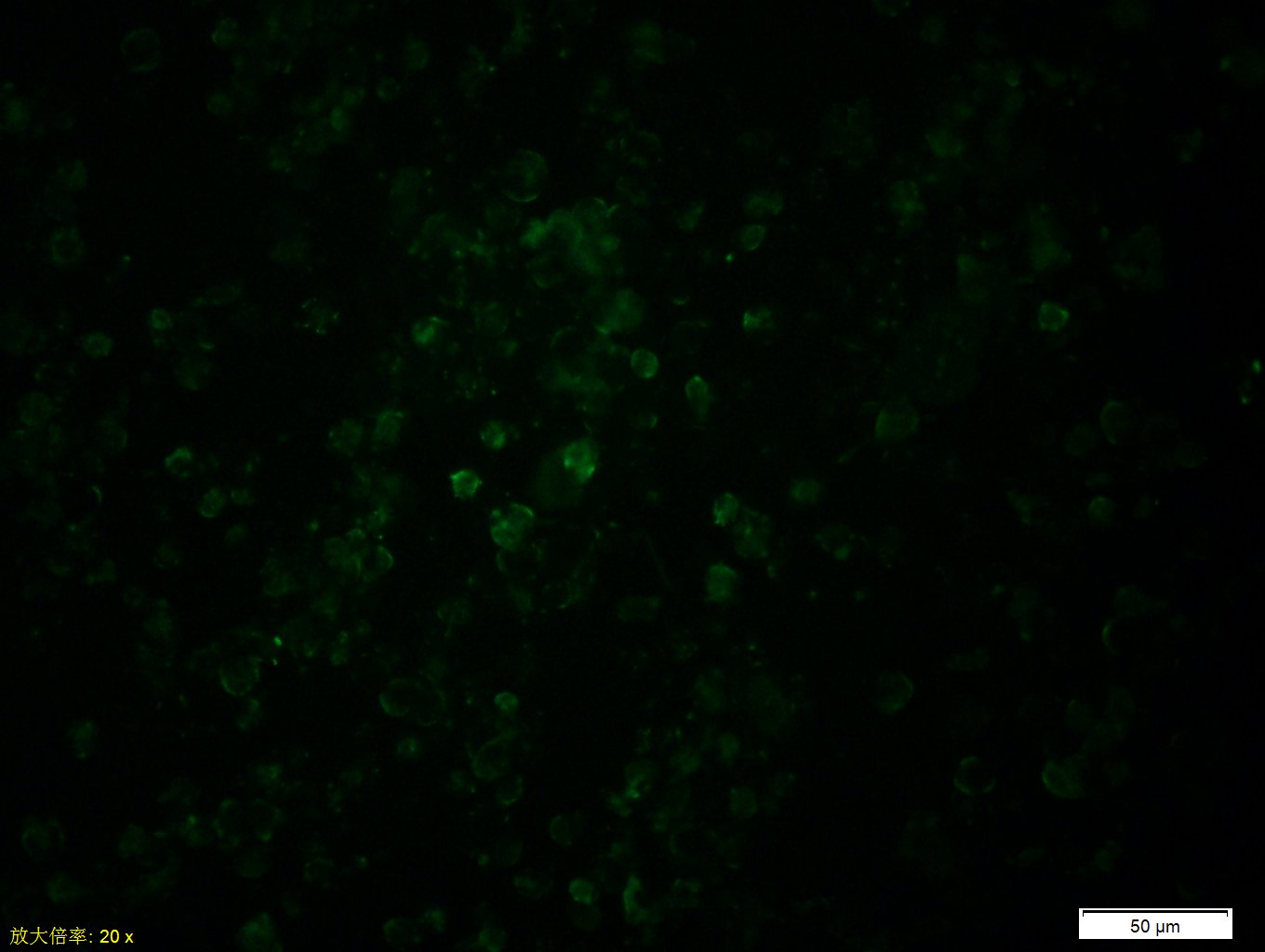


2B4-exo-Mφ (CD-206-FITC)


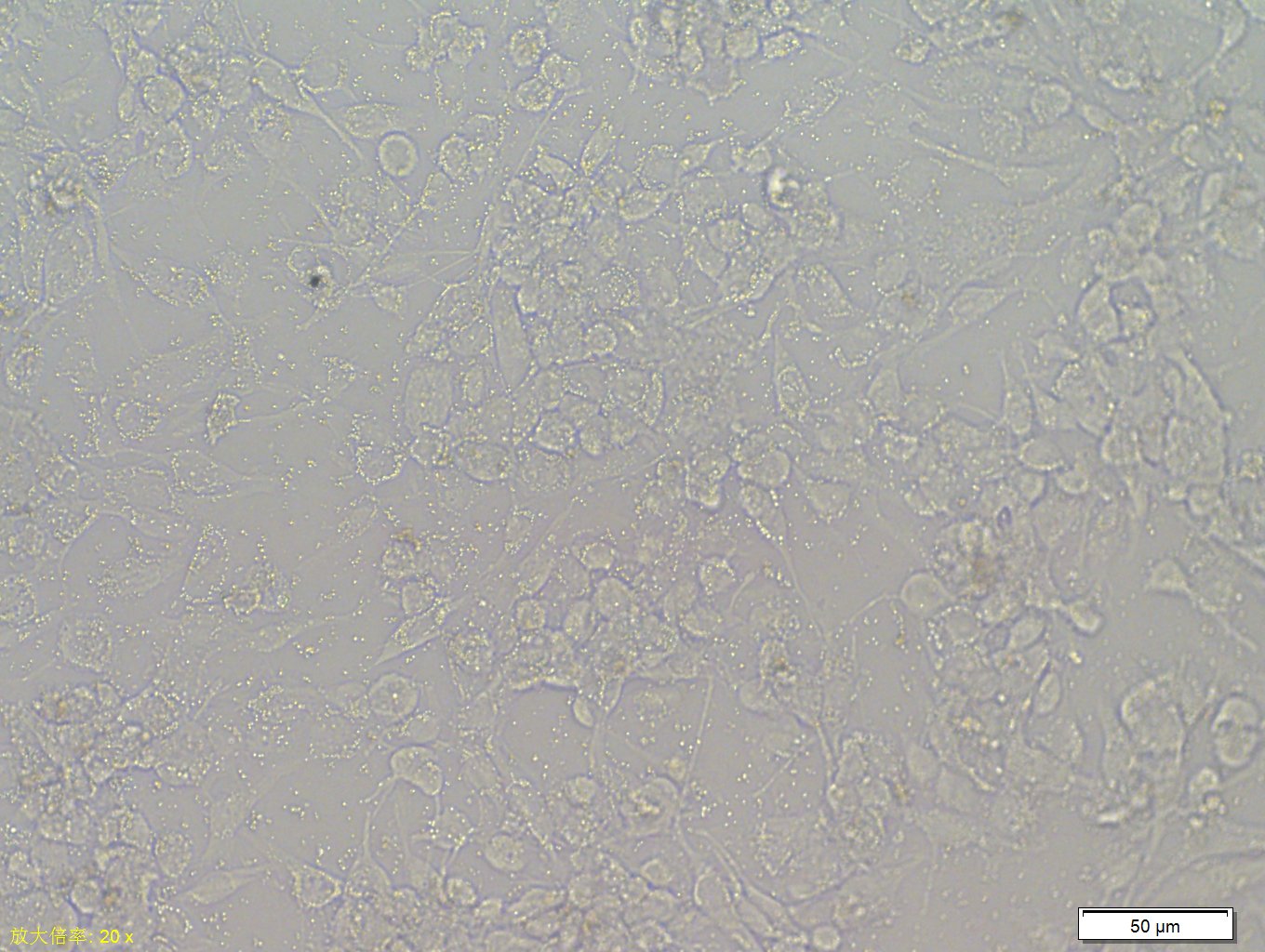


IE8-exo-Mφ


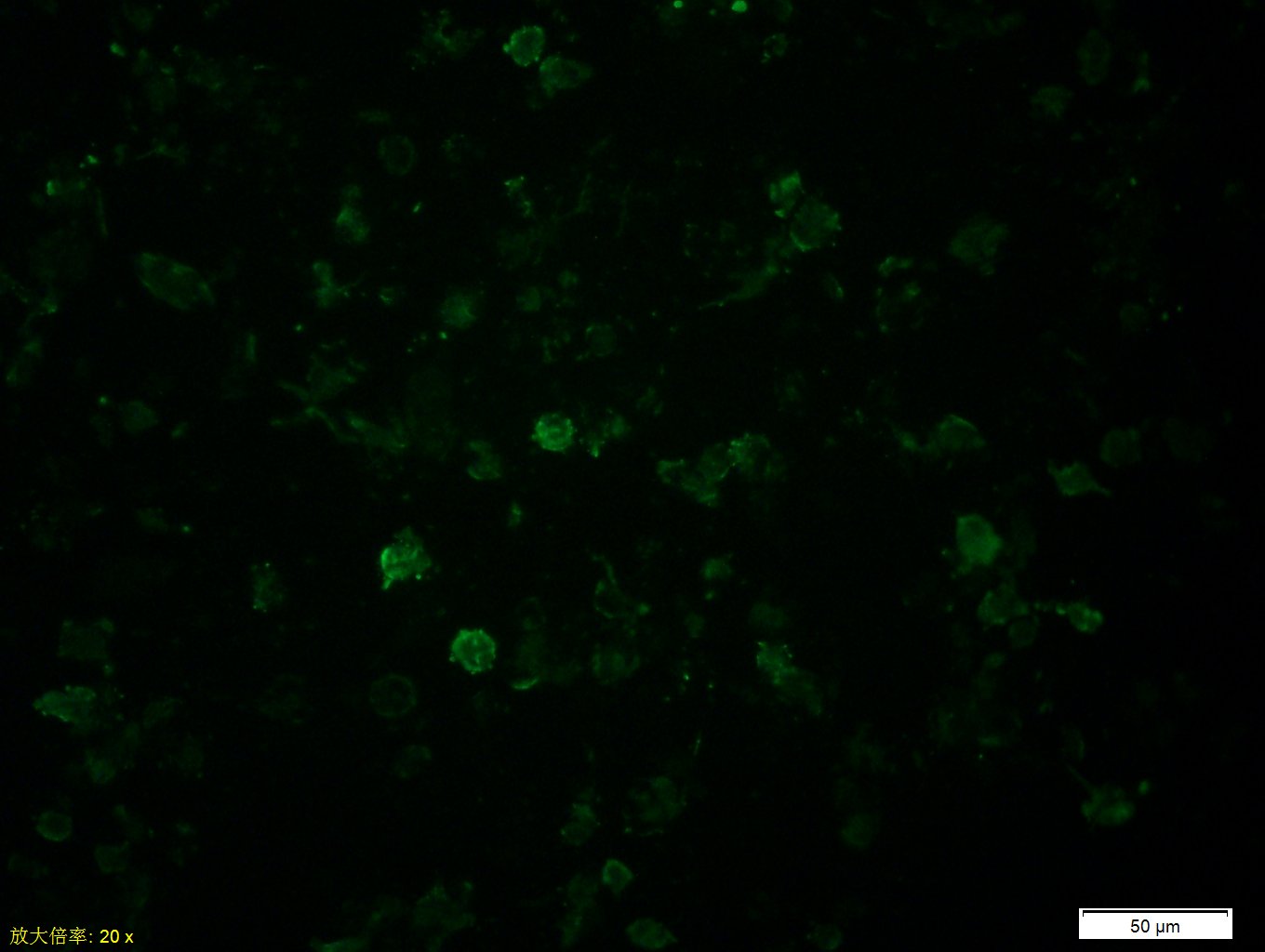


IE8-exo-Mφ (CD-206-FITC)


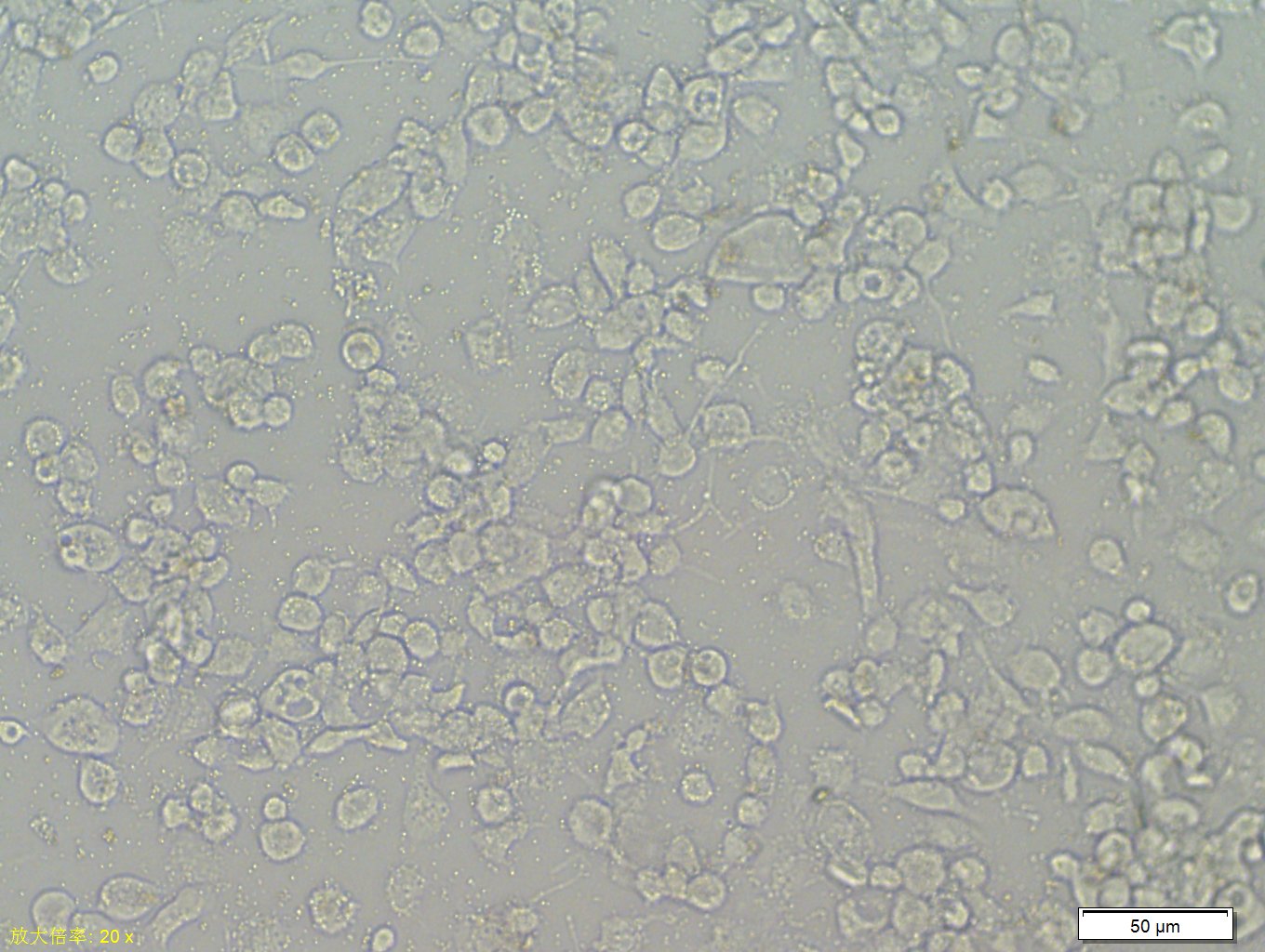


**Figure 3. Effect of GW4869 on the secretion of exosomes and number of CD206+ macrophages**

A. Effect of GW4869 on AChE activity. After PCa cells were treated with GW4869 for 48 h, CM from PC-3-M-2B4 or PC-3-M-1E8 was collected, and then the AChE activity in the above CM was detected by ELISA. * P < 0.05; ** P < 0.01.

Table 7 Effect of GW4869 on AChE activity in PC-3-M-1E8 cells

| Sample name | 1E8-NC | DMSO | GW4869-10uM | GW4869-20uM |
| --- | --- | --- | --- | --- |
|  | 2795 | 2728 | 1238 | 1264 |
|  | 2516 | 2417 | 1104 | 1219 |
|  | 2797 | 2662 | 1295 | 1213 |
|  | 2529 | 2643 | 1256 | 1366 |

Table 8 Effect of GW4869 on AChE activity in PC-3-M-2B4 cells

| Sample name | 2B4-NC | DMSO | GW4869-10uM | GW4869-20uM |
| --- | --- | --- | --- | --- |
|  | 2470 | 2693 | 1046 | 1018 |
|  | 2541 | 2747 | 986 | 1131 |

B. Effect of GW4869 on the release of PCa exosomes. After GW4869 treatment, the amount of exosomes in the CM from PC-3-M-2B4 or PC-3-M-1E8 cells was evaluated using flow cytometry.


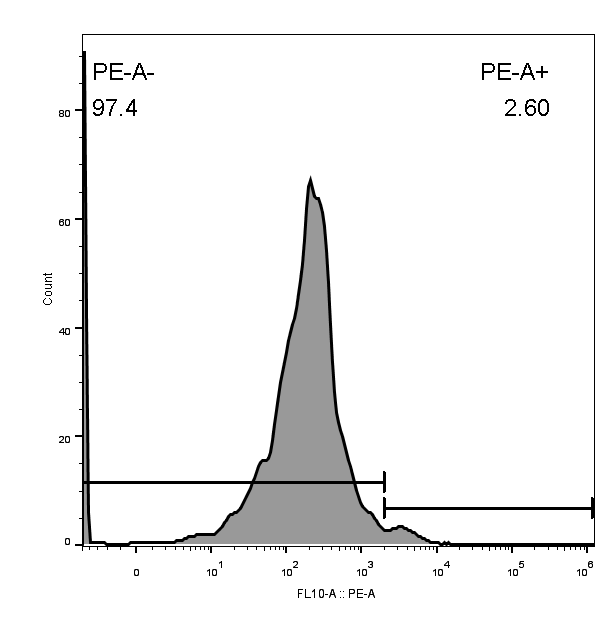


NC.fcs


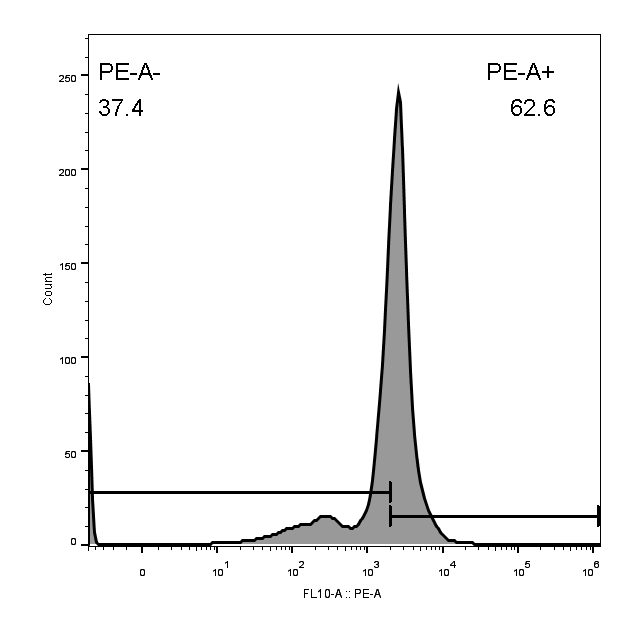


2B4.fcs


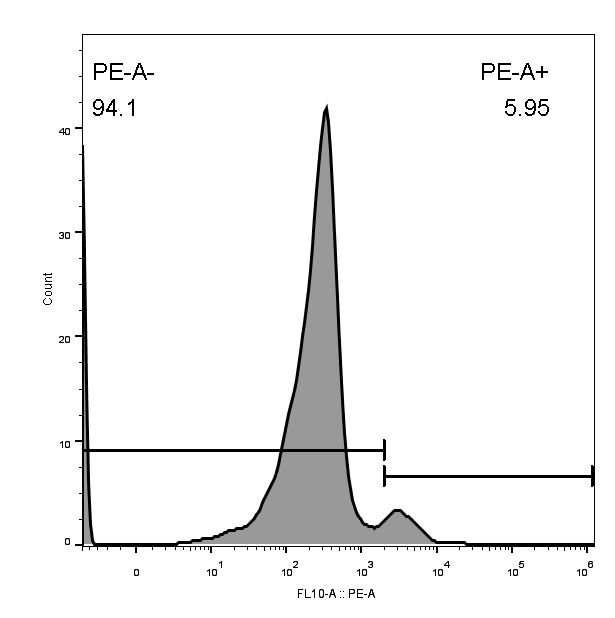


2B4-GW4869.fcs


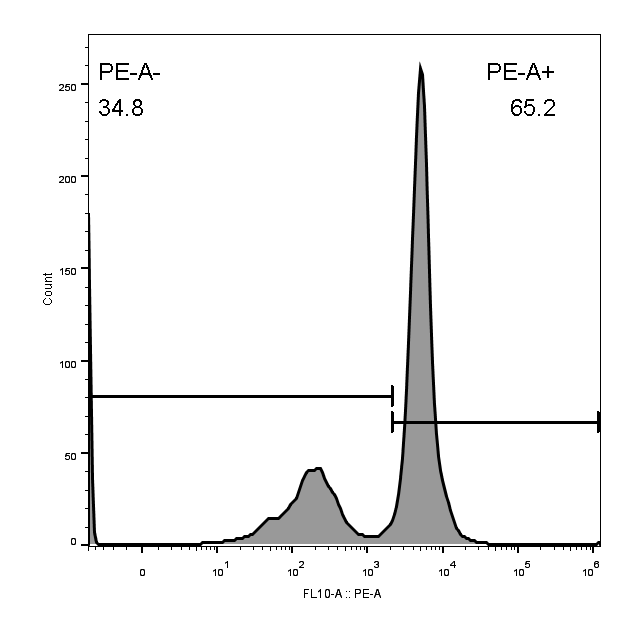


1E8.fcs


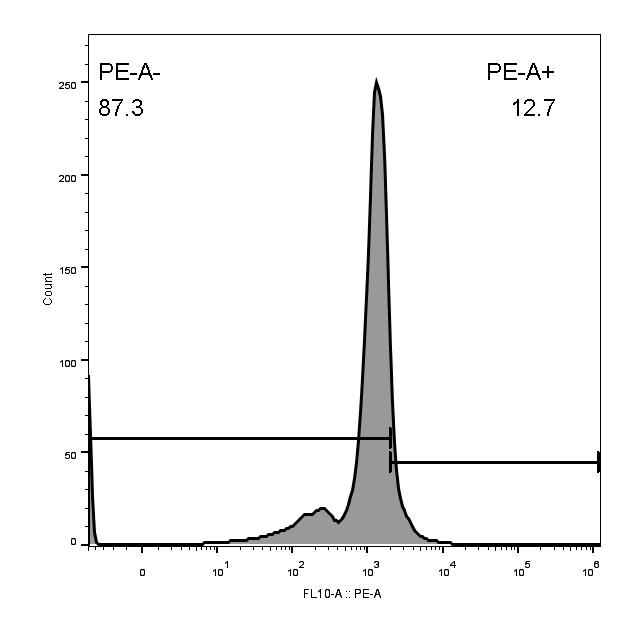


1E8+GW4869.fcs

C. CM from PCa cells treated with GW4869 reduced the CD206 expression in macrophages. After macrophages were co-cultured with CM-2B4-GW4869 or CM-1E8-GW4869, respectively, we determined the CD206 expression of subtypes of macrophages with an anti-CD-206-FITC antibody (green).


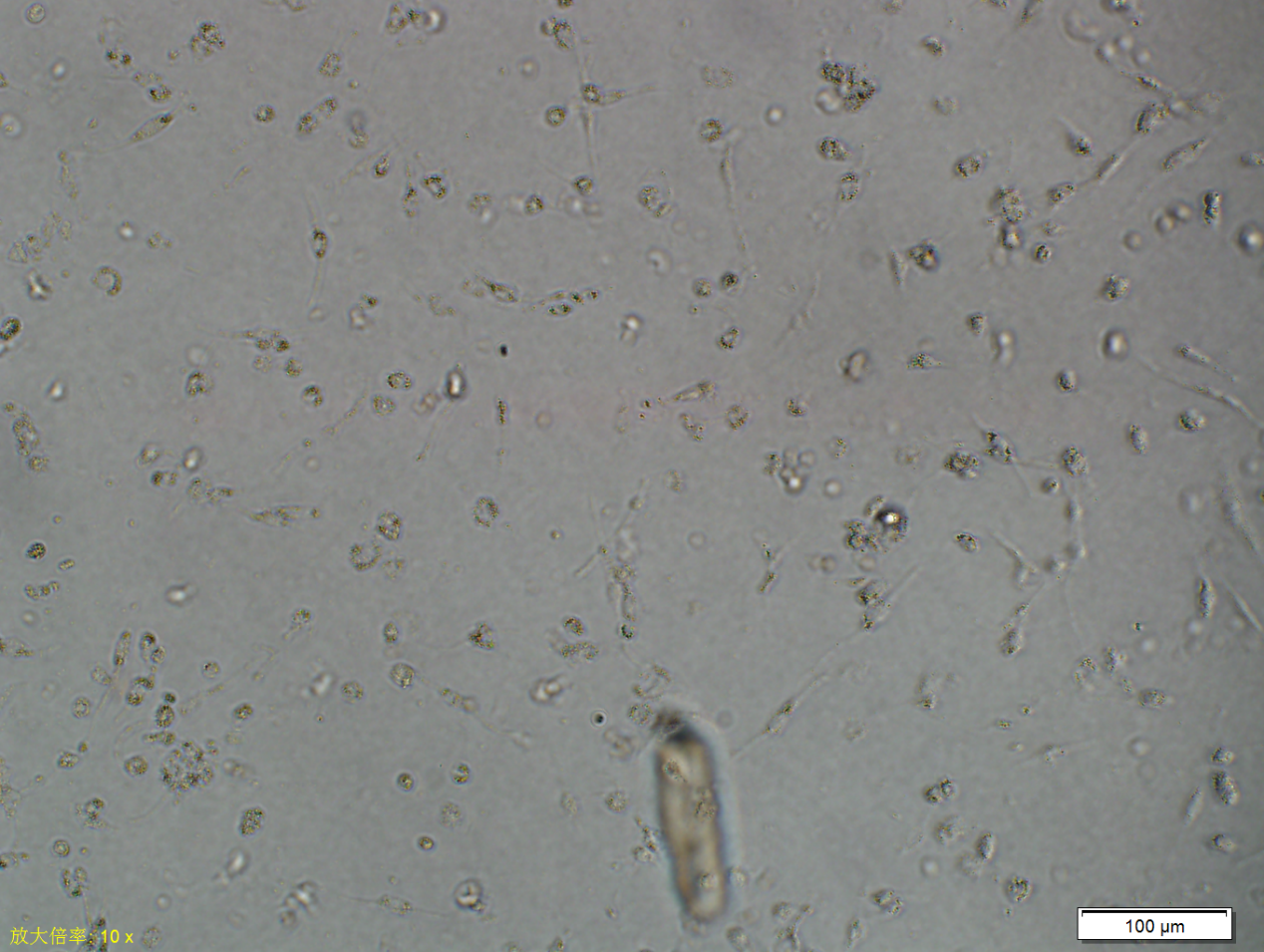


M0


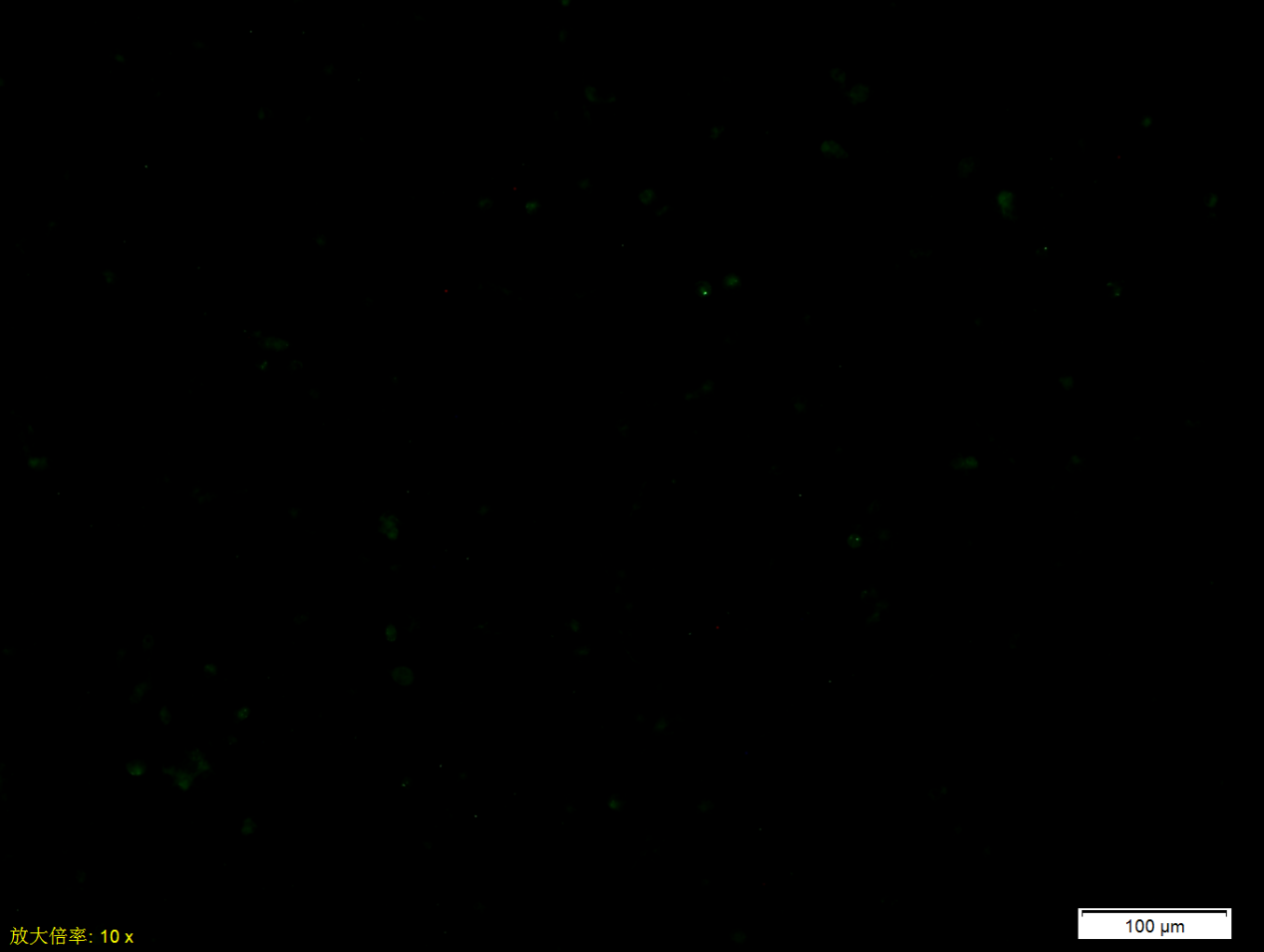


M0 (CD-206-FITC)


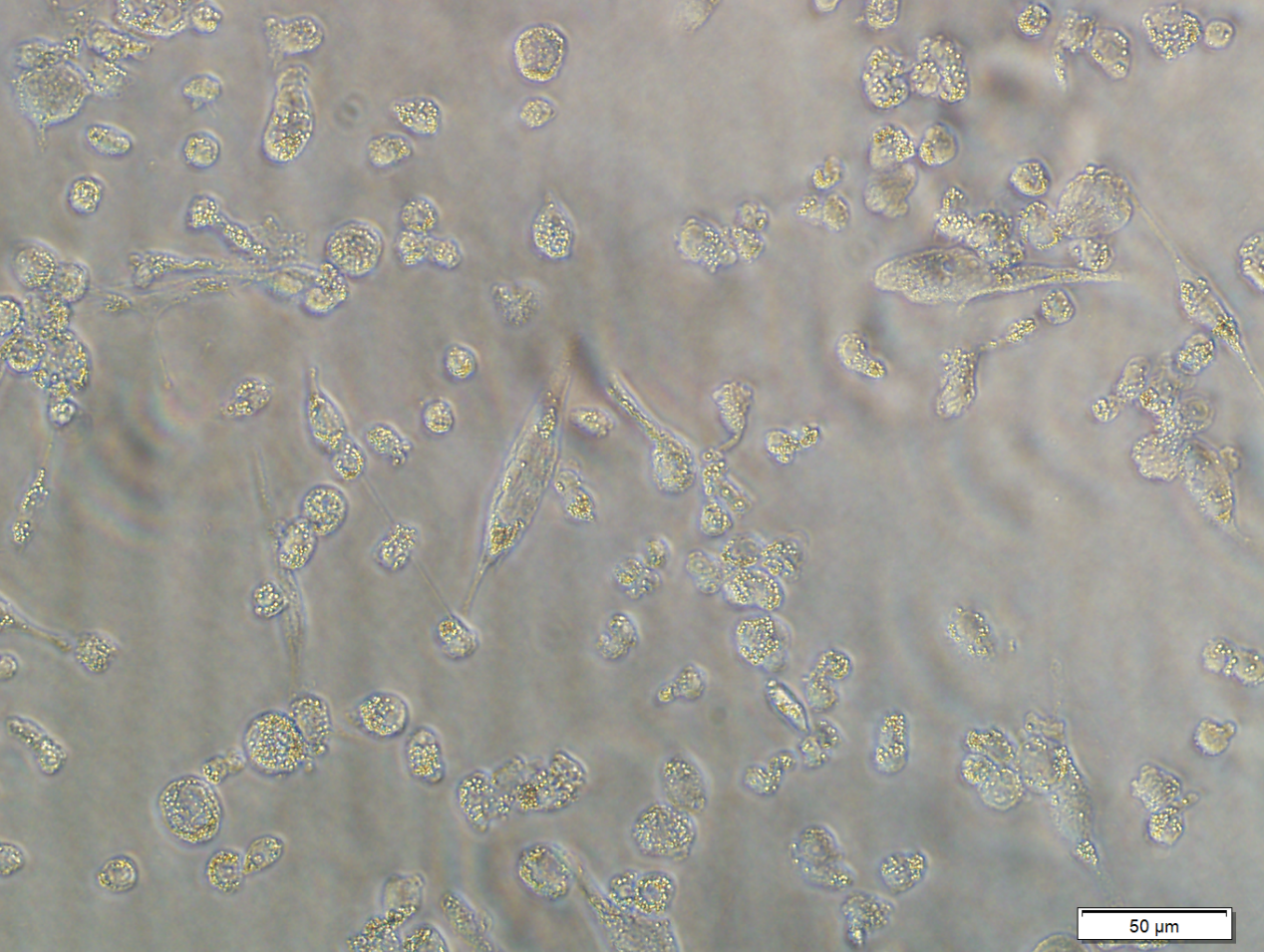


IL-4-M2


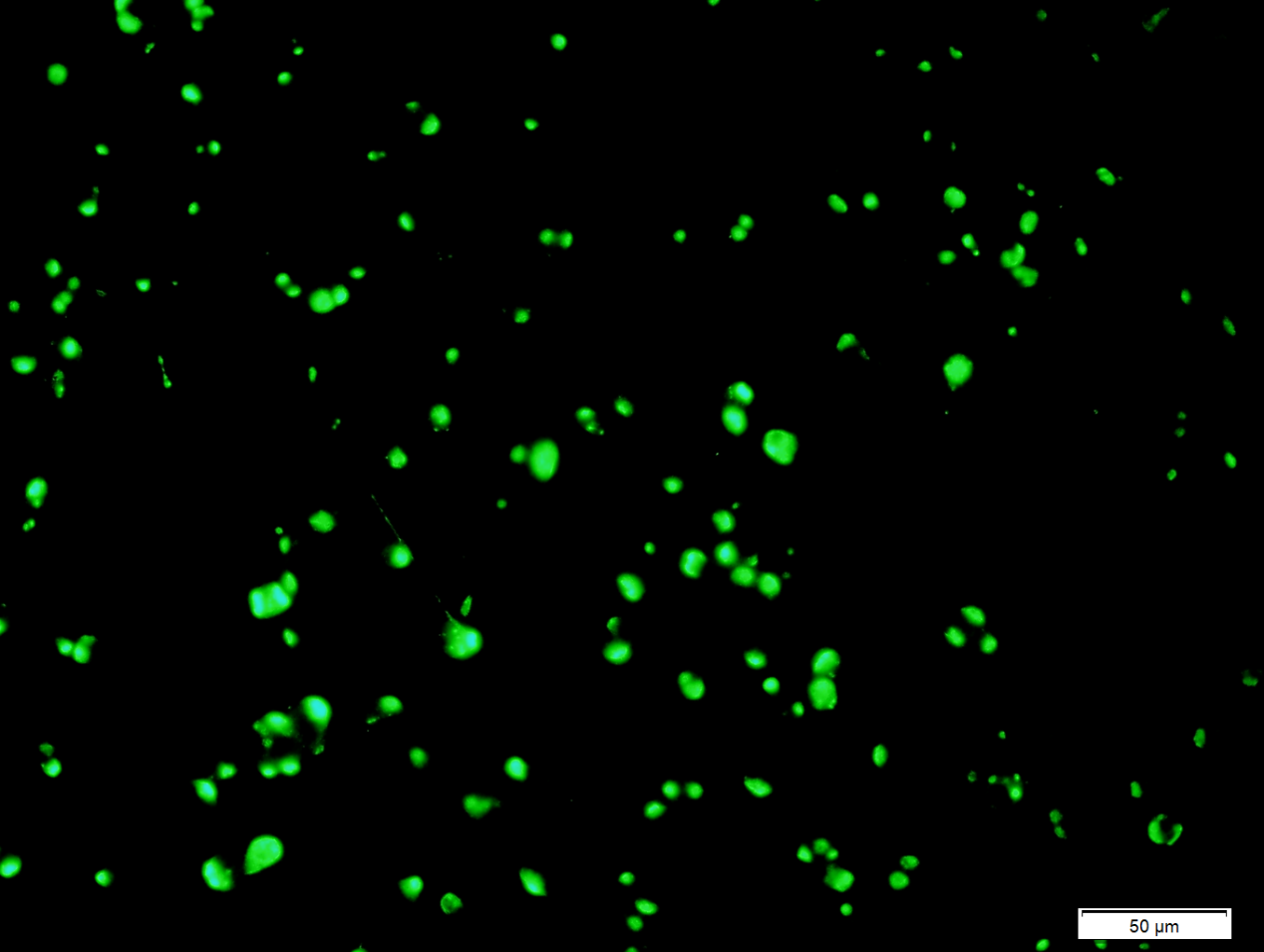


IL-4-M2 (CD206-FITC)


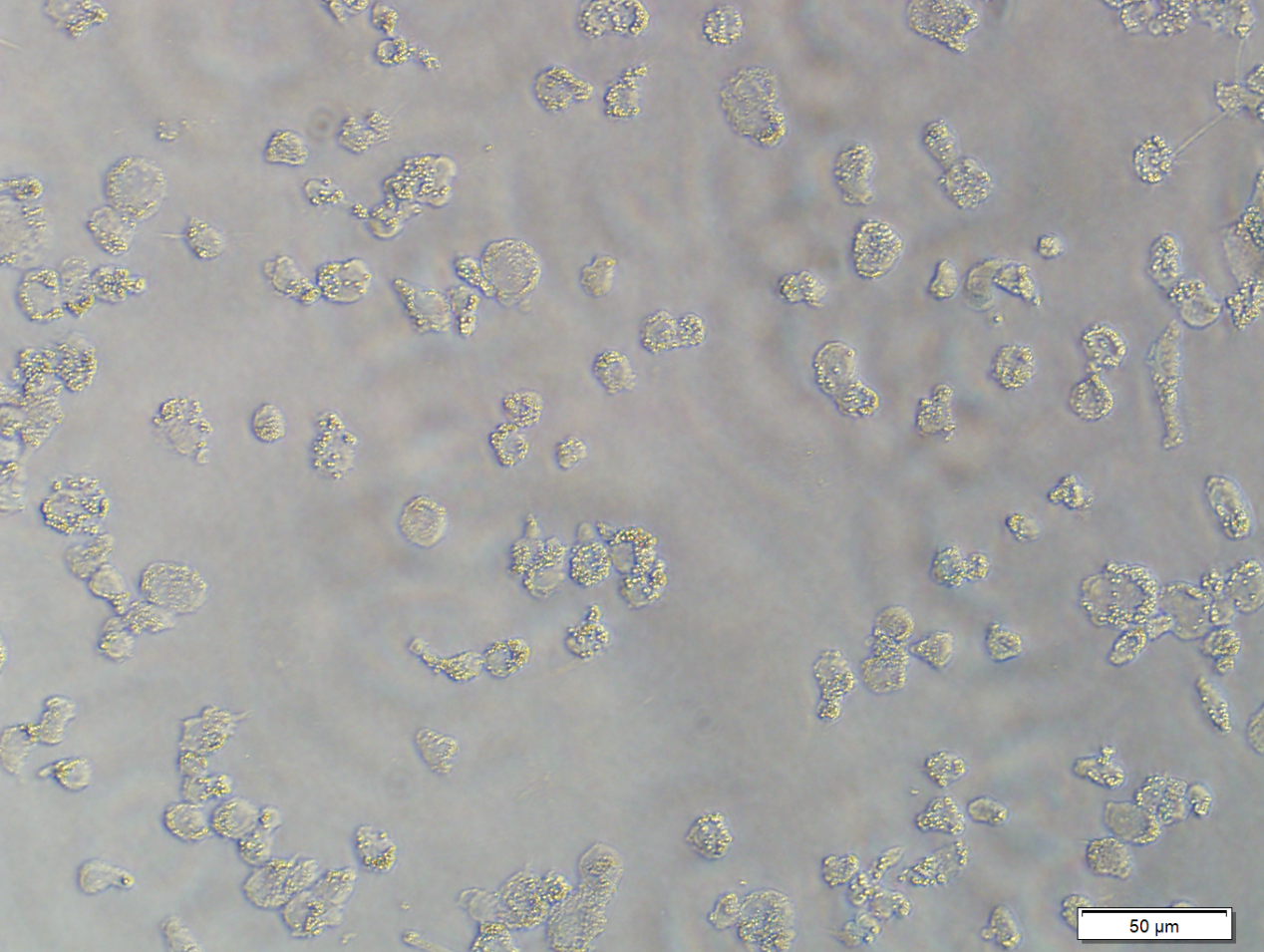


2B4-exo-Mφ


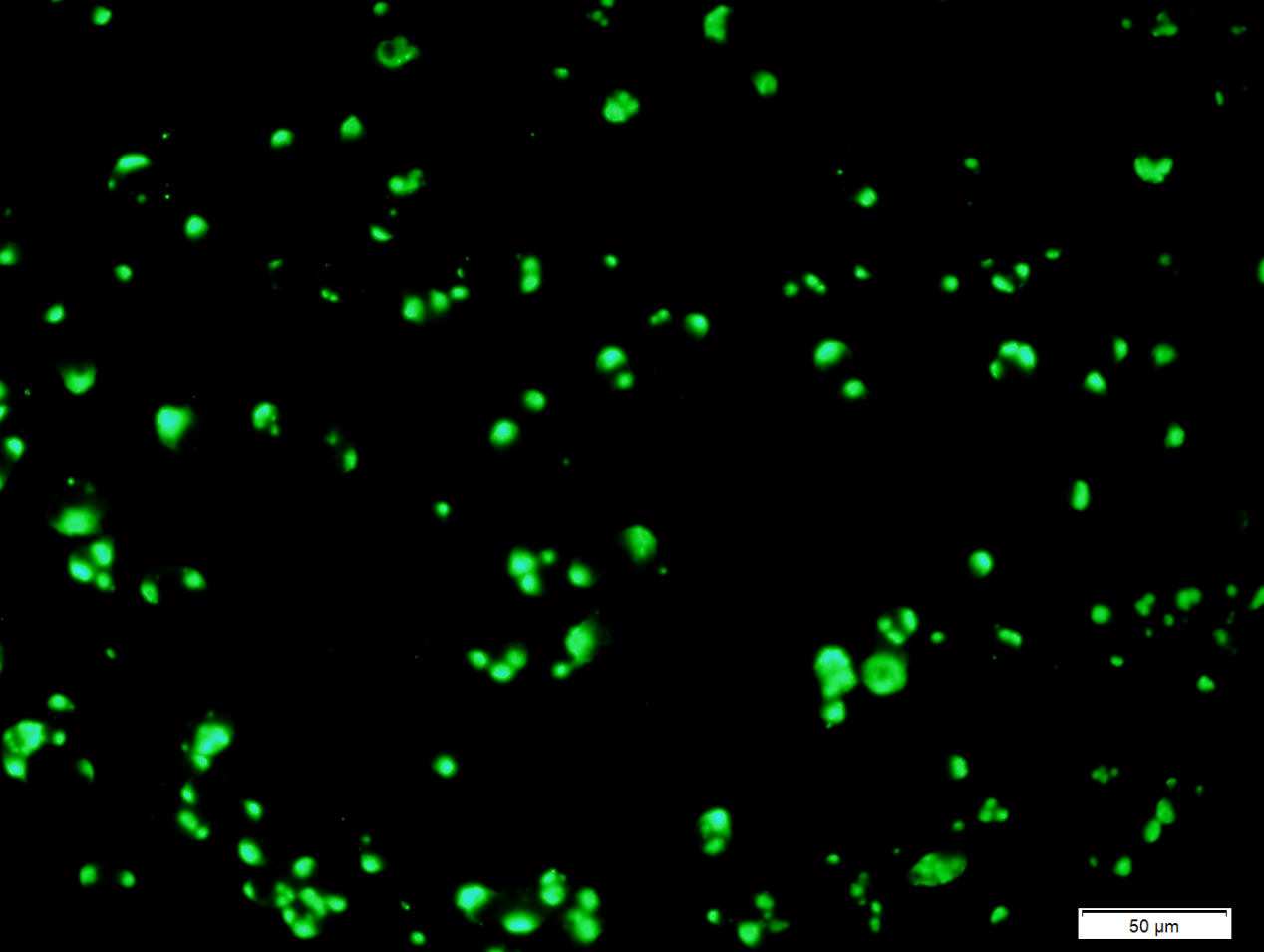


2B4-exo-Mφ (CD-206-FITC)


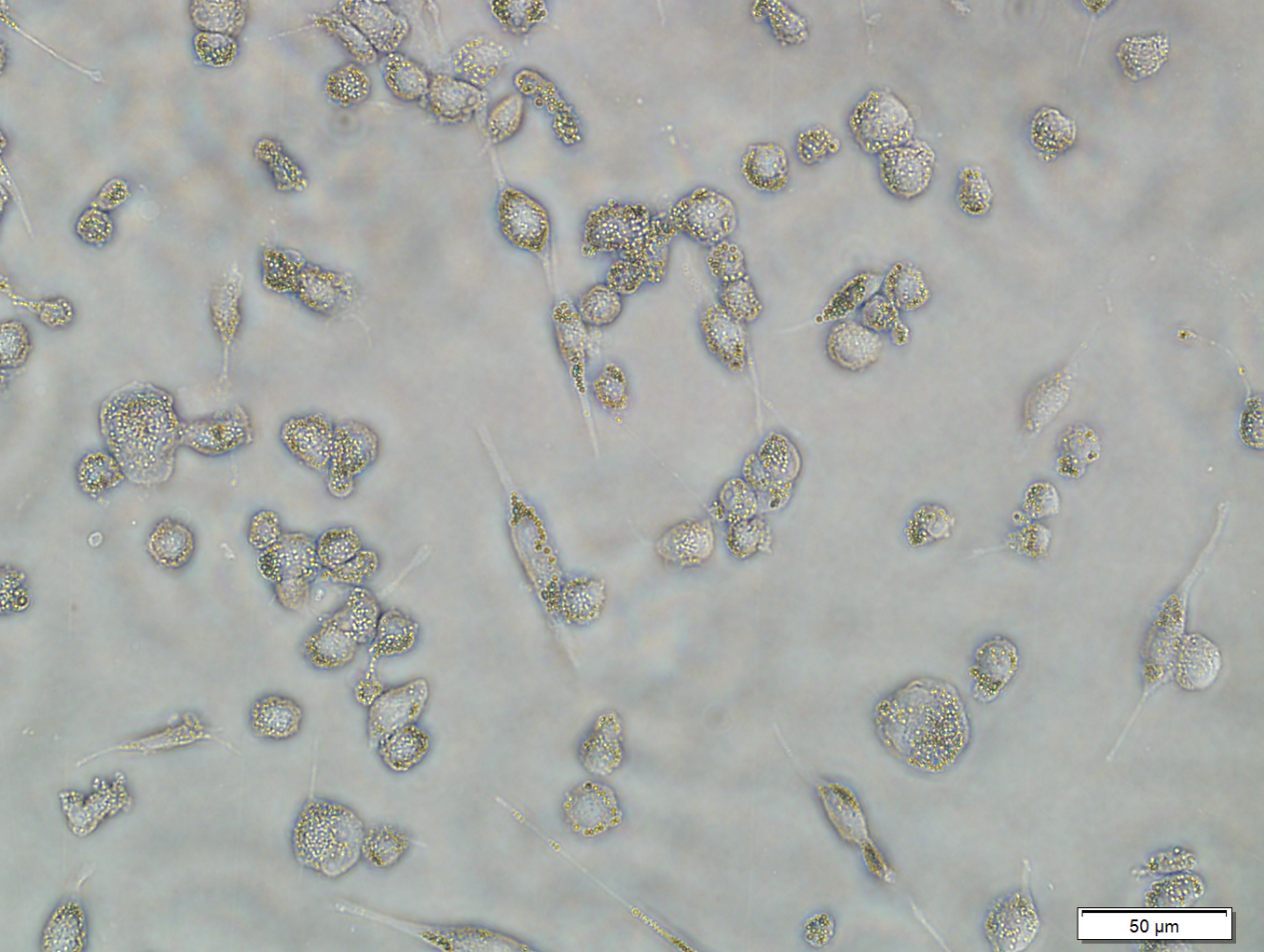


2B4-GW4869-Mφ


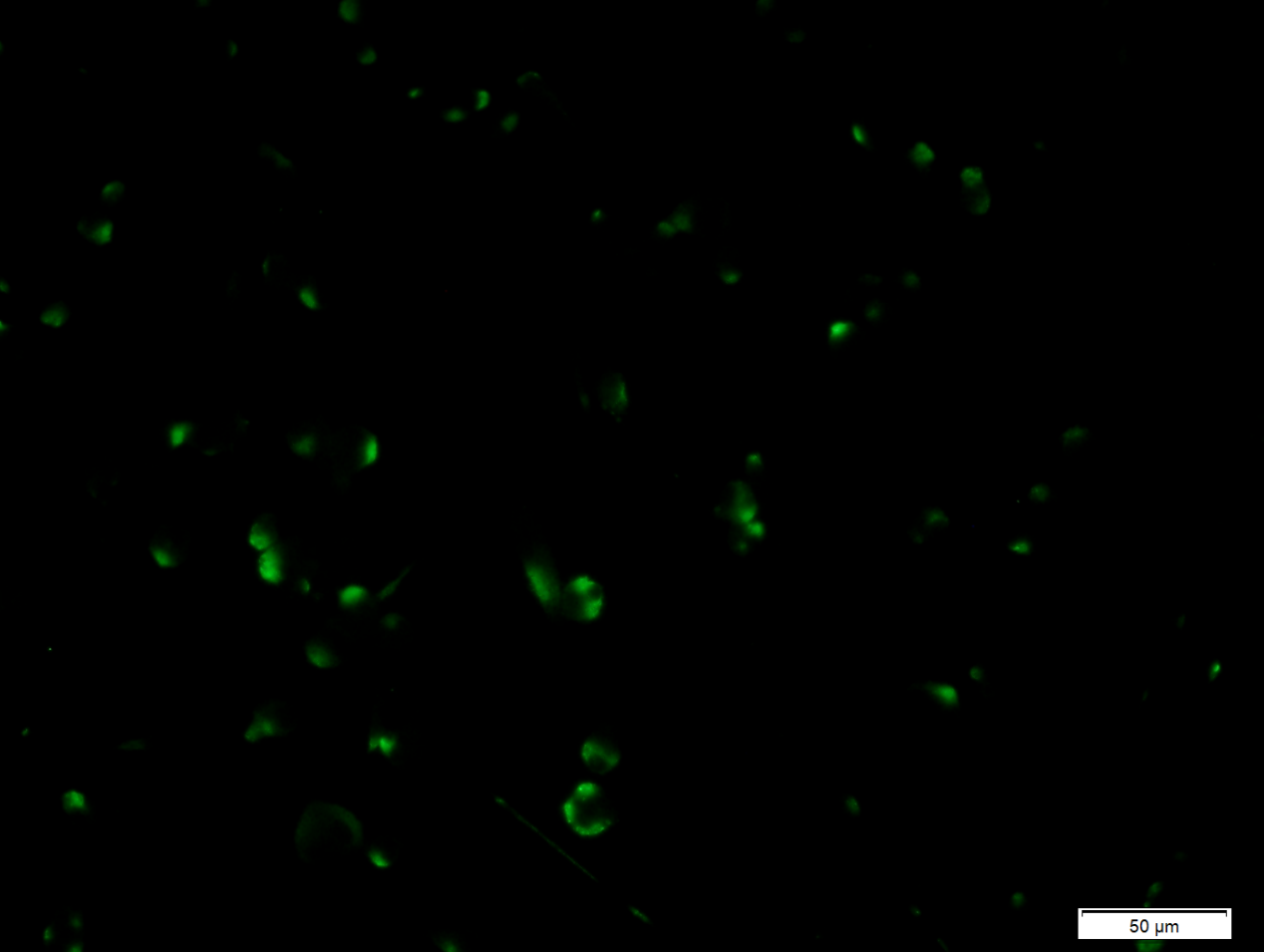


2B4-GW4869-Mφ (CD-206-FITC)


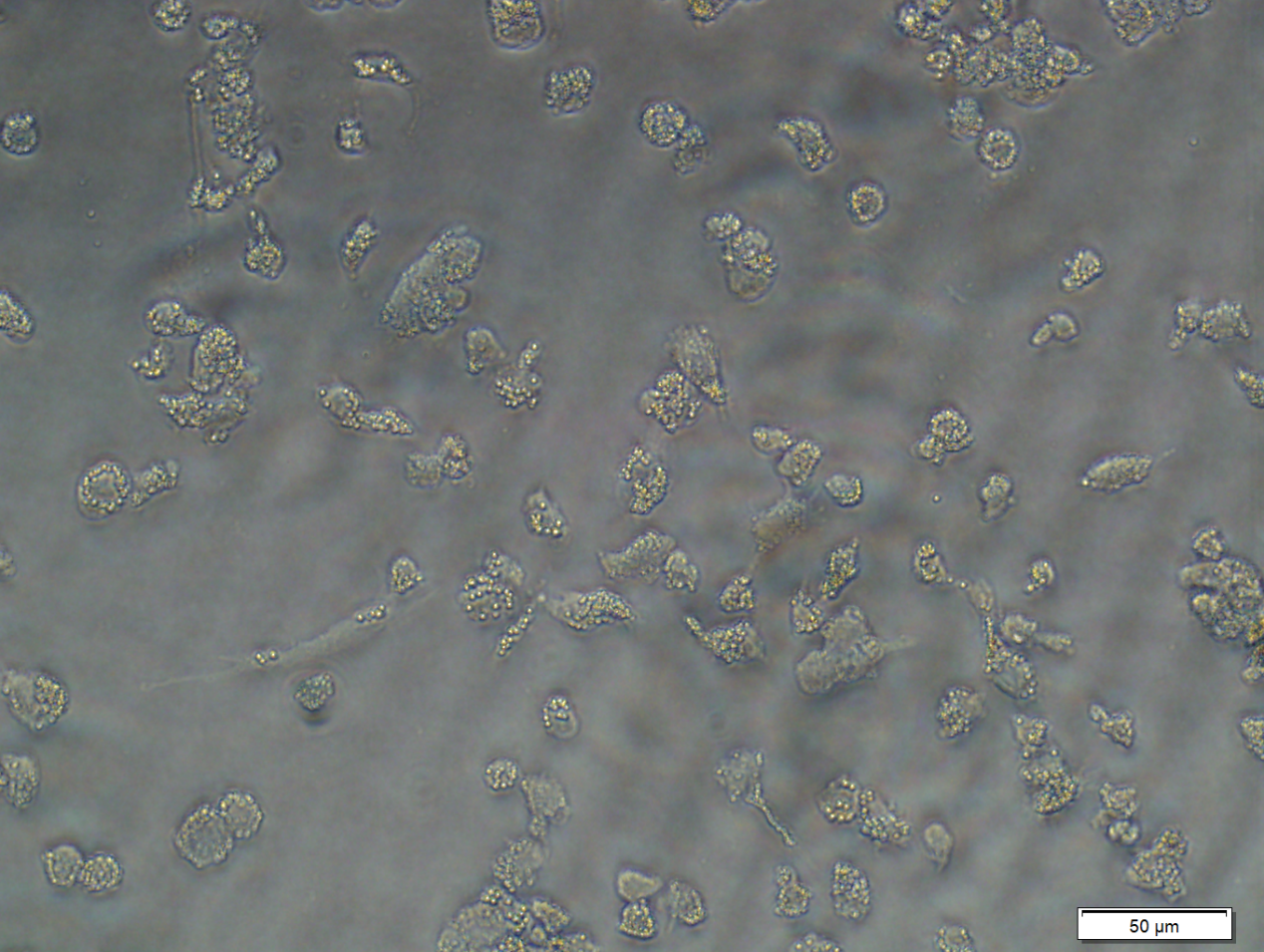


1E8-exo-Mφ


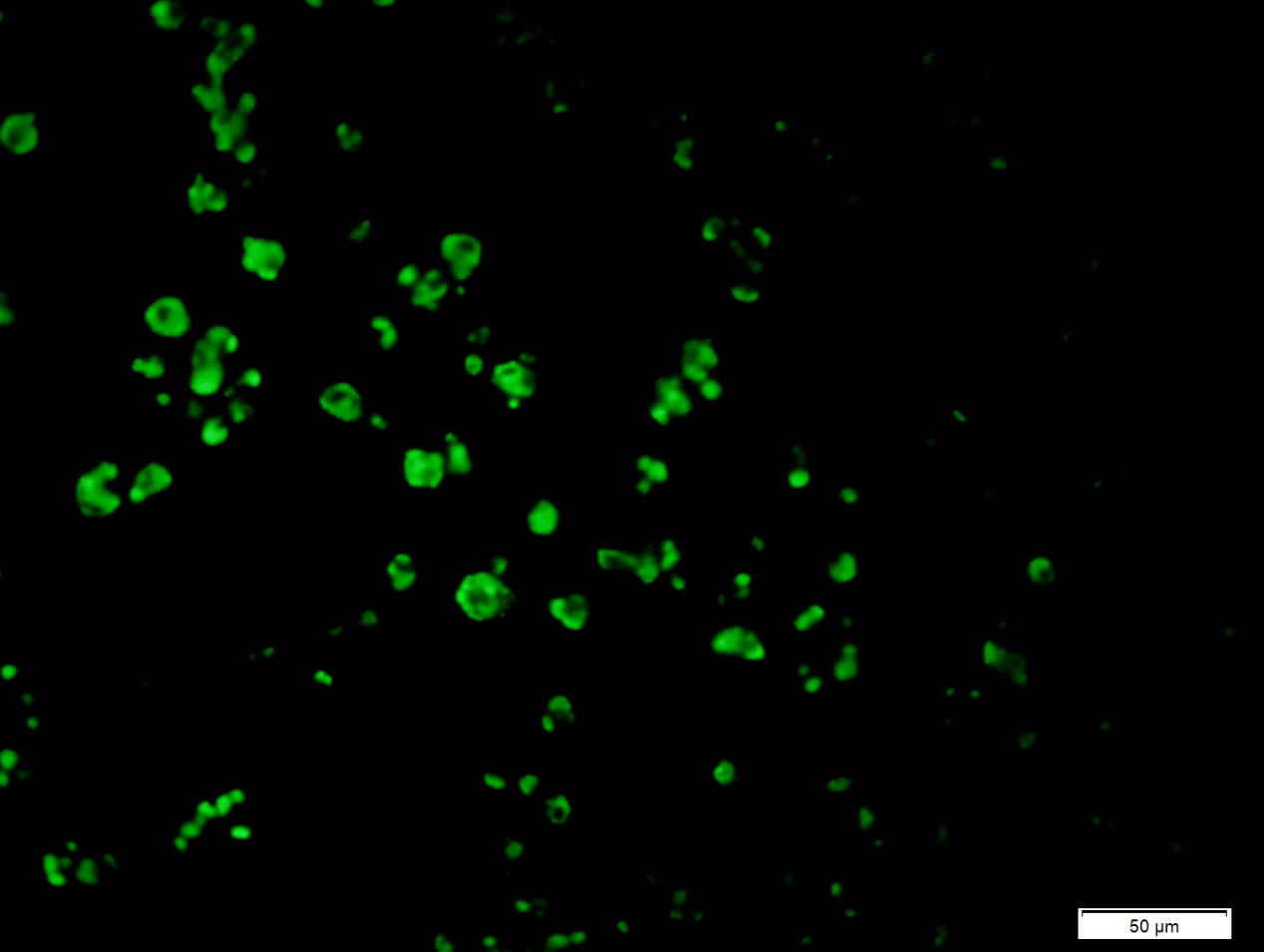


1E8-exo-Mφ (CD206-FITC)


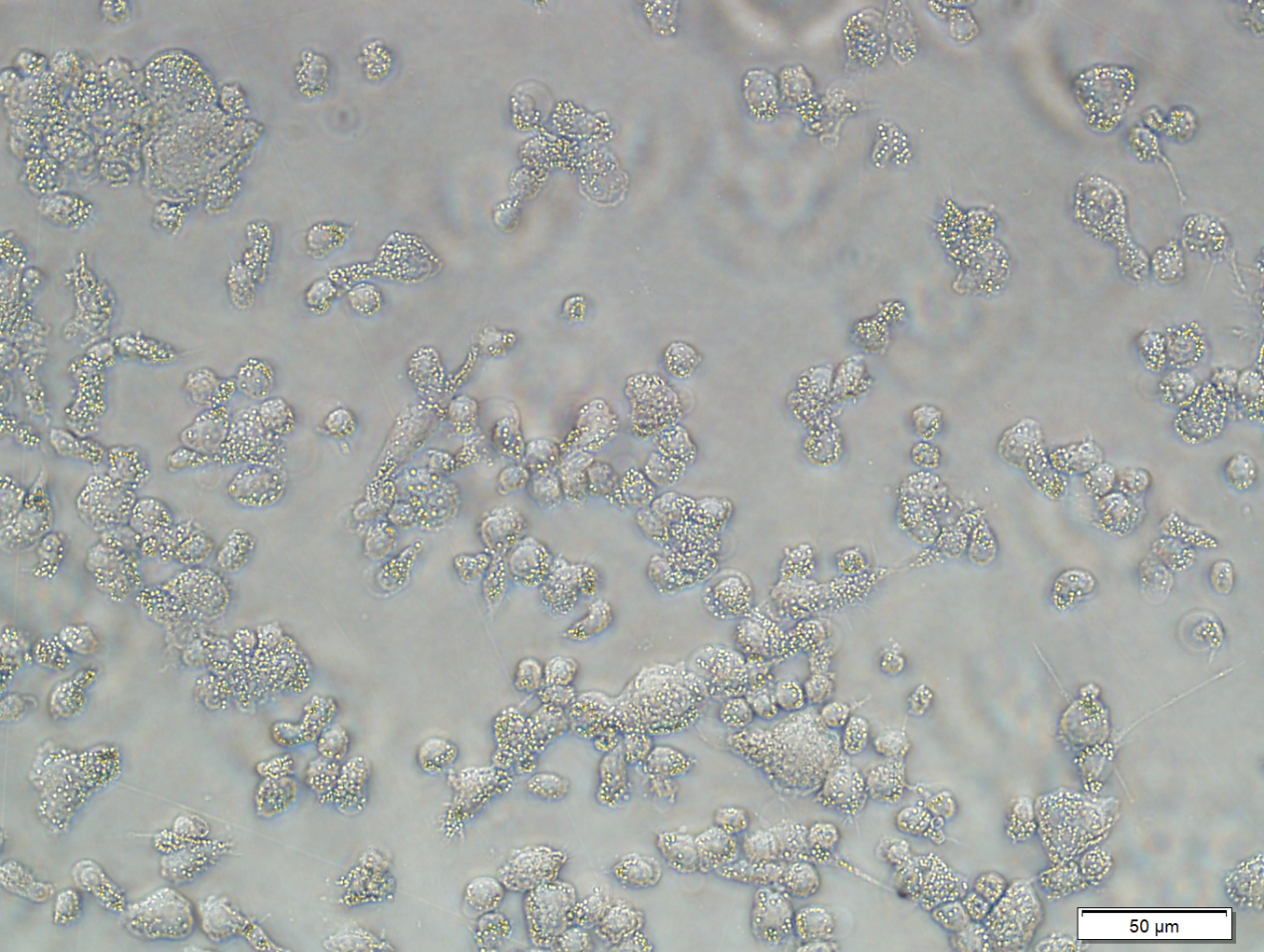


1E8-GW4869-Mφ


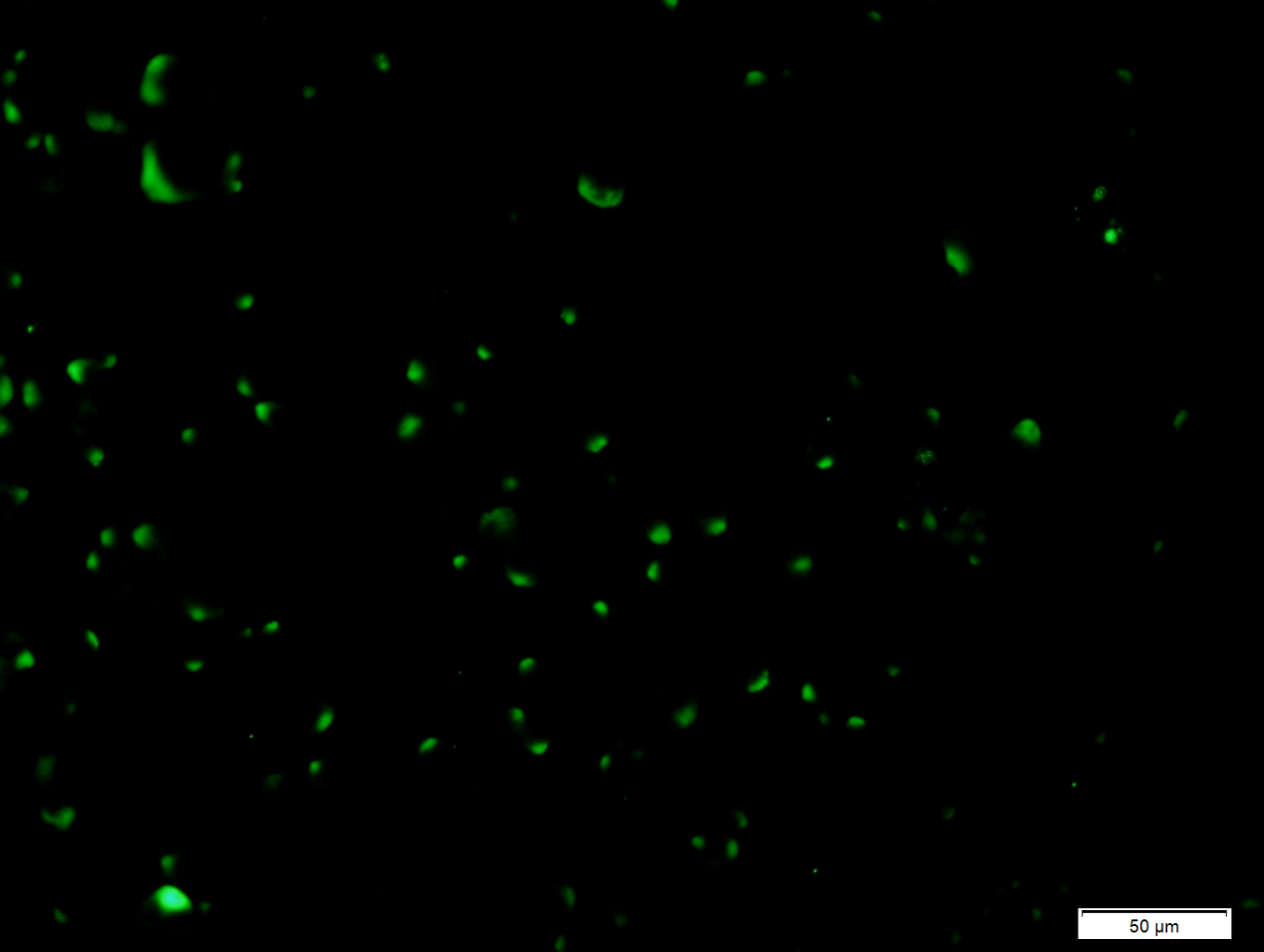


1E8-GW4869-Mφ (CD206-FITC)

**Figure 4.** **Blocking the release of PCa with GW4869 affected the tumor-promoting ability of tumor-associated macrophages.**

A. Macrophages induced with CM from PCa treated with GW4869 decreased the invasive ability of PCa cells. Macrophages were incubated with PCa-exos, CM-2B4-GW4869/CM-1E8-GW4869, and IL-4 for 48 h. CM from each group was collected and added to PCa cells in transwell chambers. Cells in these transwell chambers were stained and observed from three randomly chosen fields (original magnification, 100×) 48 h later. * P < 0.05; ** P < 0.01.


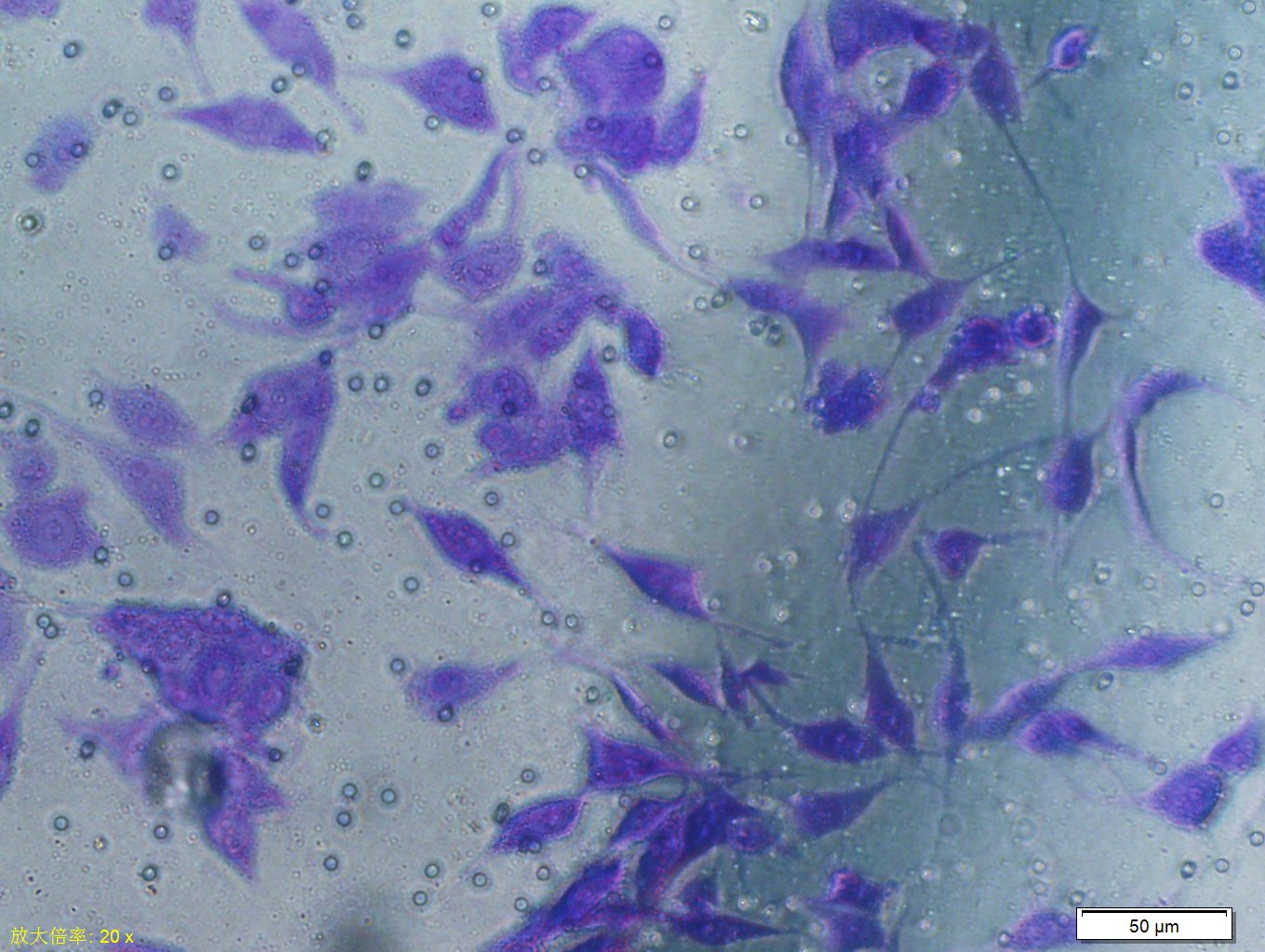


PC-3M-2B4 treated with M0-CM


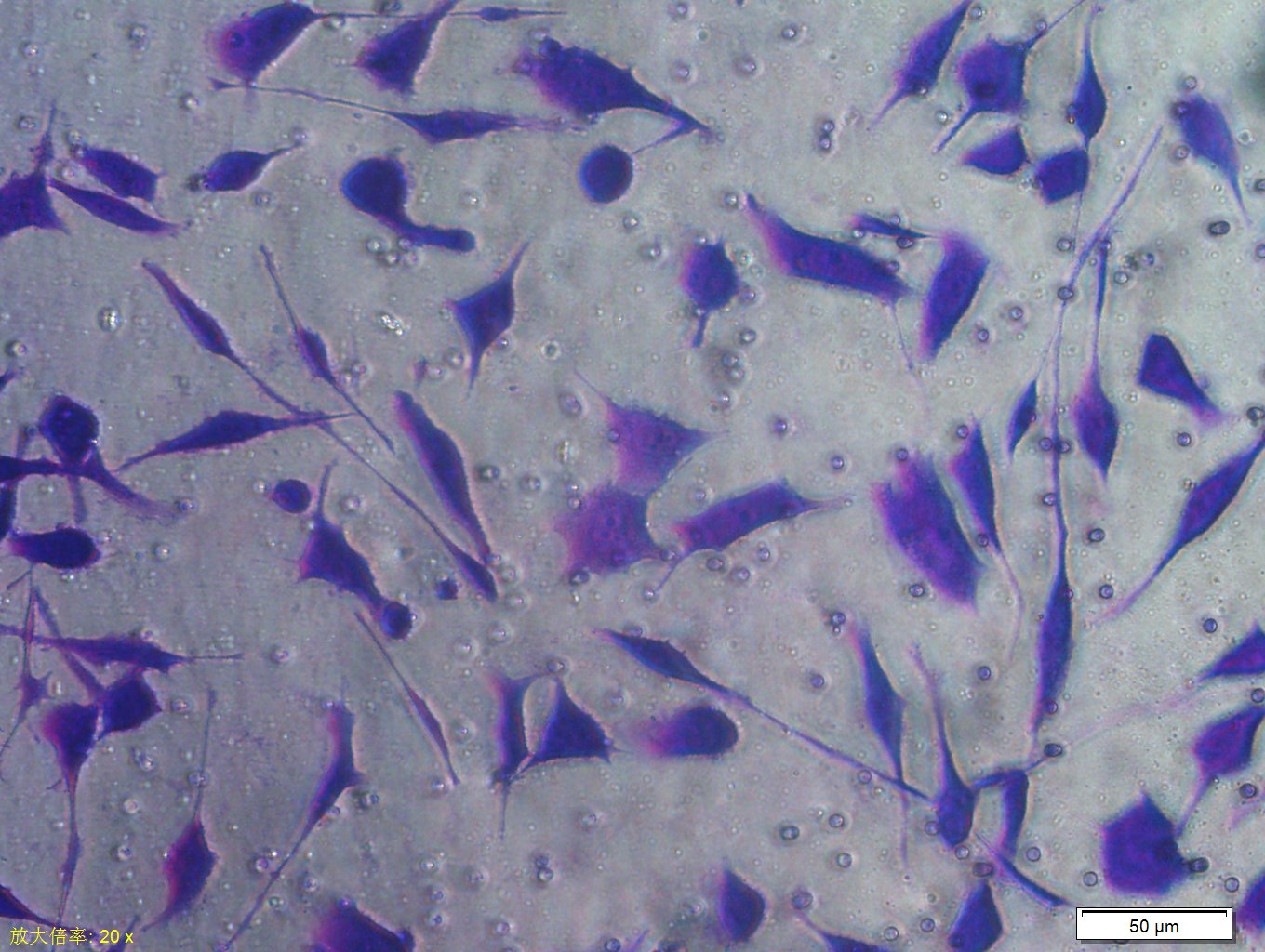


PC-3M-1E8 treated with M0-CM


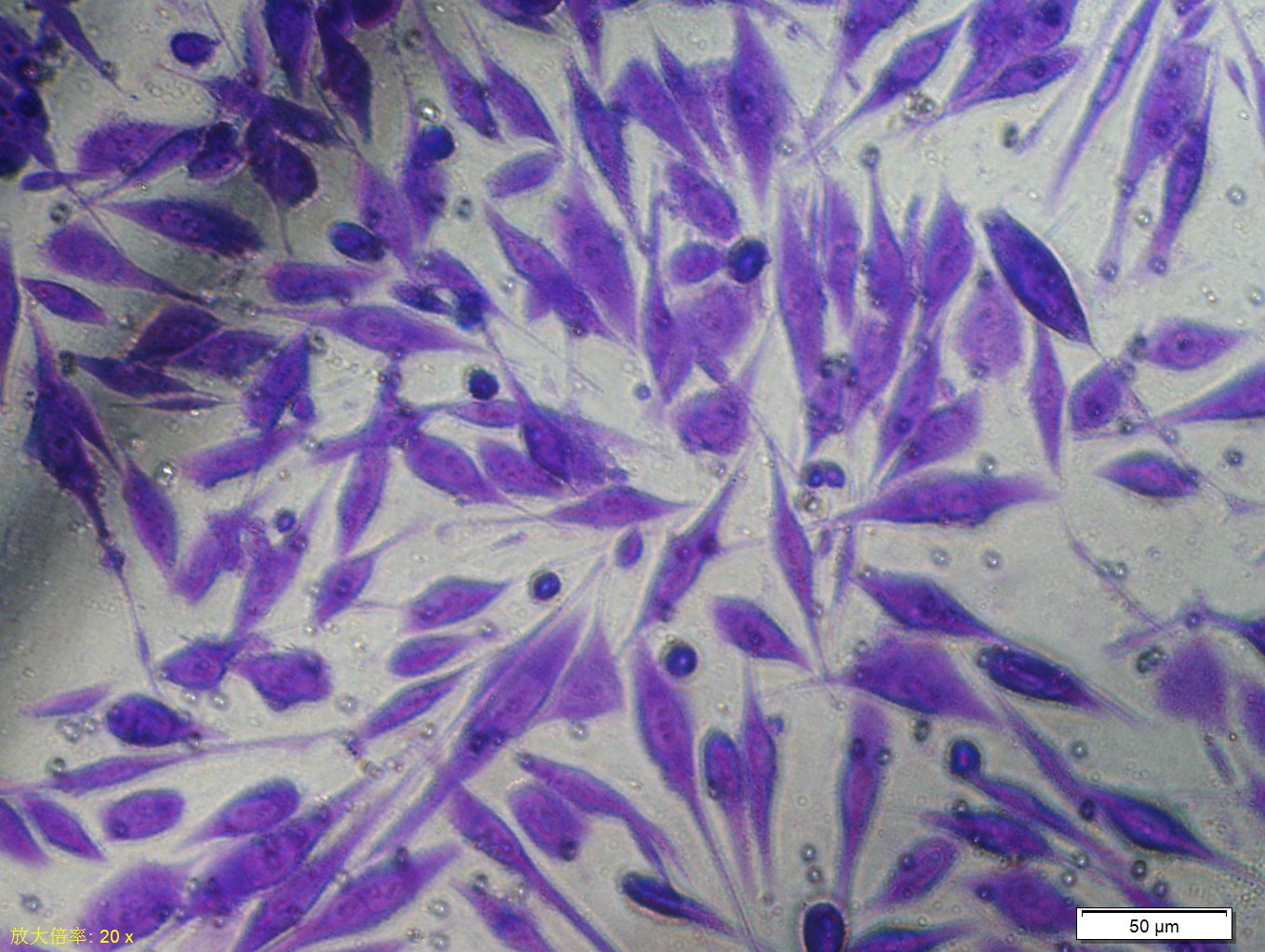


PC-3M-2B4 treated with IL-4-M2-CM


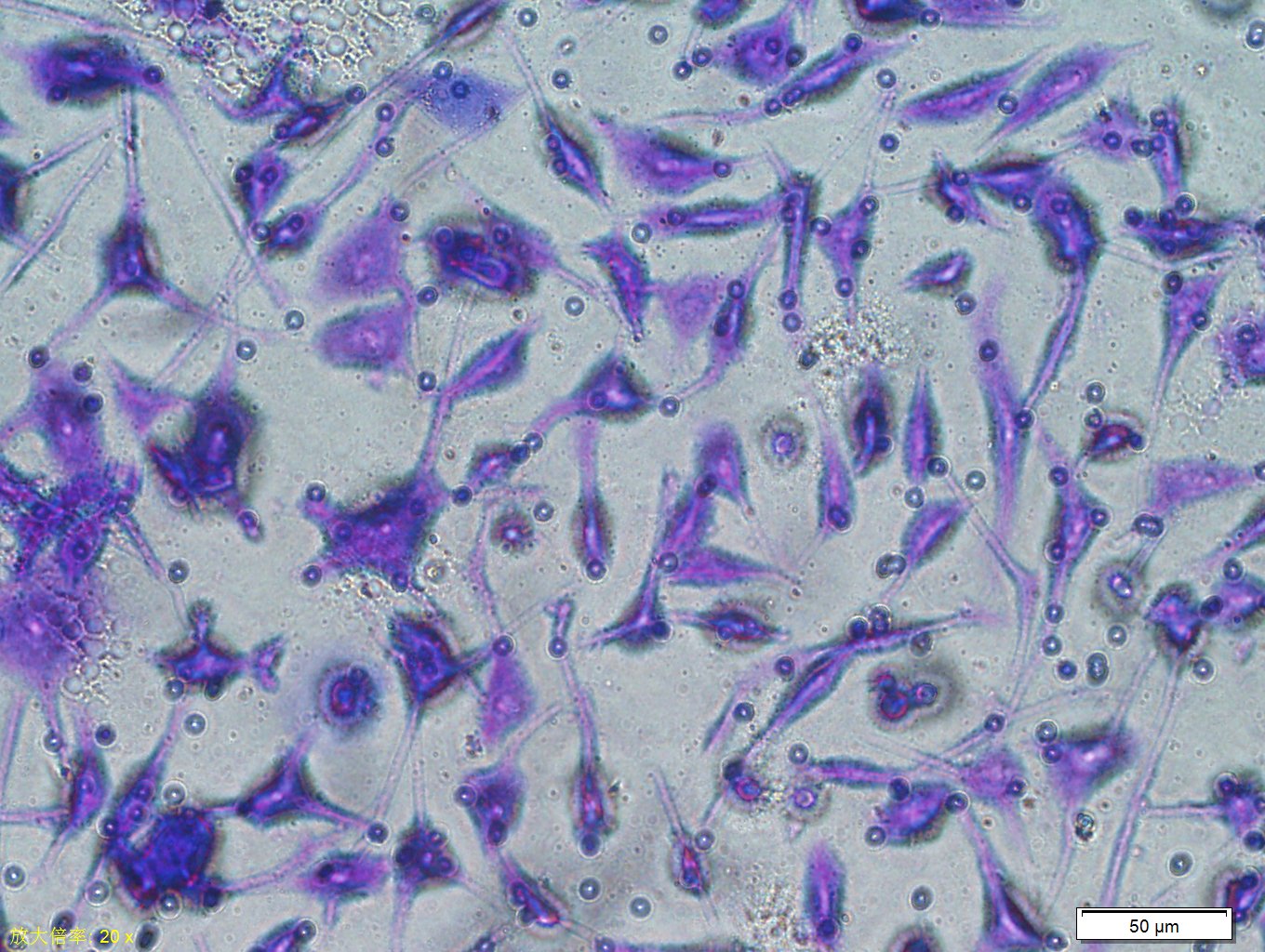


PC-3M-1E8 treated with IL-4-M2-CM


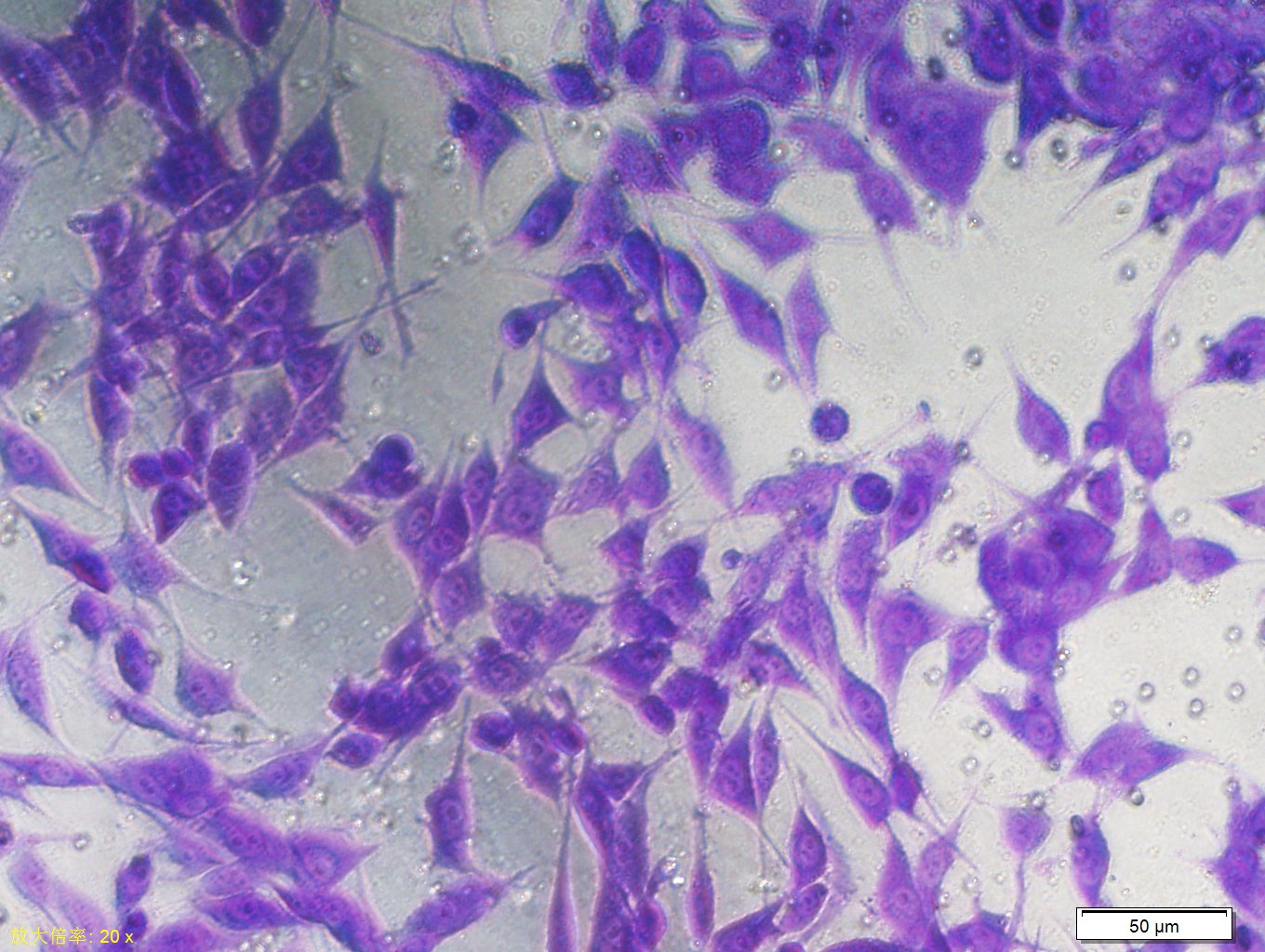


PC-3M-2B4 treated with 2B4-exo-Mø-CM


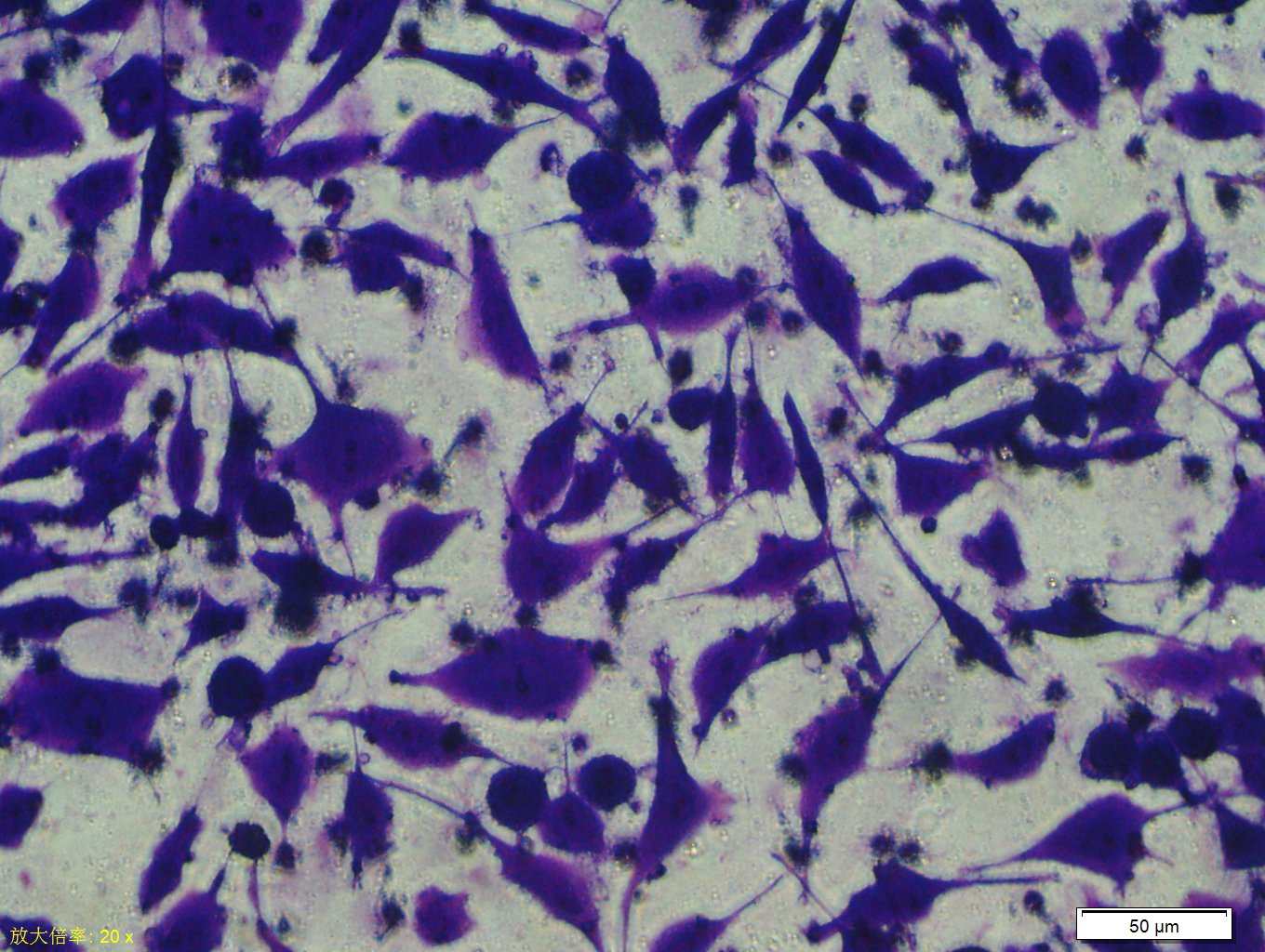


PC-3M-1E8 treated with 2B4-exo-Mø-CM


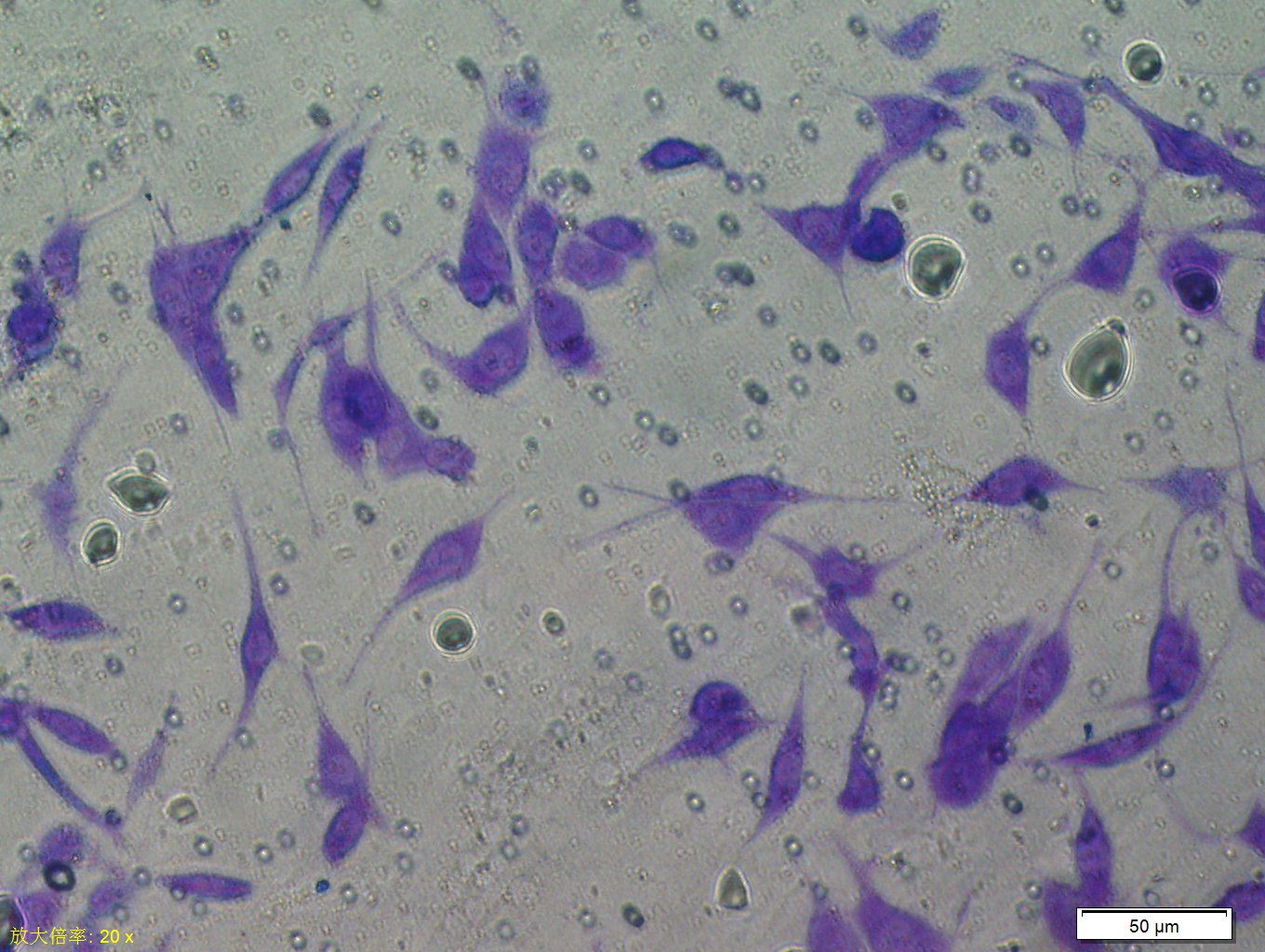


PC-3M-2B4 treated with CM-2B4-GW4869-Mø-CM


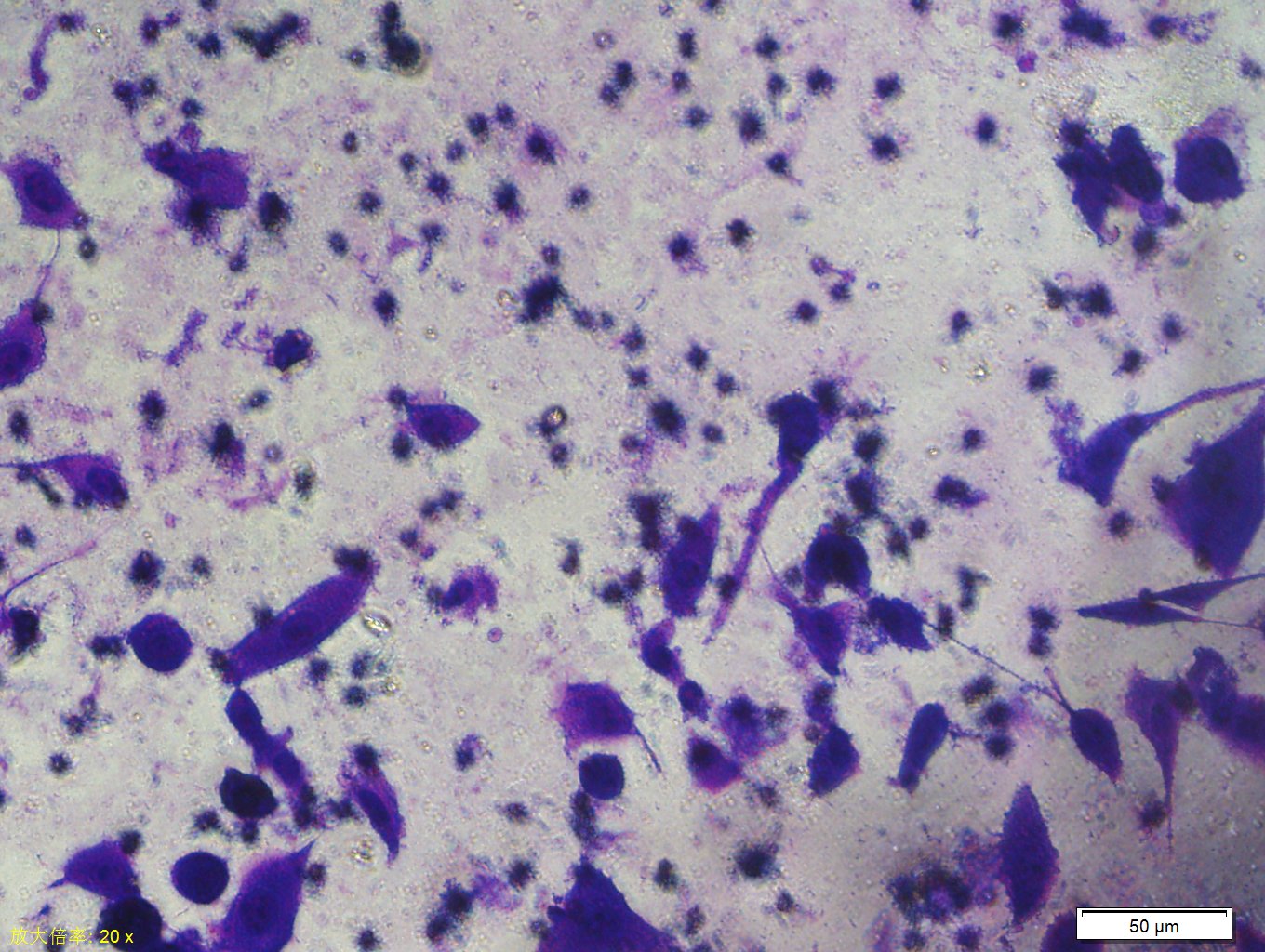


PC-3M-1E8 treated with CM-2B4-GW4869-Mø-CM


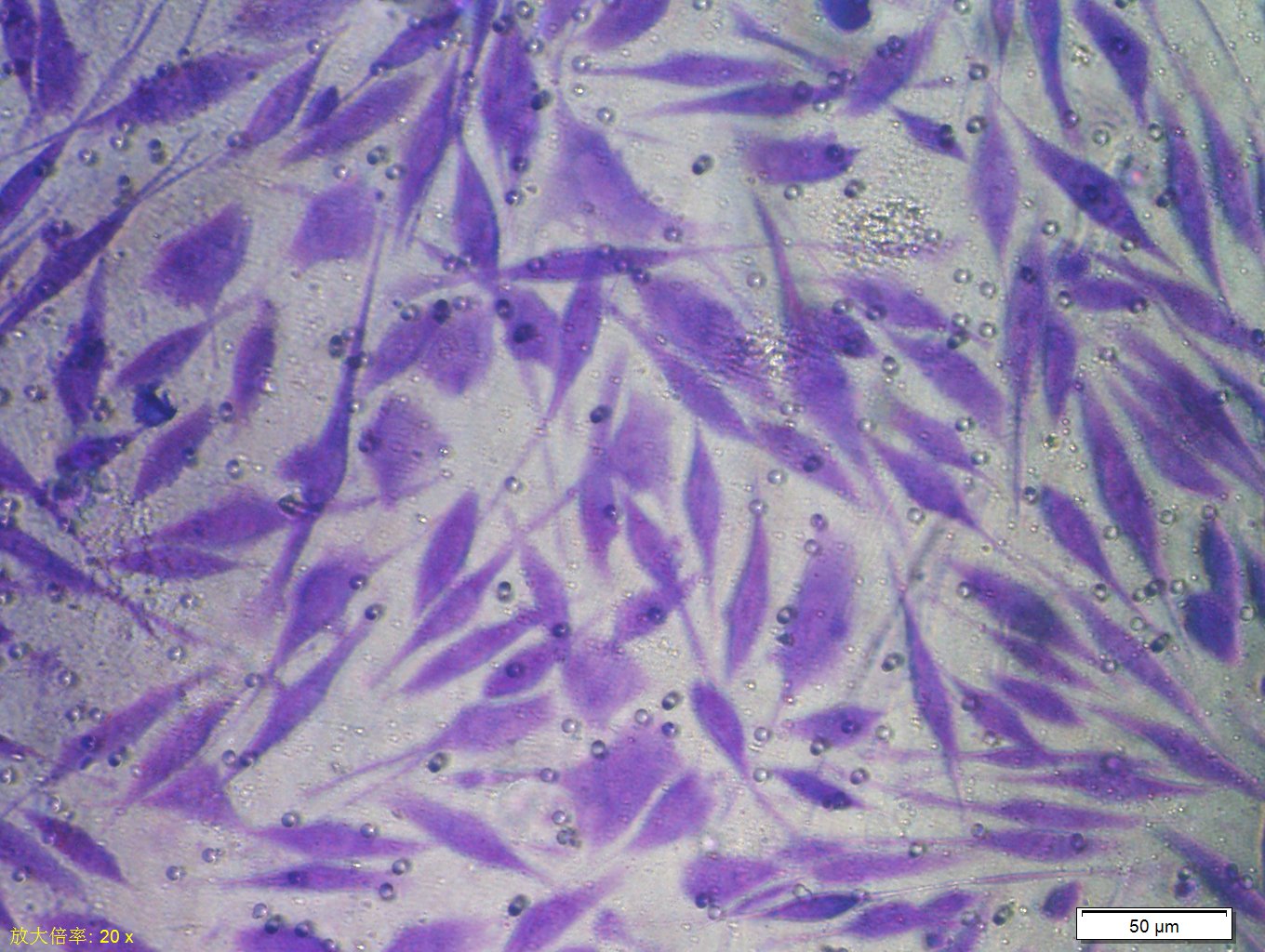


PC-3M-2B4 treated with 1E8-exo-Mø-CM


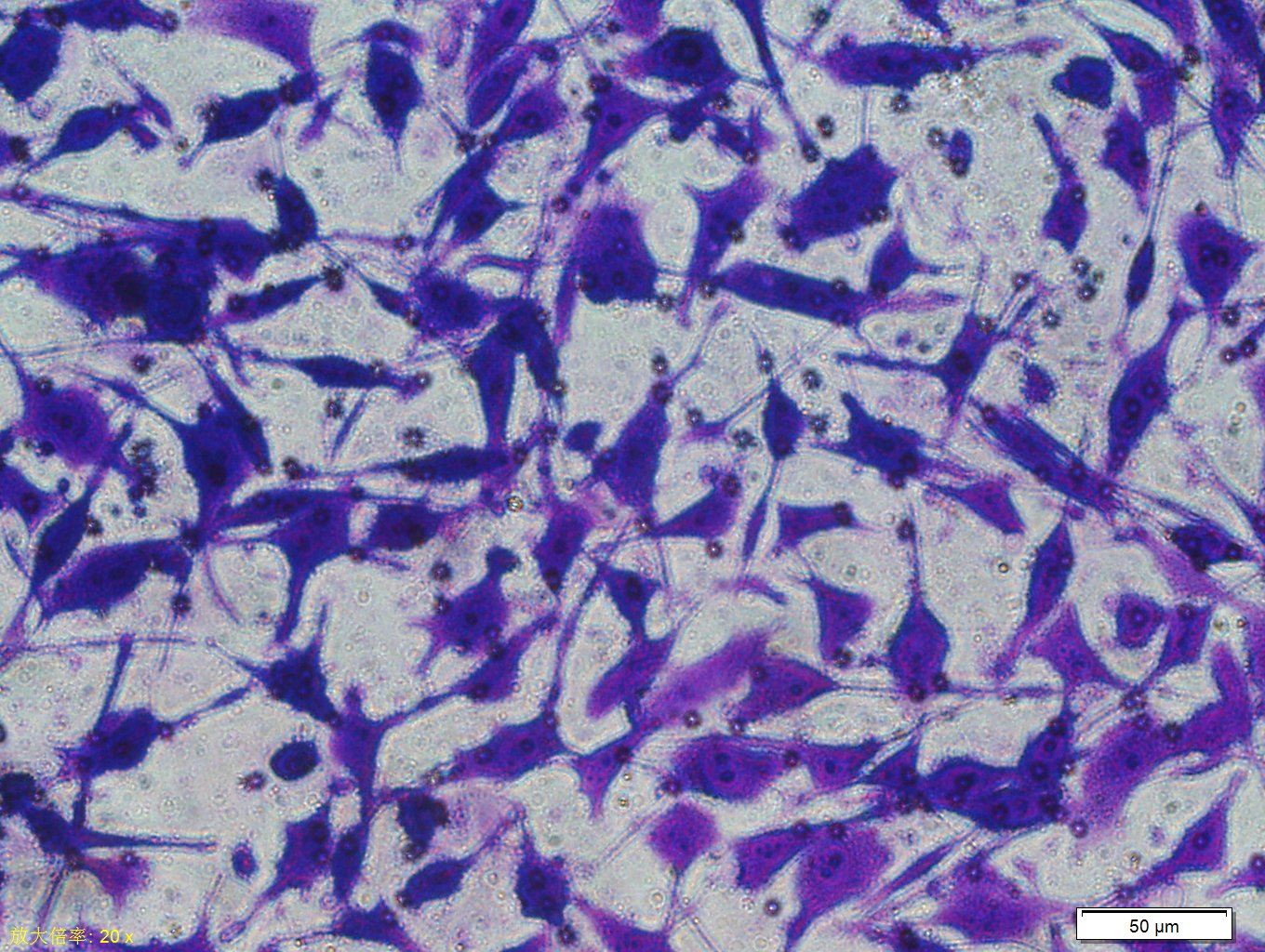


PC-3M-1E8 treated with 1E8-exo-Mø-CM


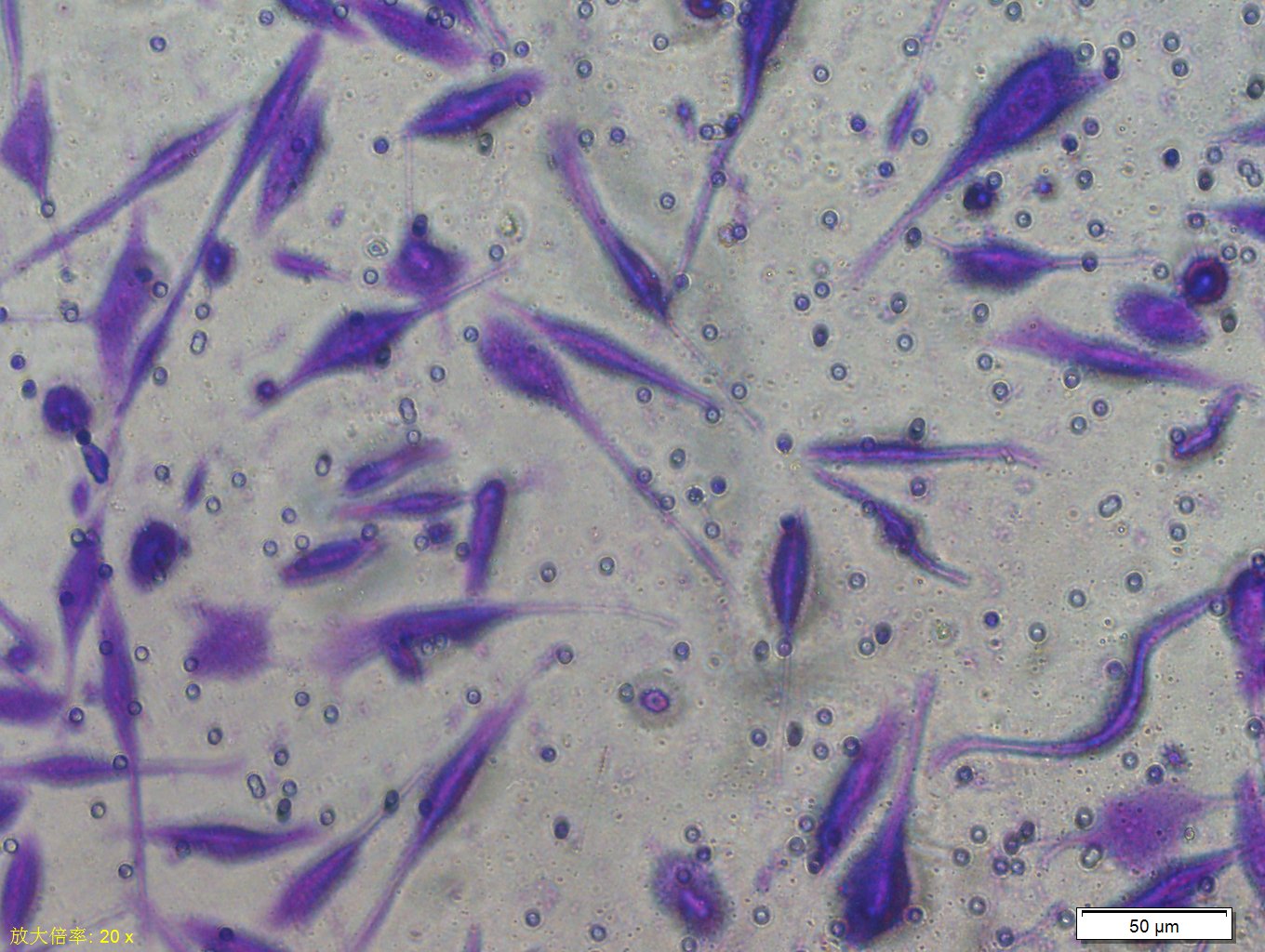


PC-3M-2B4 treated with CM-1E8-GW4869-Mø-CM


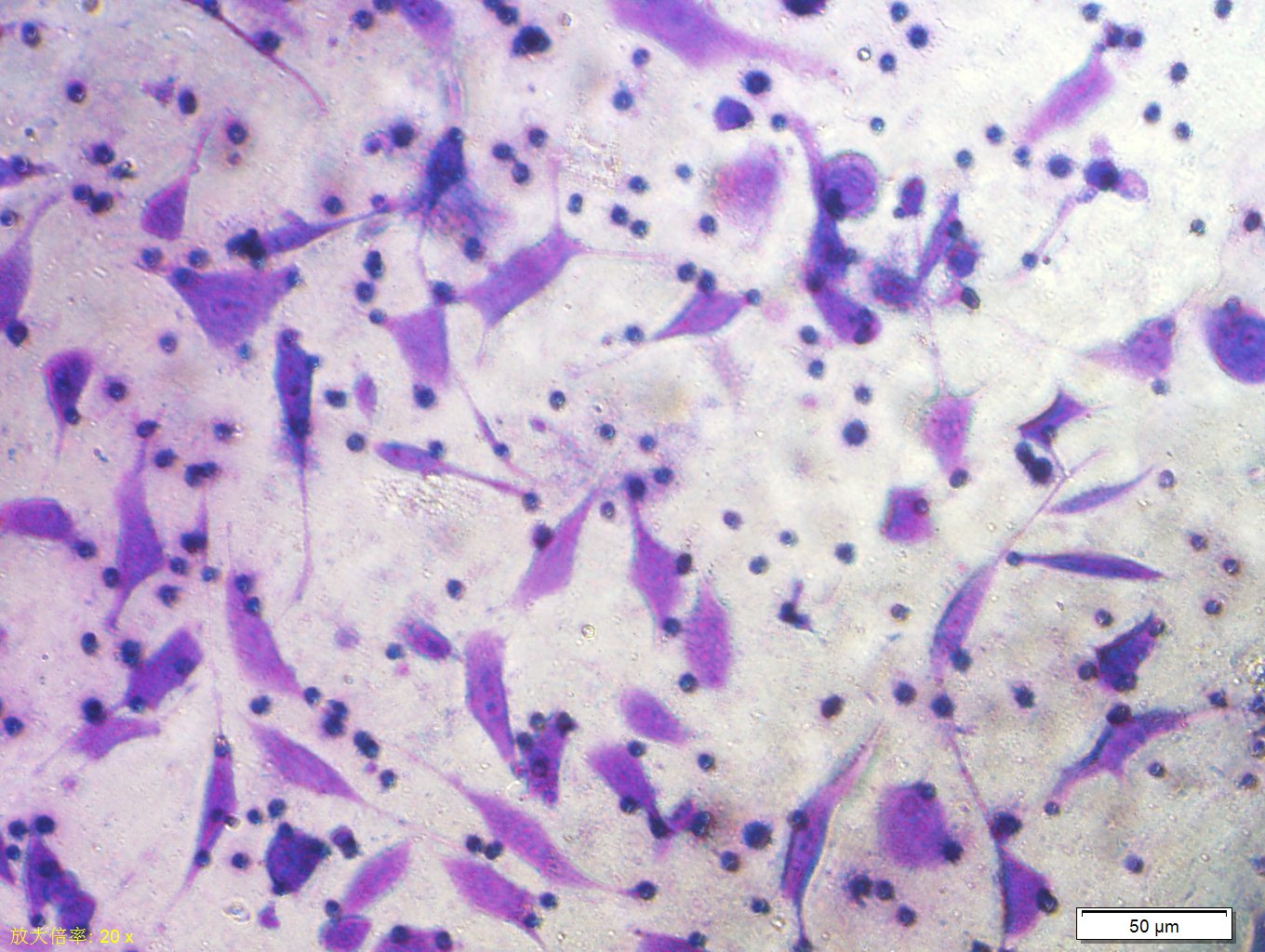


PC-3M-1E8 treated with CM-1E8-GW4869-Mø-CM

B. Macrophages induced with CM from PCa cells treated with GW4869 suppresses pro-angiogenic effects of PCa cells. HUVECs and PC-3M-2B4 or PC-3M-1E8 cells were inoculated into a 96 well plates pre-coated with matrigel, followed by the addition of CM from the above macrophages for 24 h. The tubular structures of endothelial cells were imaged with an inverted microscope equipped with CCD optics and a digital analysis system. Results were evaluated through counting the joint or vessel numbers in three fields per well (original magnification, 100×). *P < 0.05; ** P < 0.01.


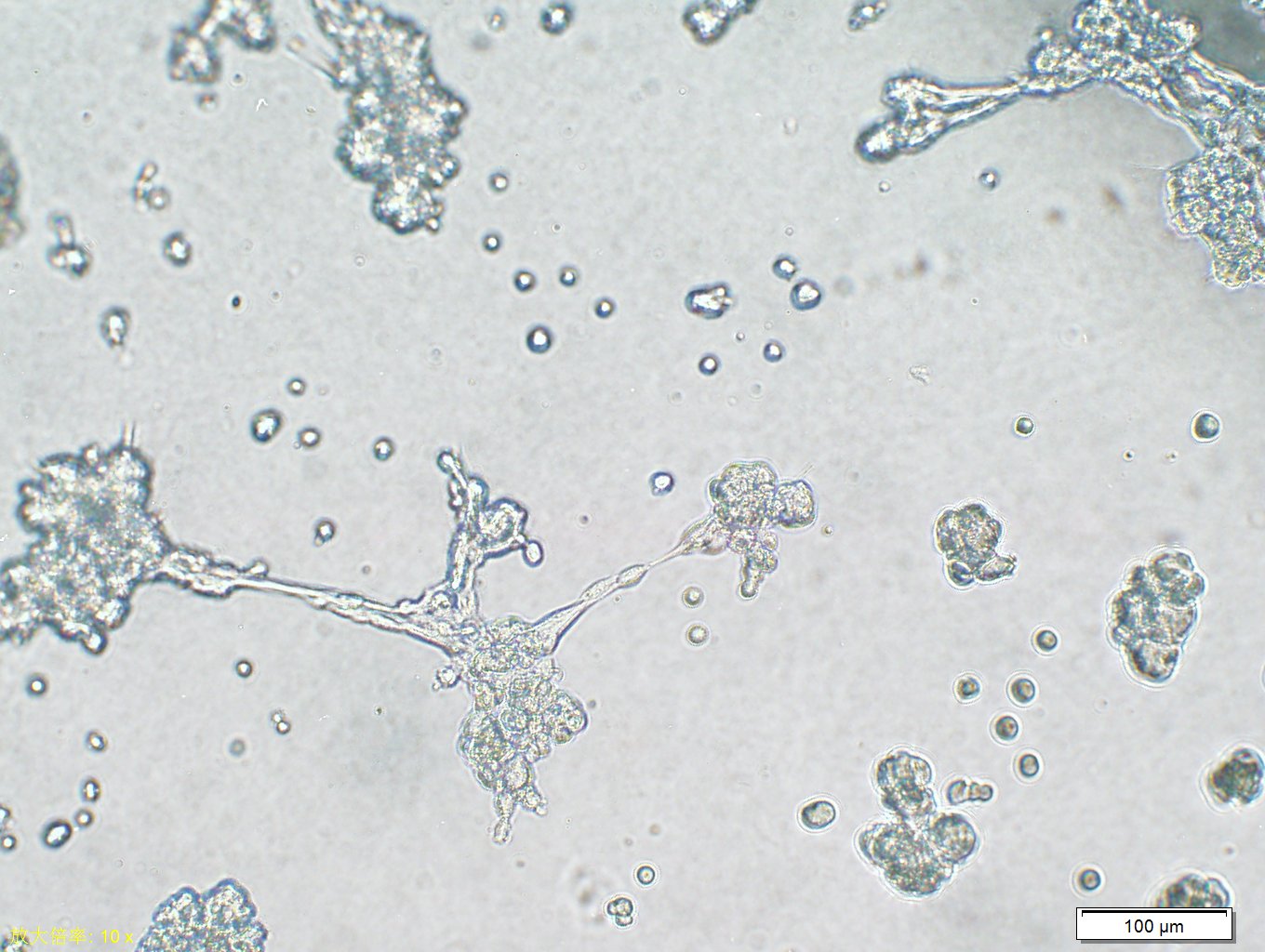


M0-CM + 2B4 group


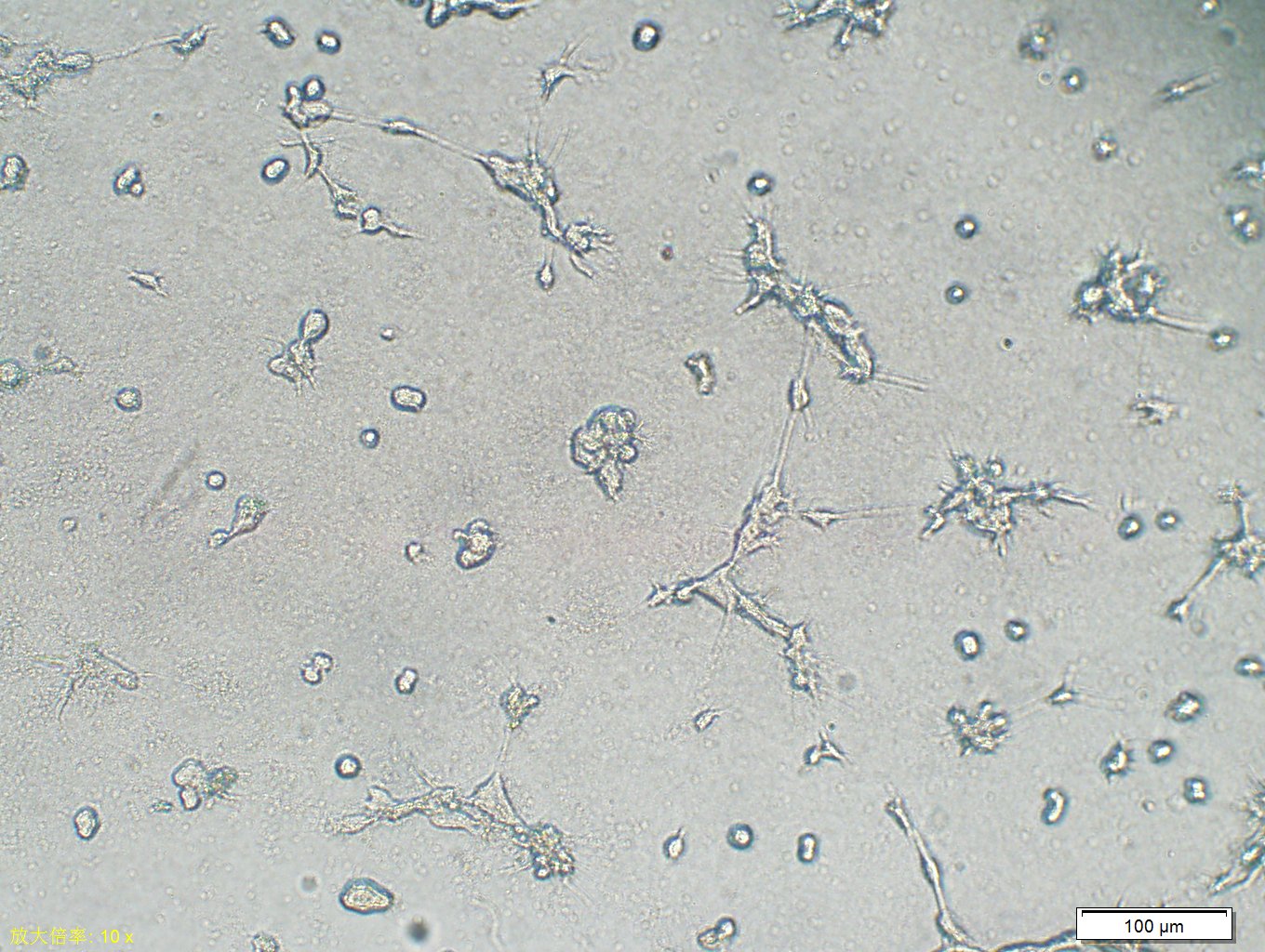


M0-CM+1E8 group


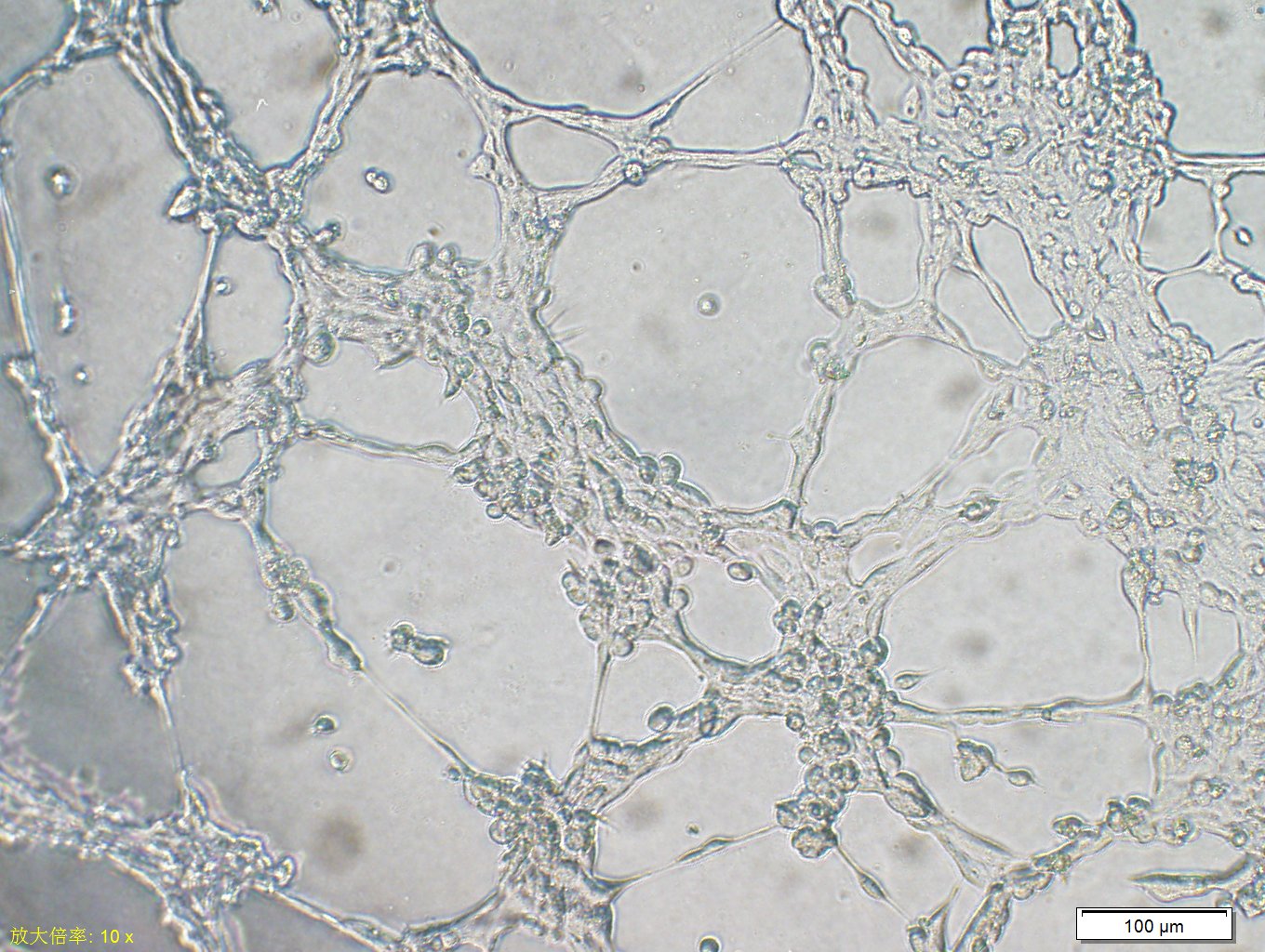


IL-4-M2-CM+2B4 group


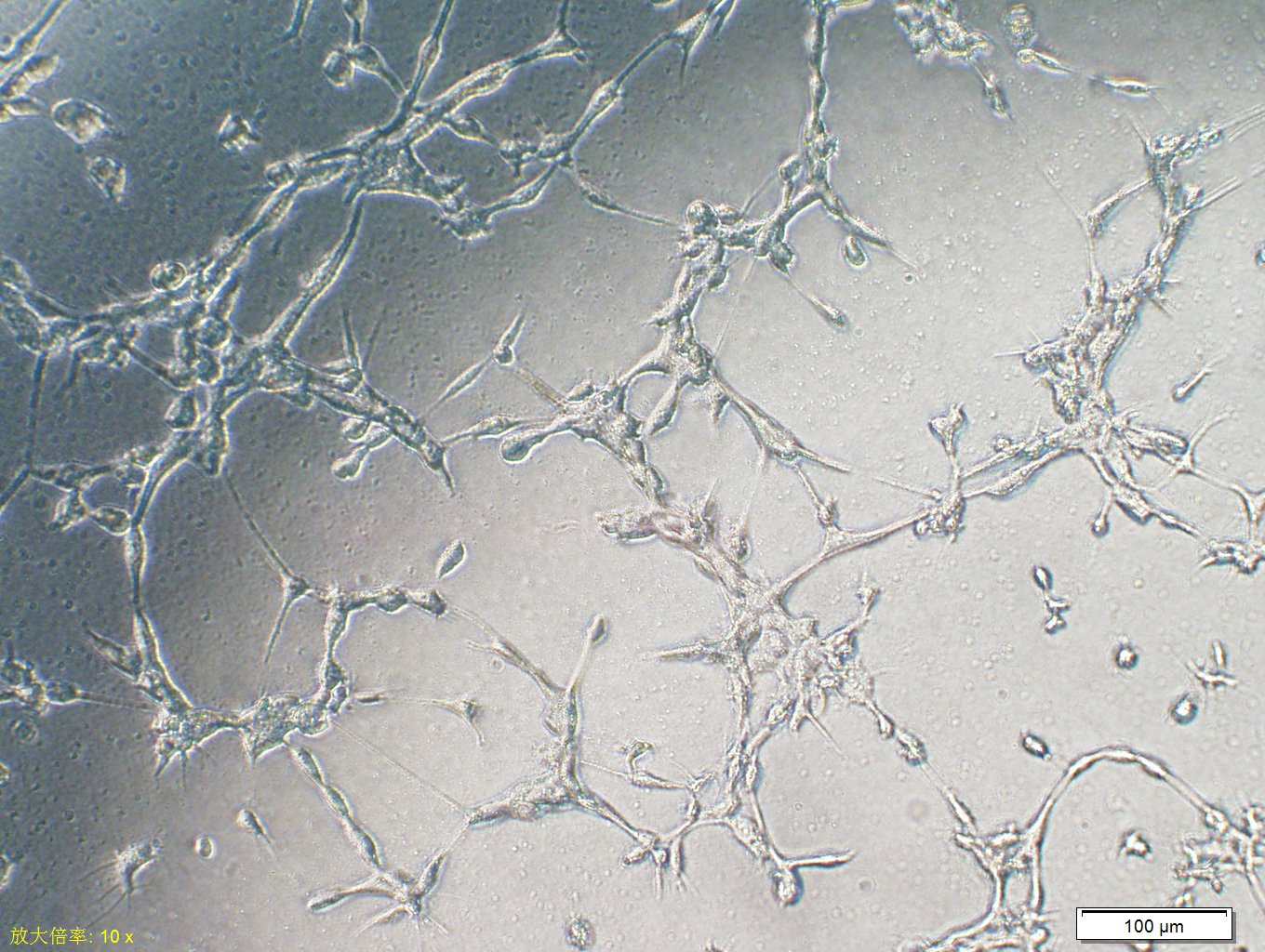


IL-4-M2-CM+1E8 group


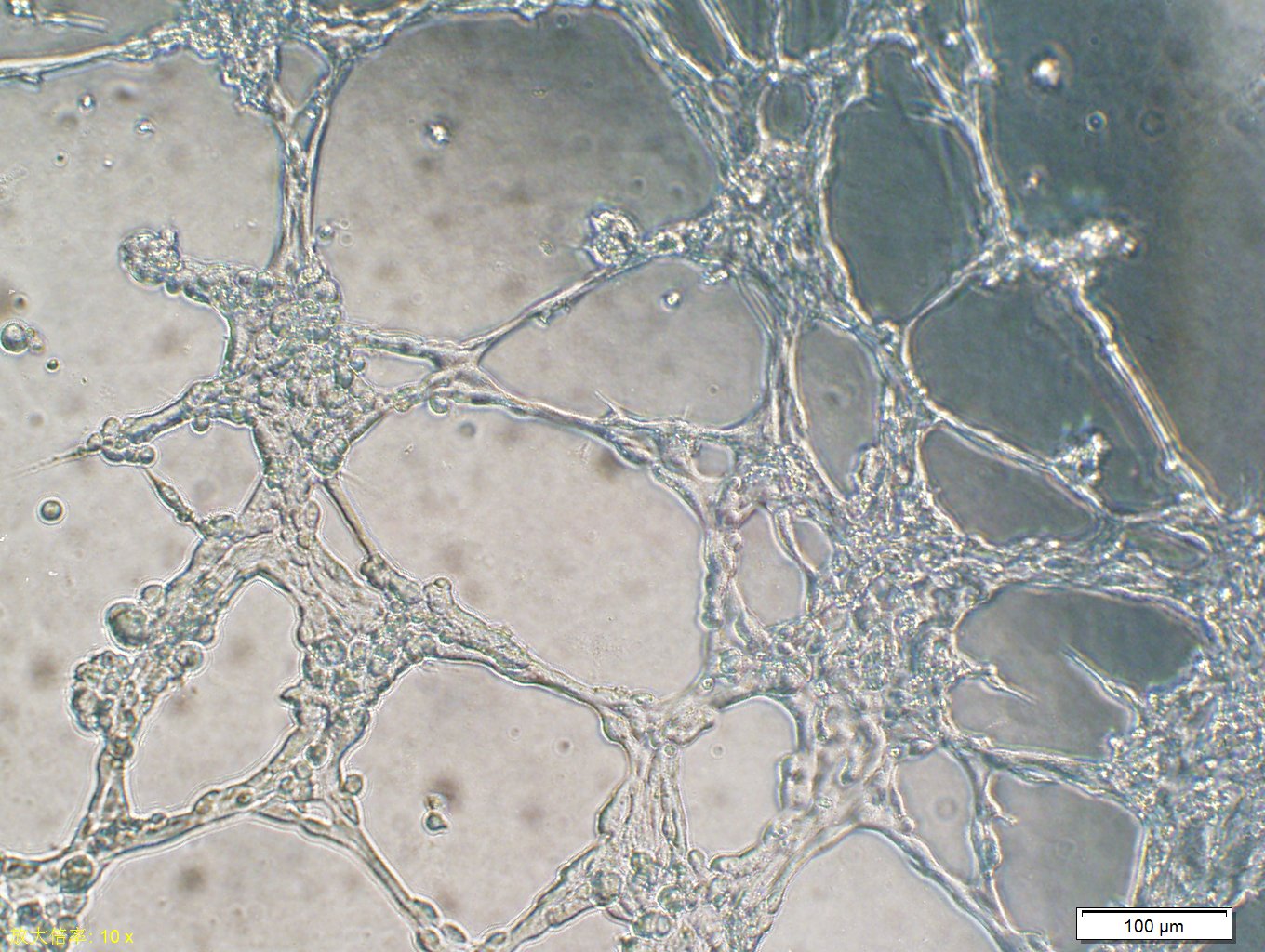


2B4-exo-Mø-CM+2B4 group


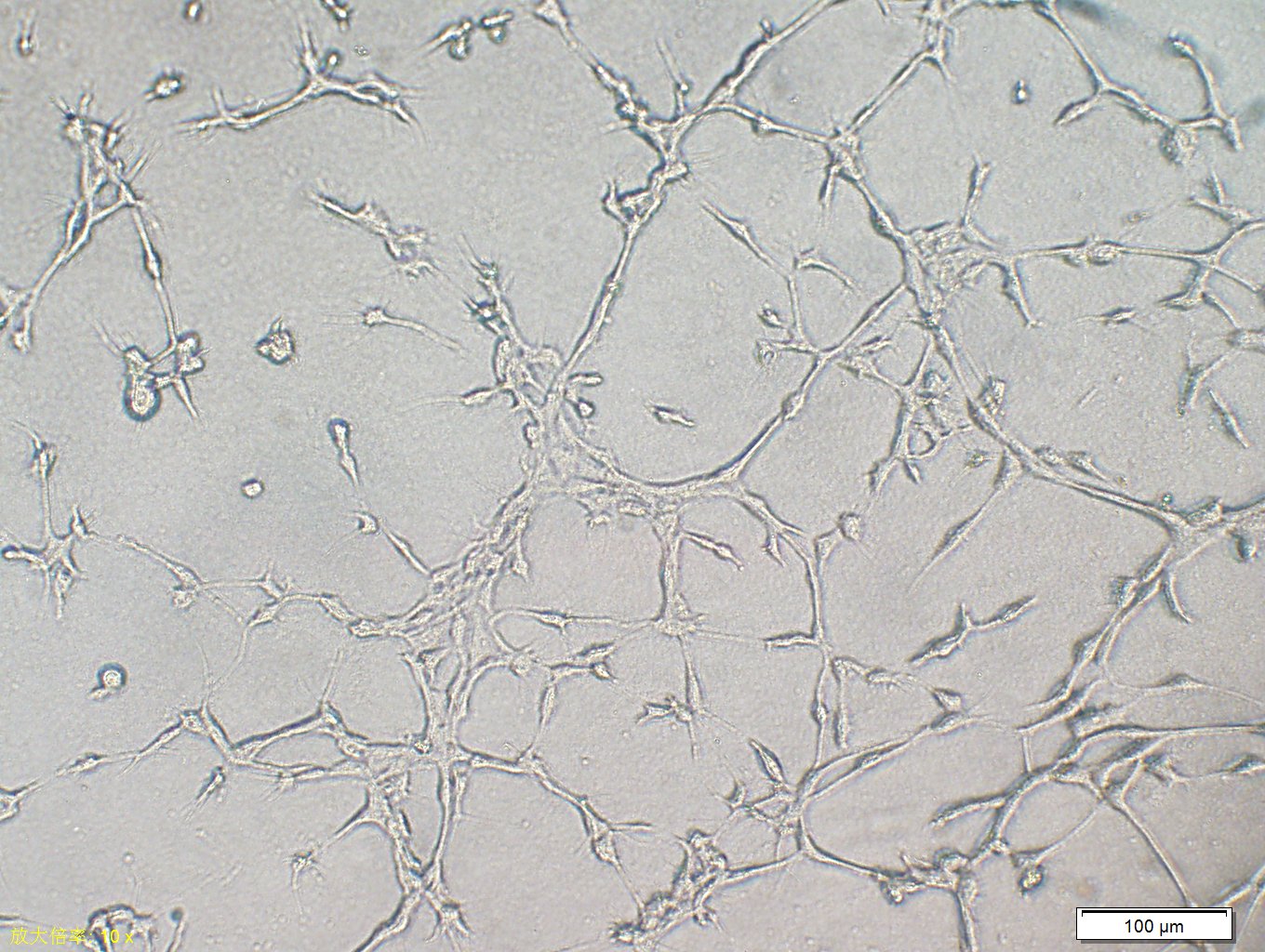


2B4-exo-Mø-CM+1E8 group


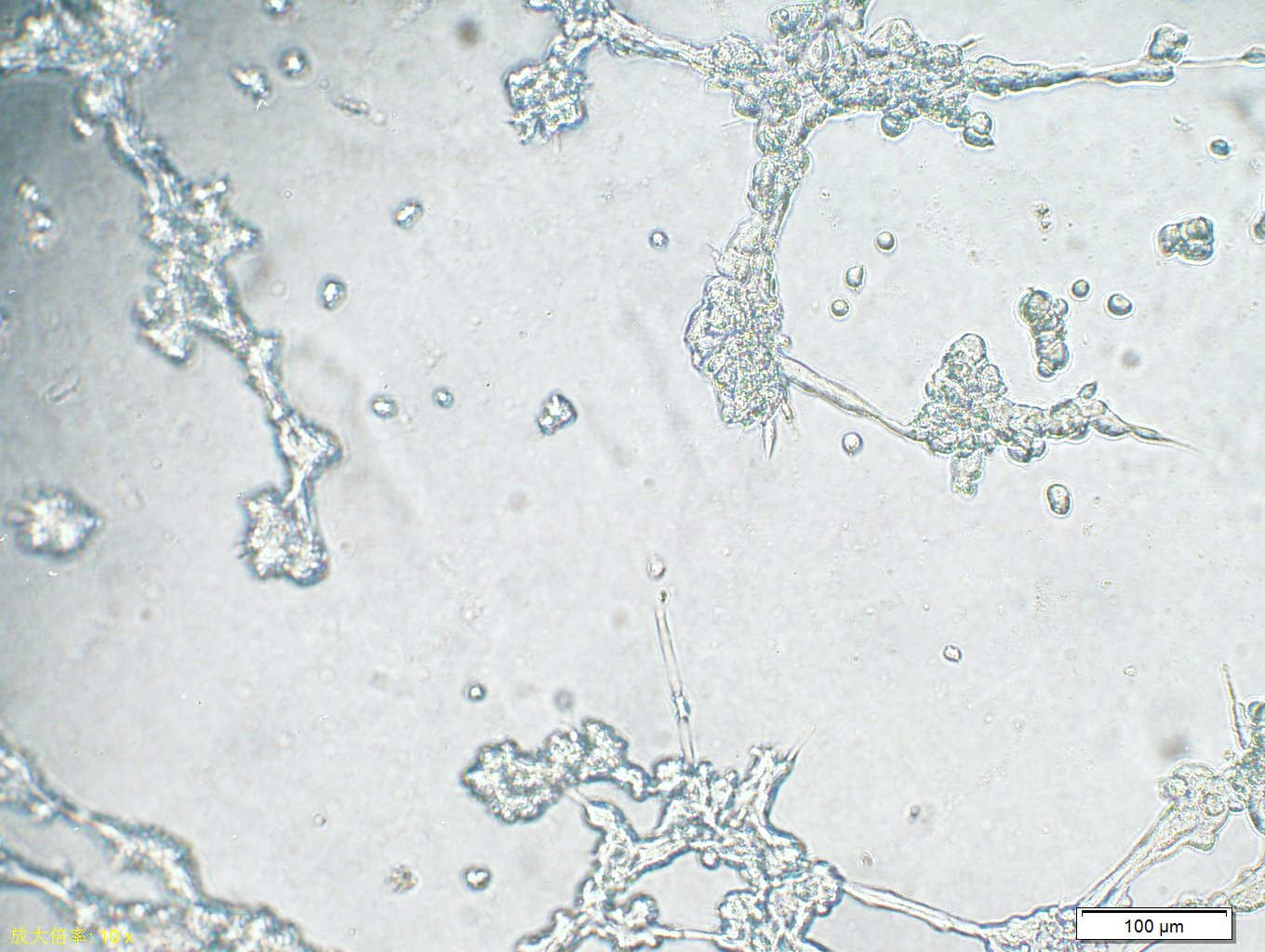


CM-2B4-GW4869-Mø-CM+2B4 group


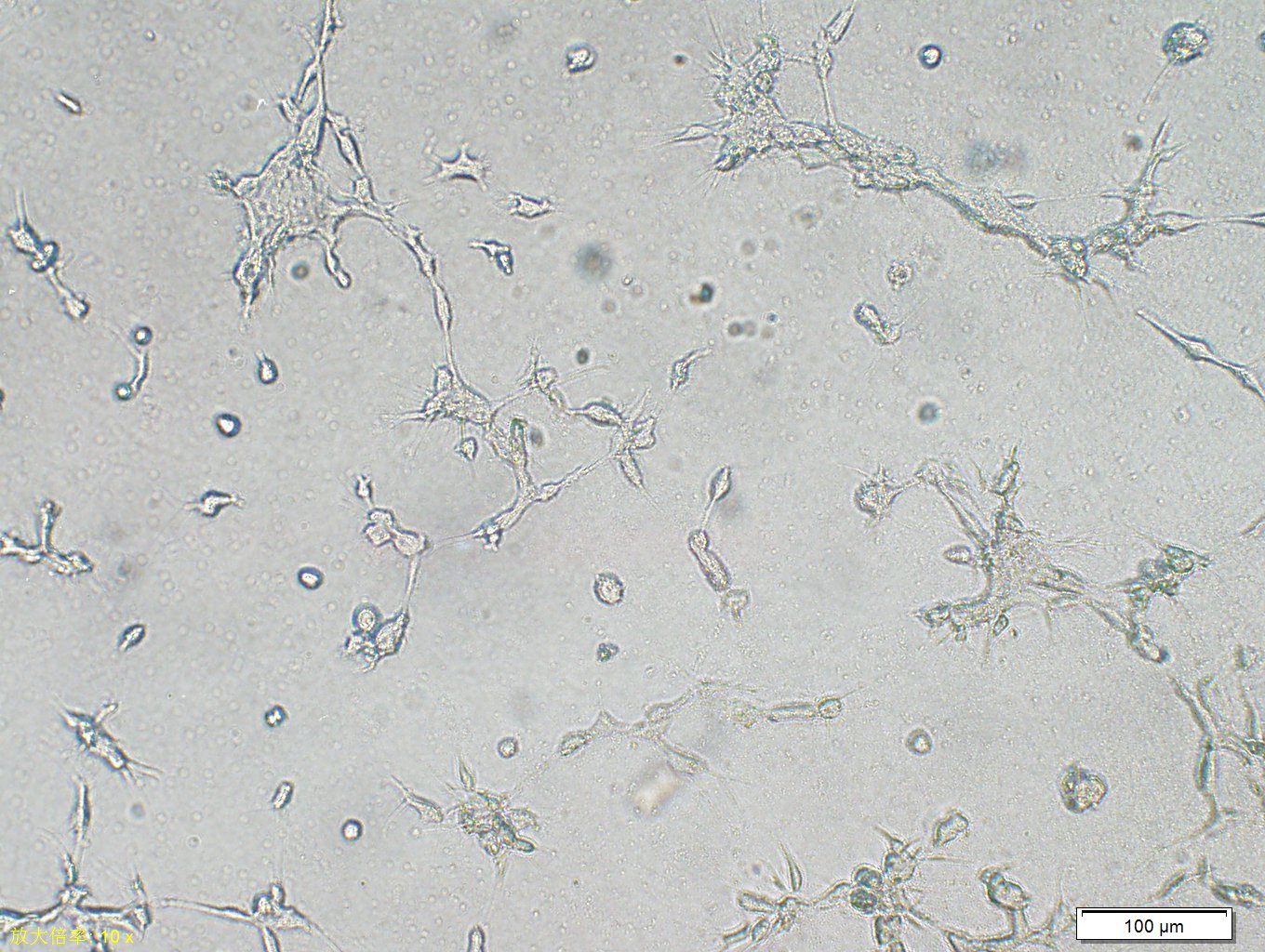


CM-2B4-GW4869-Mø-CM+1E8 group


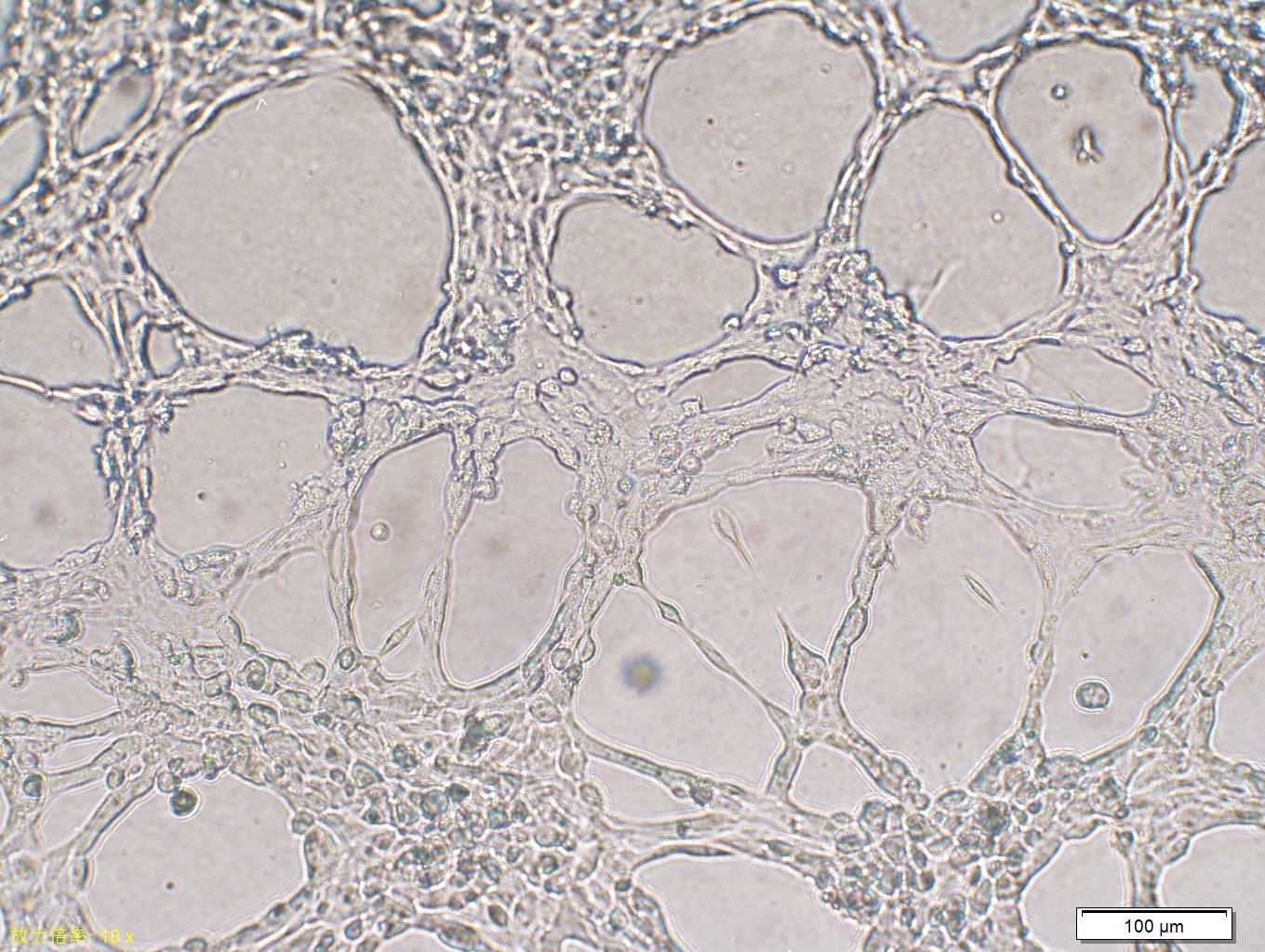


1E8-exo-Mø-CM+2B4 group


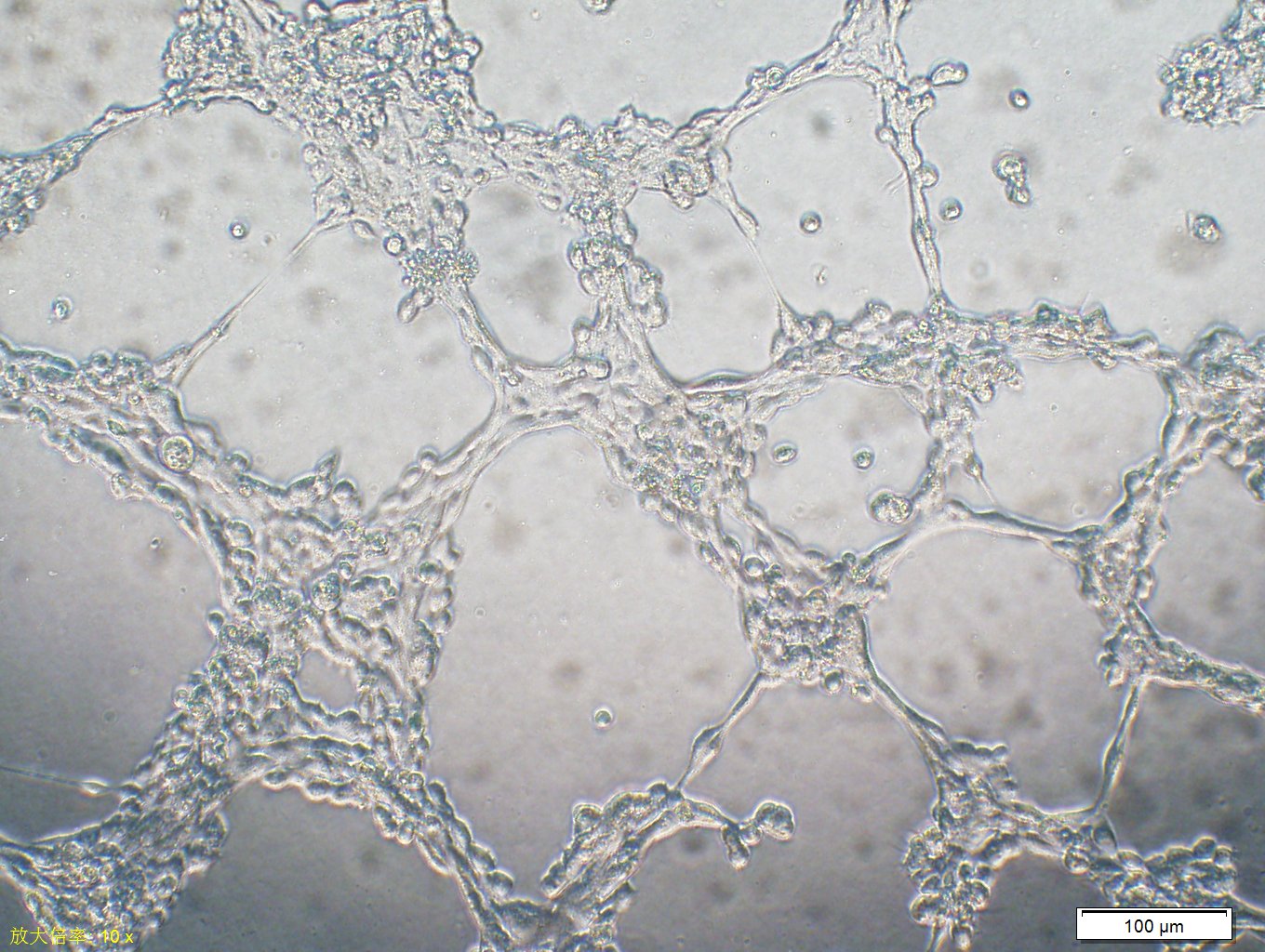


1E8-exo-Mø-CM+1E8 group


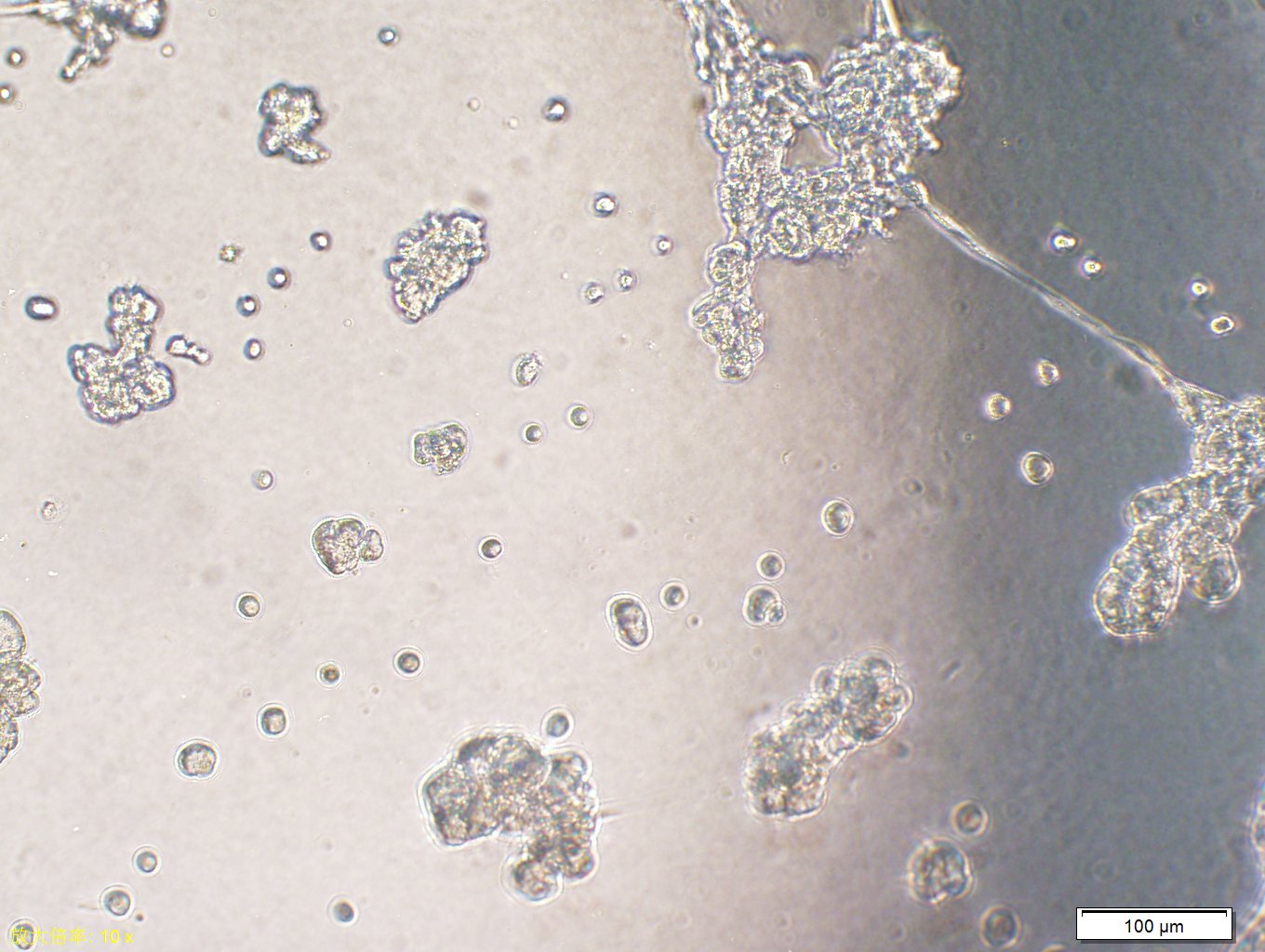


CM-1E8-GW4869-Mø-CM+2B4 group


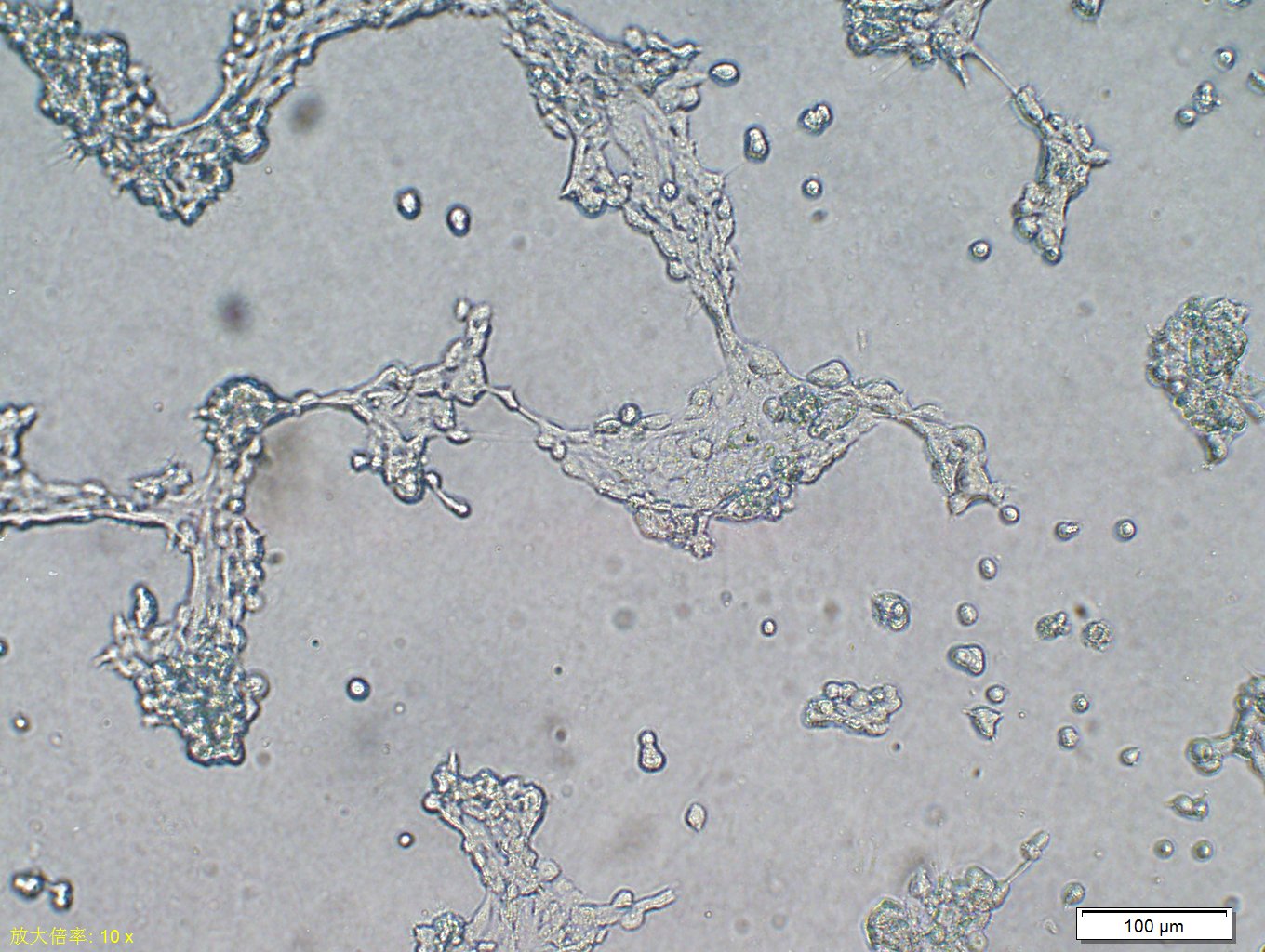


CM-1E8-GW4869-Mø-CM+1E8 group

**Figure S2.** **Activitiy of Akt and STAT3 signaling pathways in macrophages by Western blotting.** After blocking the release of PCa exosomes with GW4869, CM from PCa cells were collected and added to macrophages for 48 h. The Activitiy of Akt and STAT3 signaling pathways in these macrophages was determined by Western blotting.

**Lane 1 2 3 4 5 6 7**

**THP-1 2B4- 2B4-exo 1E8- 1E8-exo IL-4-M2 M1**

**GW4869 GW4869**

**β-actin
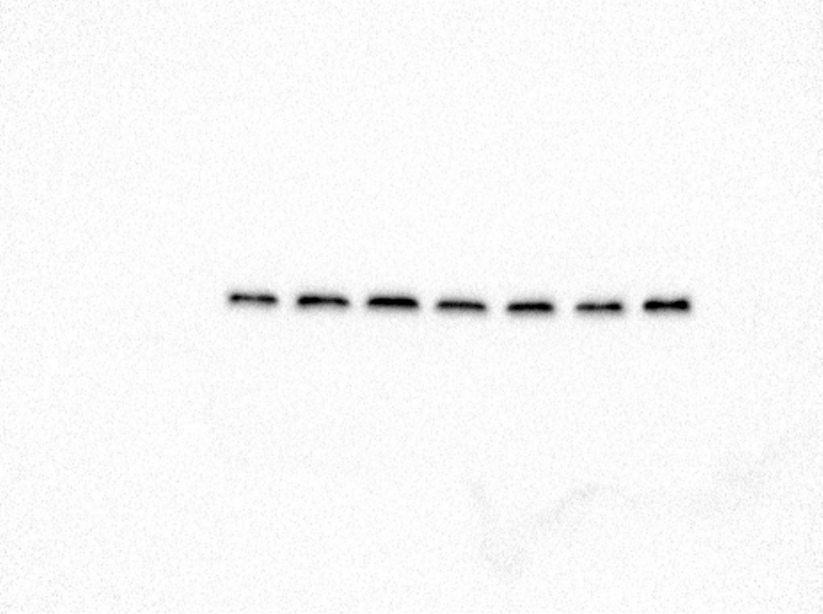
 42KDa**

**Exposure_3.0sec**

**
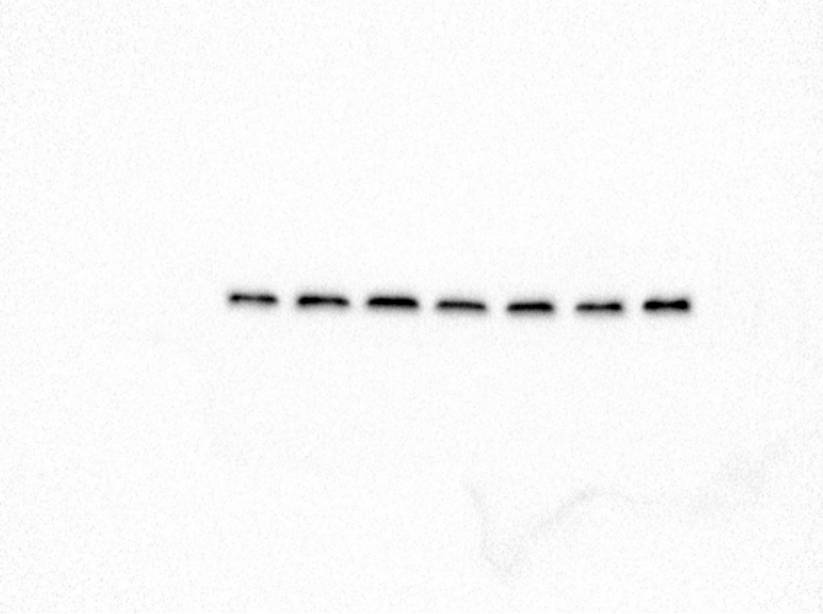
**

**Exposure_4.9sec**

**
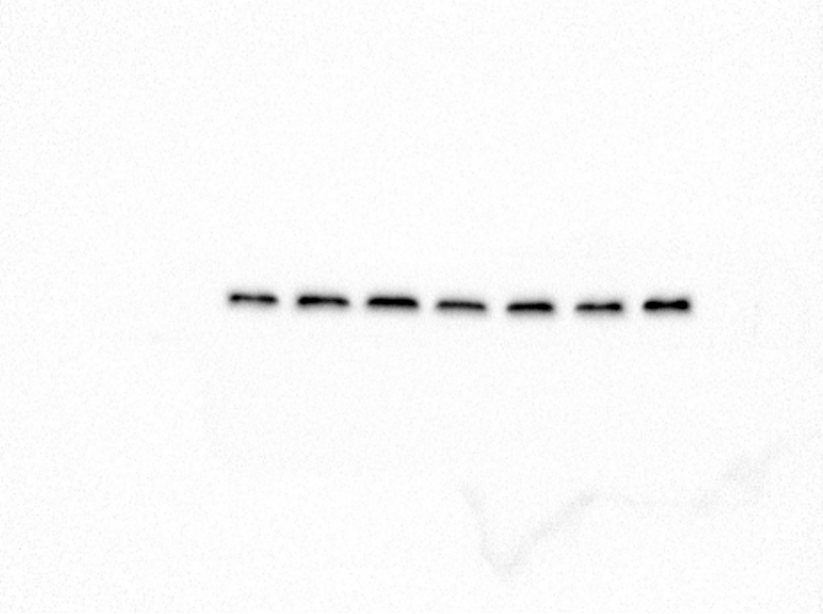
**

**Exposure_8.9sec**

**mTOR
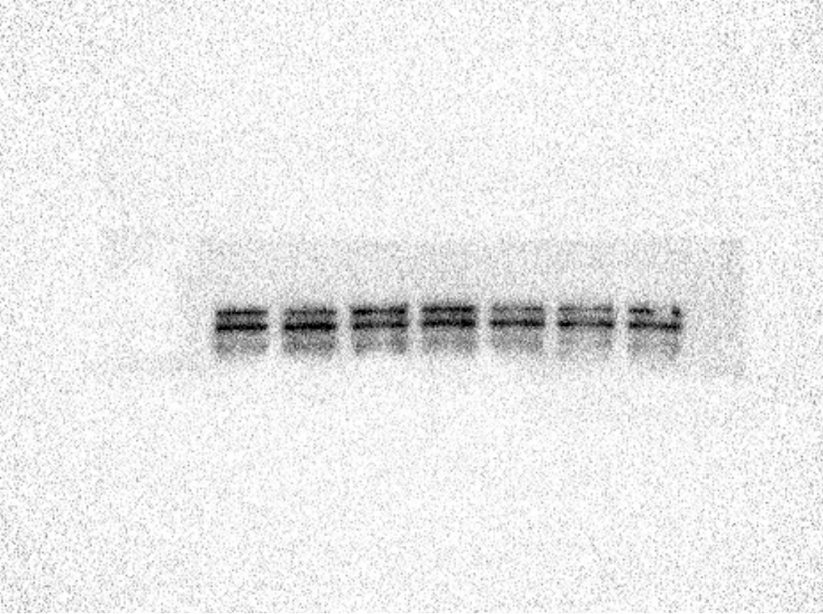
 250KDa**

**Exposure_1.0sec**

**
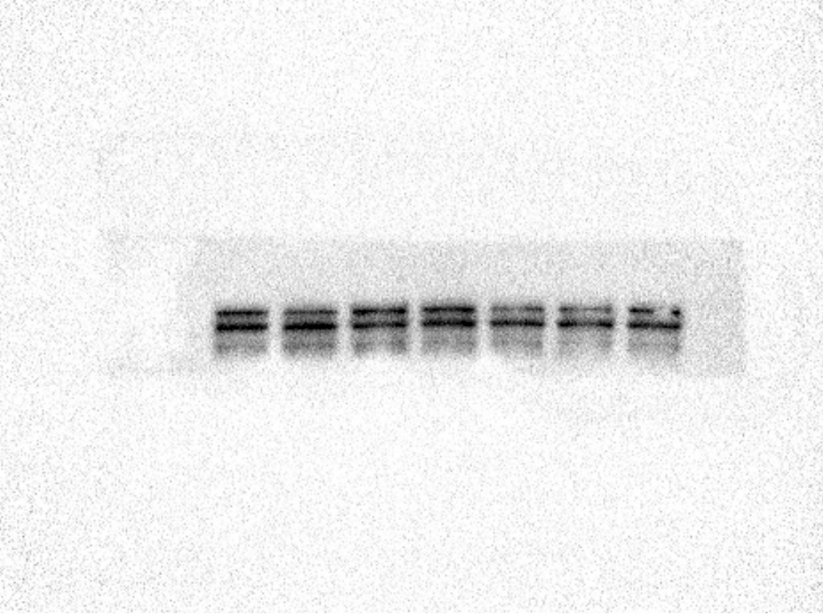
**

**Exposure_3.6sec**

**
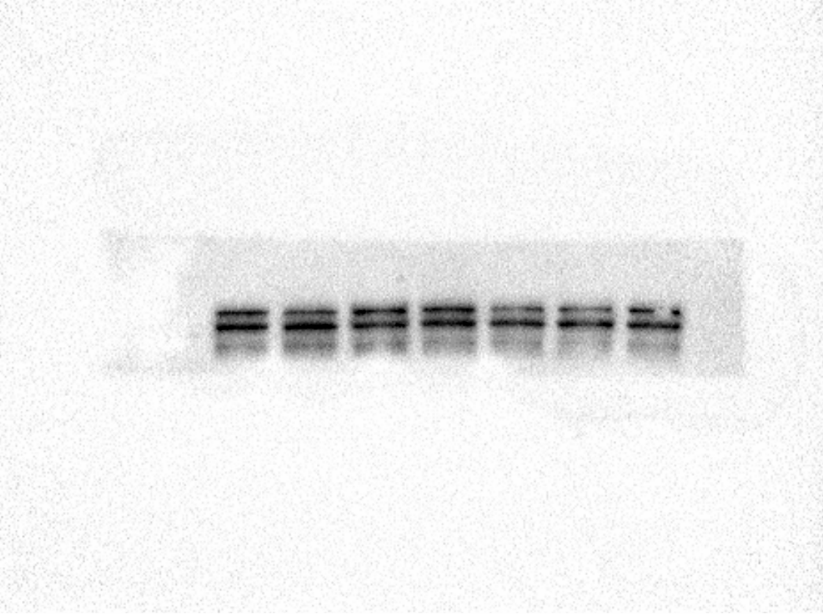
**

**Exposure_8.2sec**

**P-mTOR
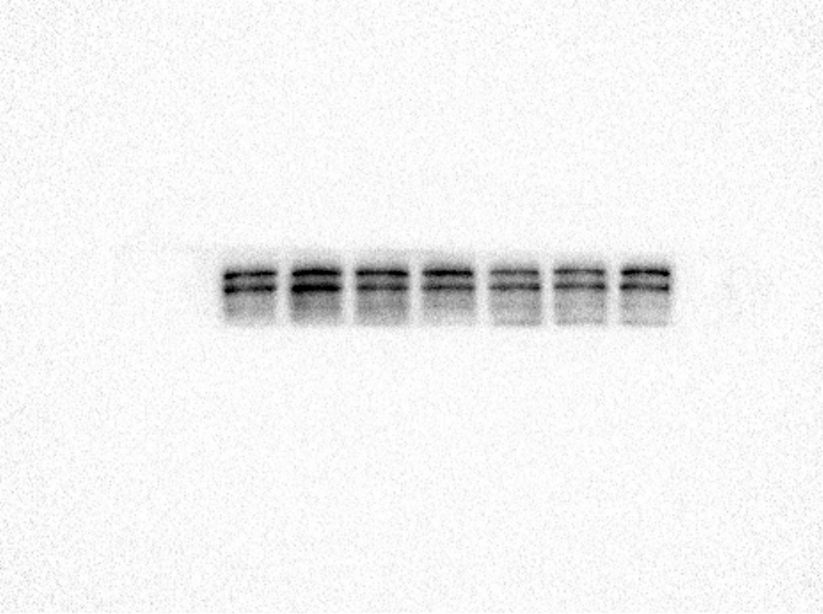
 250KDa**

**Exposure_1.0sec**

**
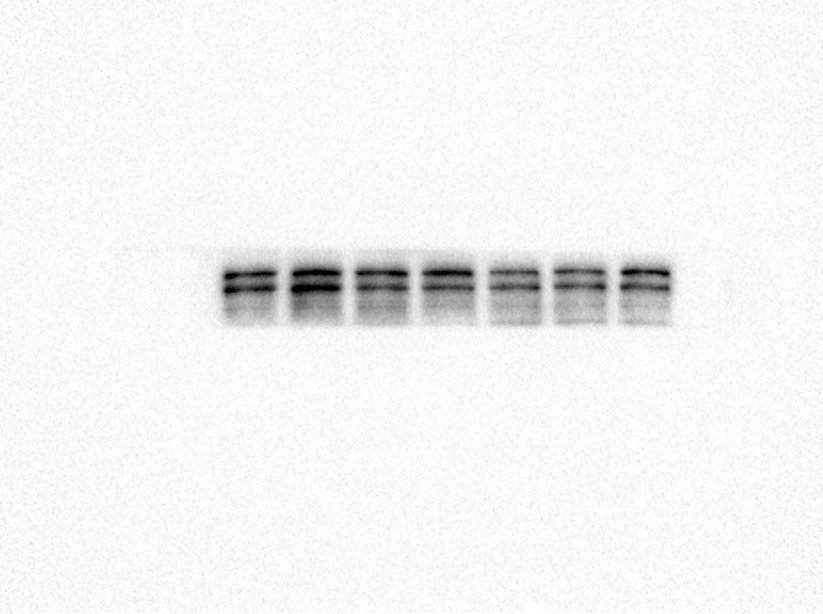
**

**Exposure_3.9sec**

**
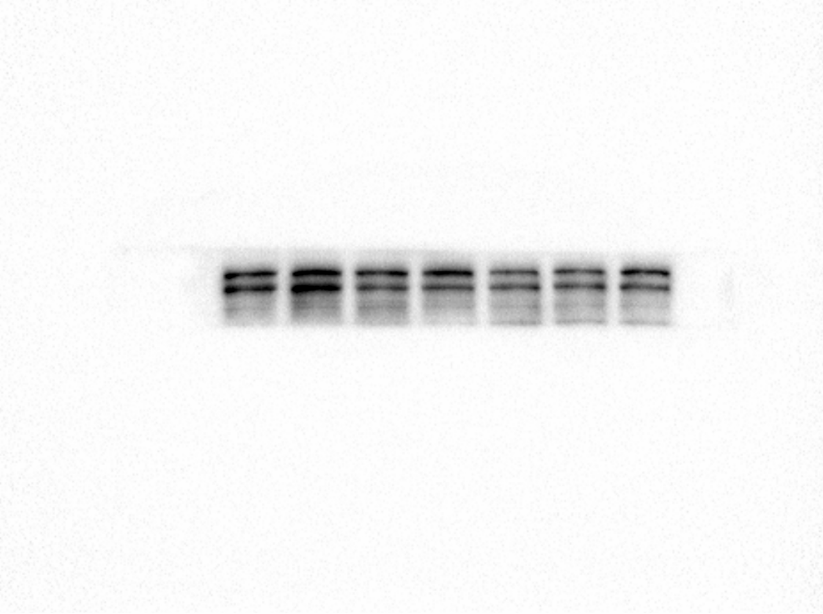
**

**Exposure_8.8sec**

**AKT
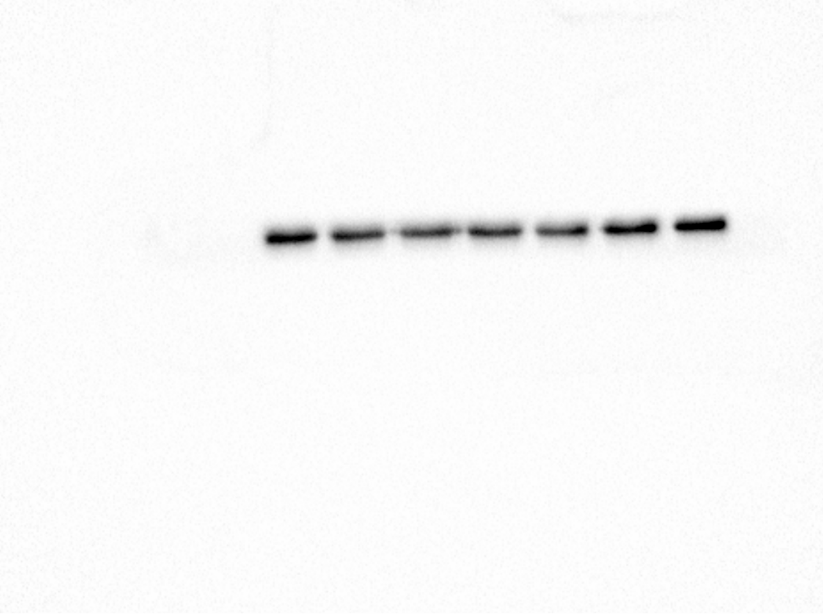
 60KDa**

**Exposure_1.0sec**

**
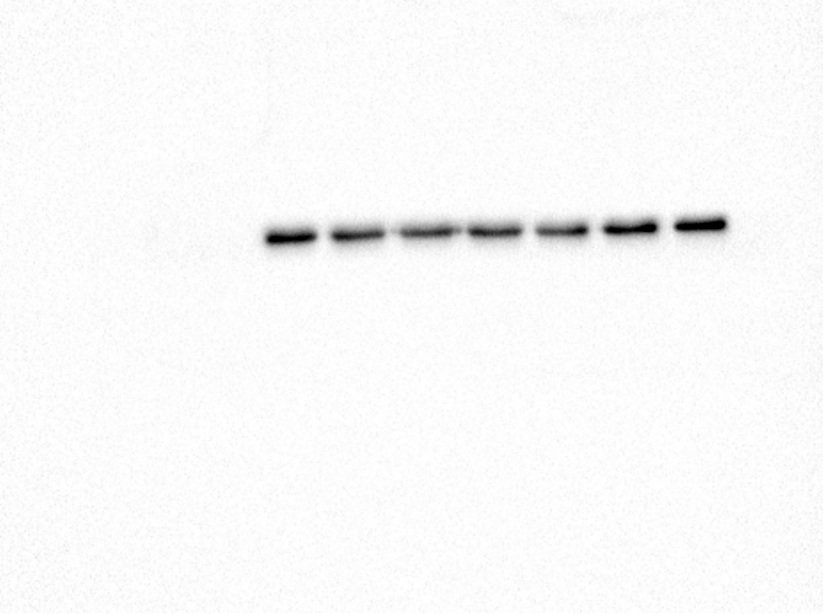
**

**Exposure_1.9sec**

**
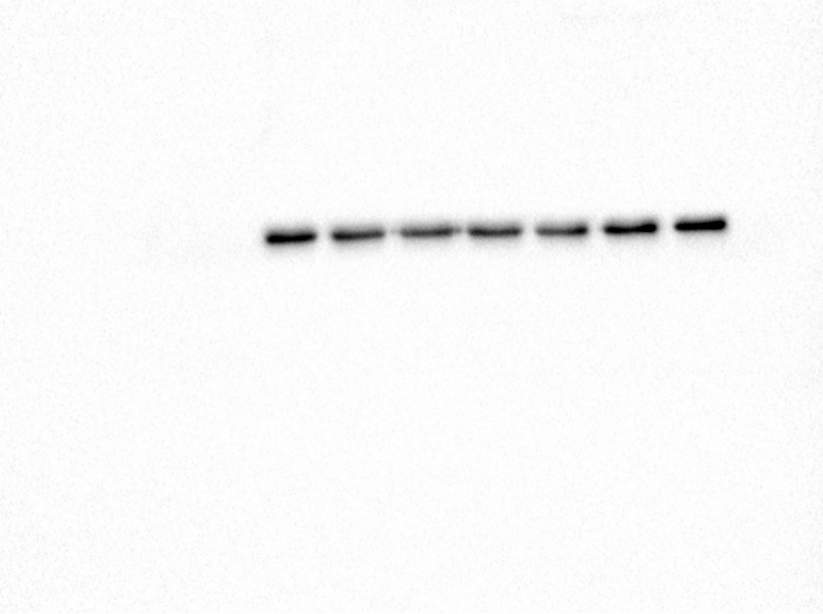
**

**Exposure_2.7sec**

**P-AKT
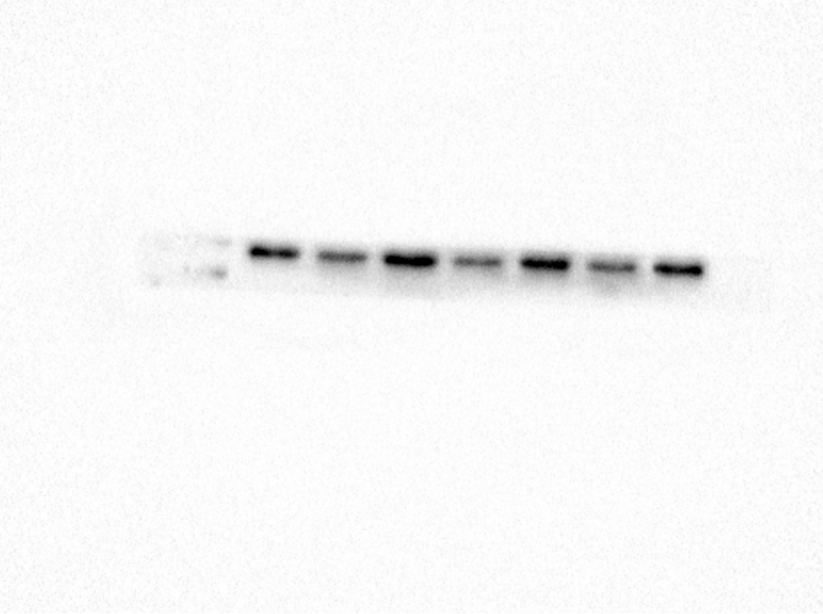
 60KDa**

**Exposure_1.0sec**

**
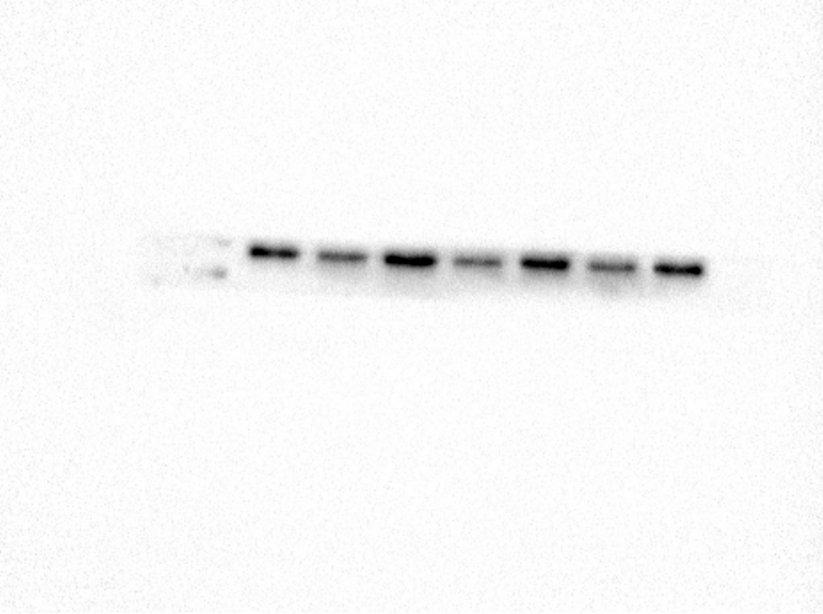
**

**Exposure_2.9sec**

**
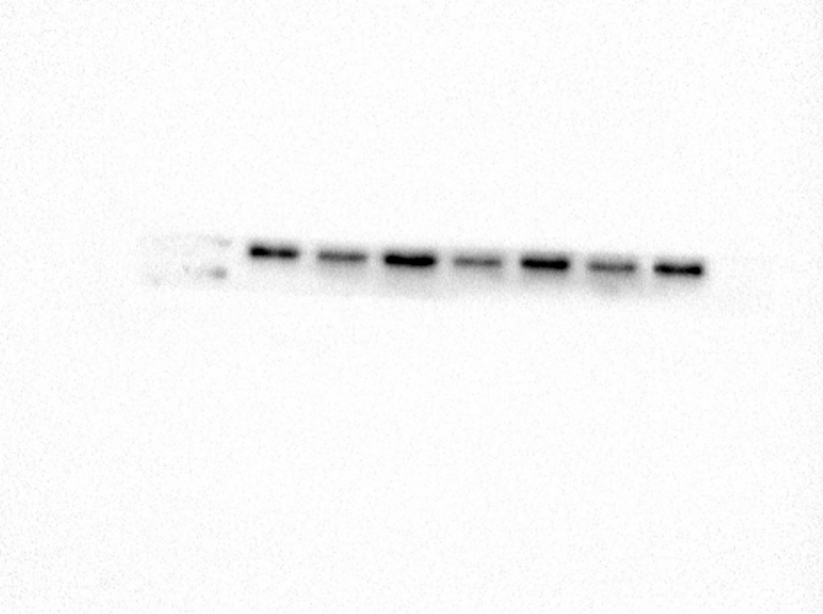
**

**Exposure_3.9sec**

**STAT3
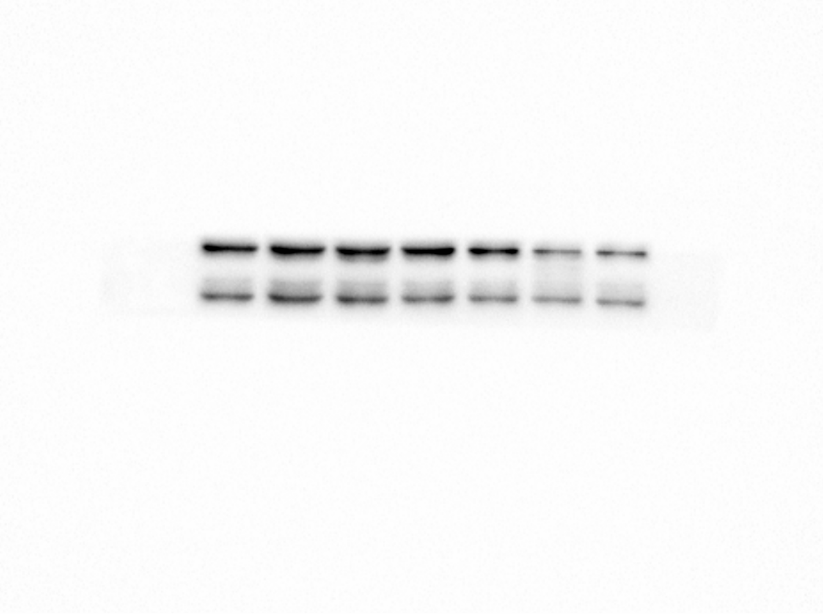
 79-92KDa**

**Exposure_1.0sec**

**
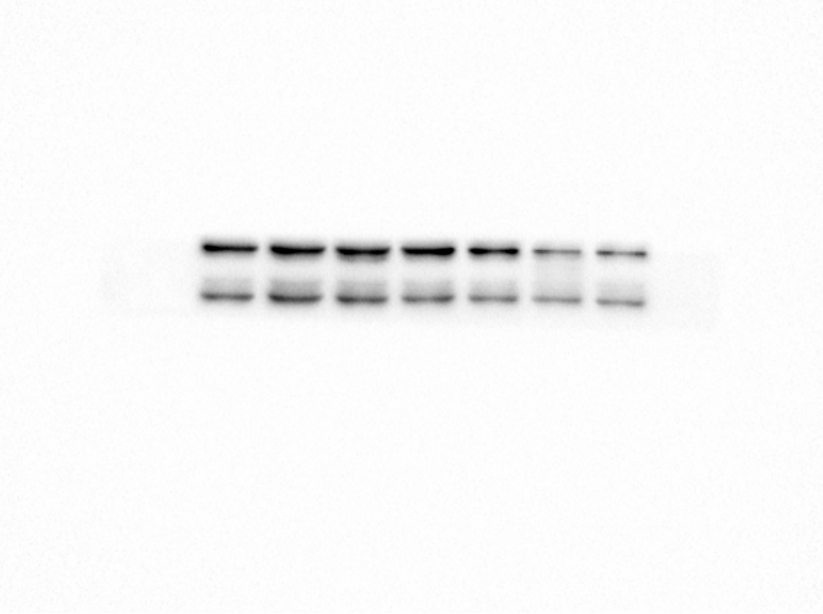
**

**Exposure_1.5sec**

**
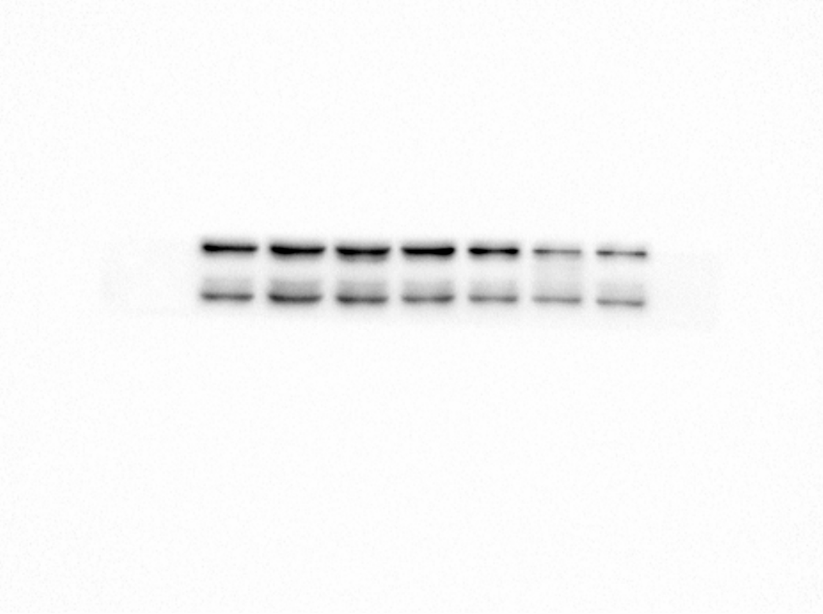
**

**Exposure_2.1sec**

**P-STAT3
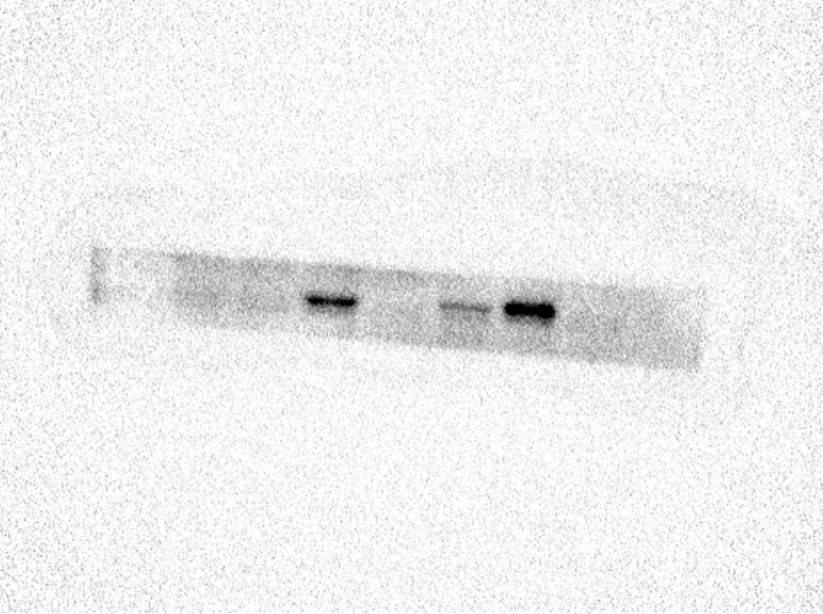
 79-92KDa**

**Exposure_1.0sec**

**
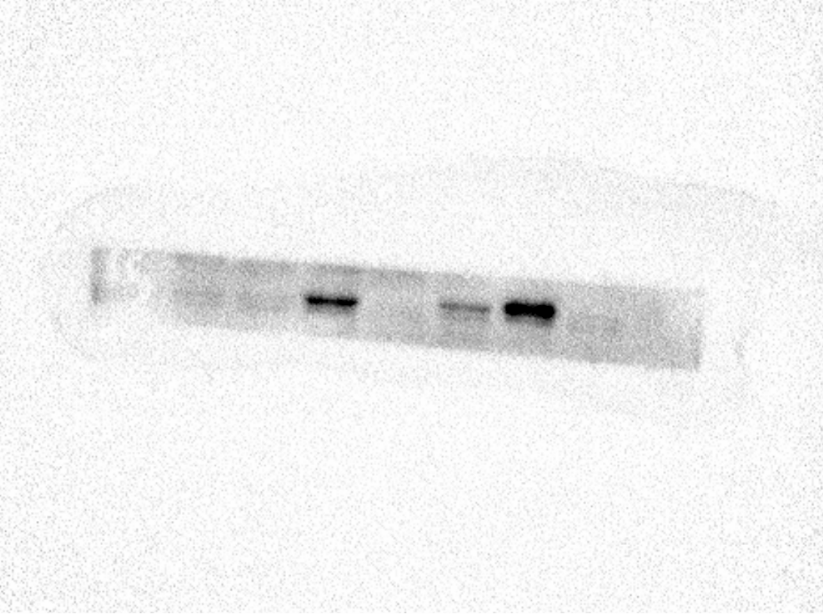
**

**Exposure_4.3sec**

**
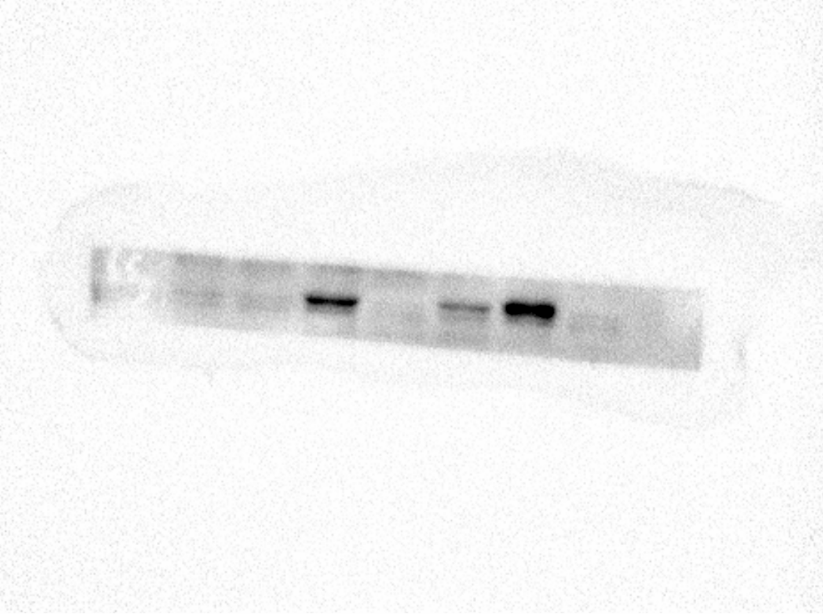
**

**Exposure_8.2sec**

**Figure 6.** **GW4869 inhibited the differentiation of tumor-associated macrophages into M2 phenotype *in vivo.*** A. The effect of GW4869 on the growth of prostate cancer in tumor xenograft mice (n = 5). After the subcutaneous injection of 5×10^6^ PC-3-M-1E8 cells or PC-3-M-1E8 cells treated with GW4869 cells in nude mice, tumor volume was measured every five days, where tumor volume (mm^3^) = shorter diameter^2^ × longer diameter/2.


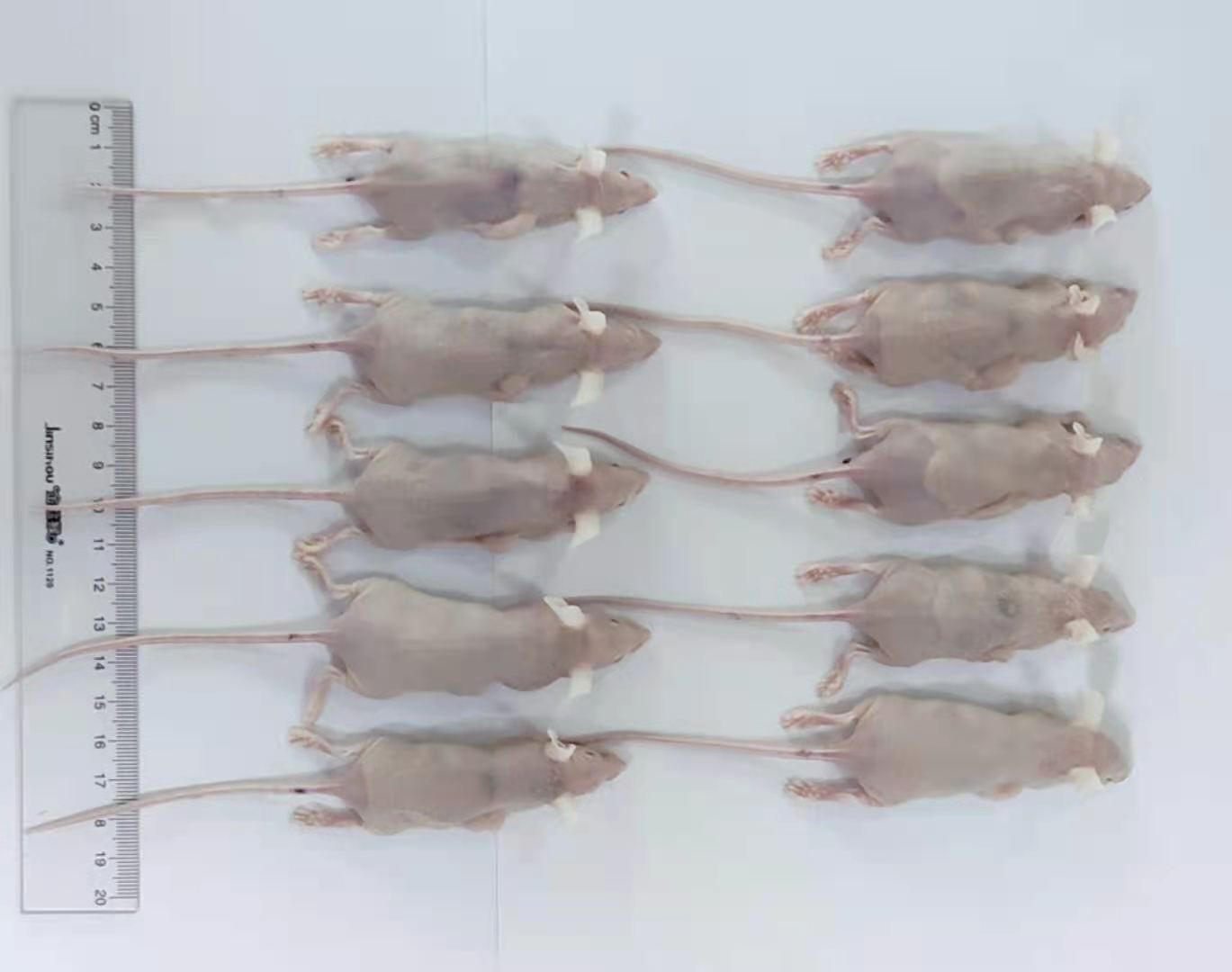


Nude mice


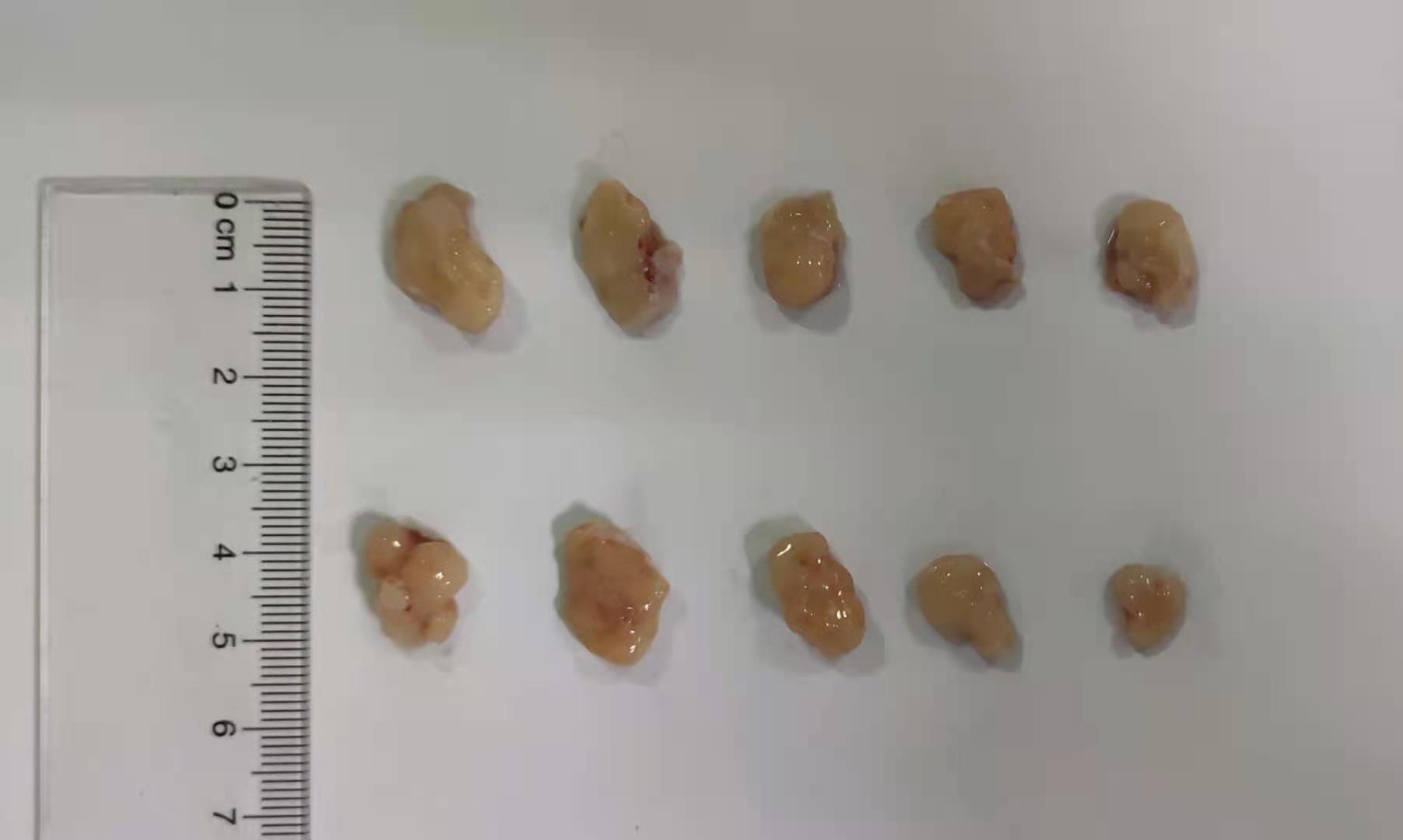


Tumor tissues

B. GW4869 reduced number of CD206+ macrophages in PCa tissues. After tumor formation, CD206 expression in tumor tissue was determined using immunohistochemical analysis. *P < 0.05,**P < 0.01.


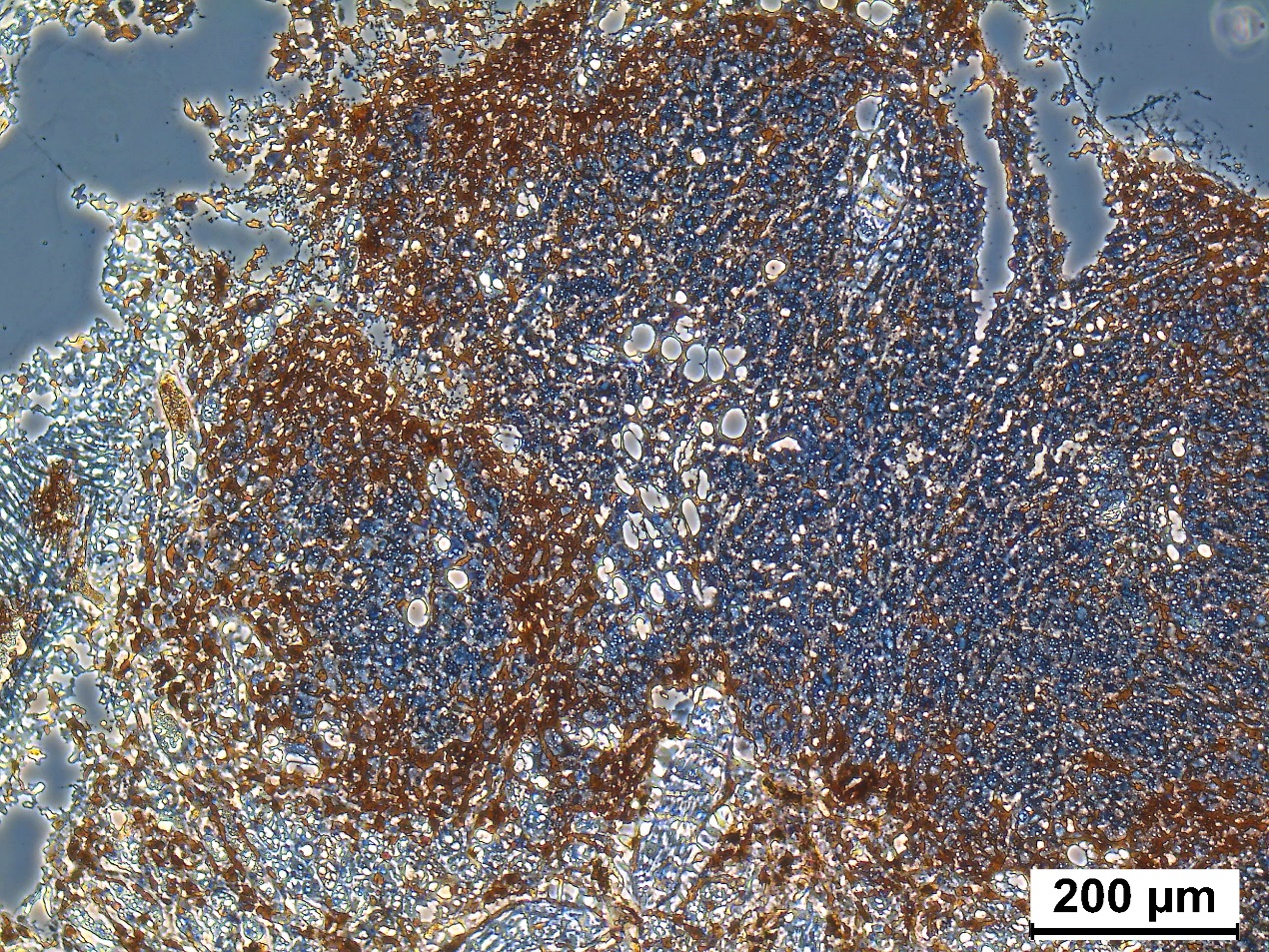


CD206 expression in control group (100X)


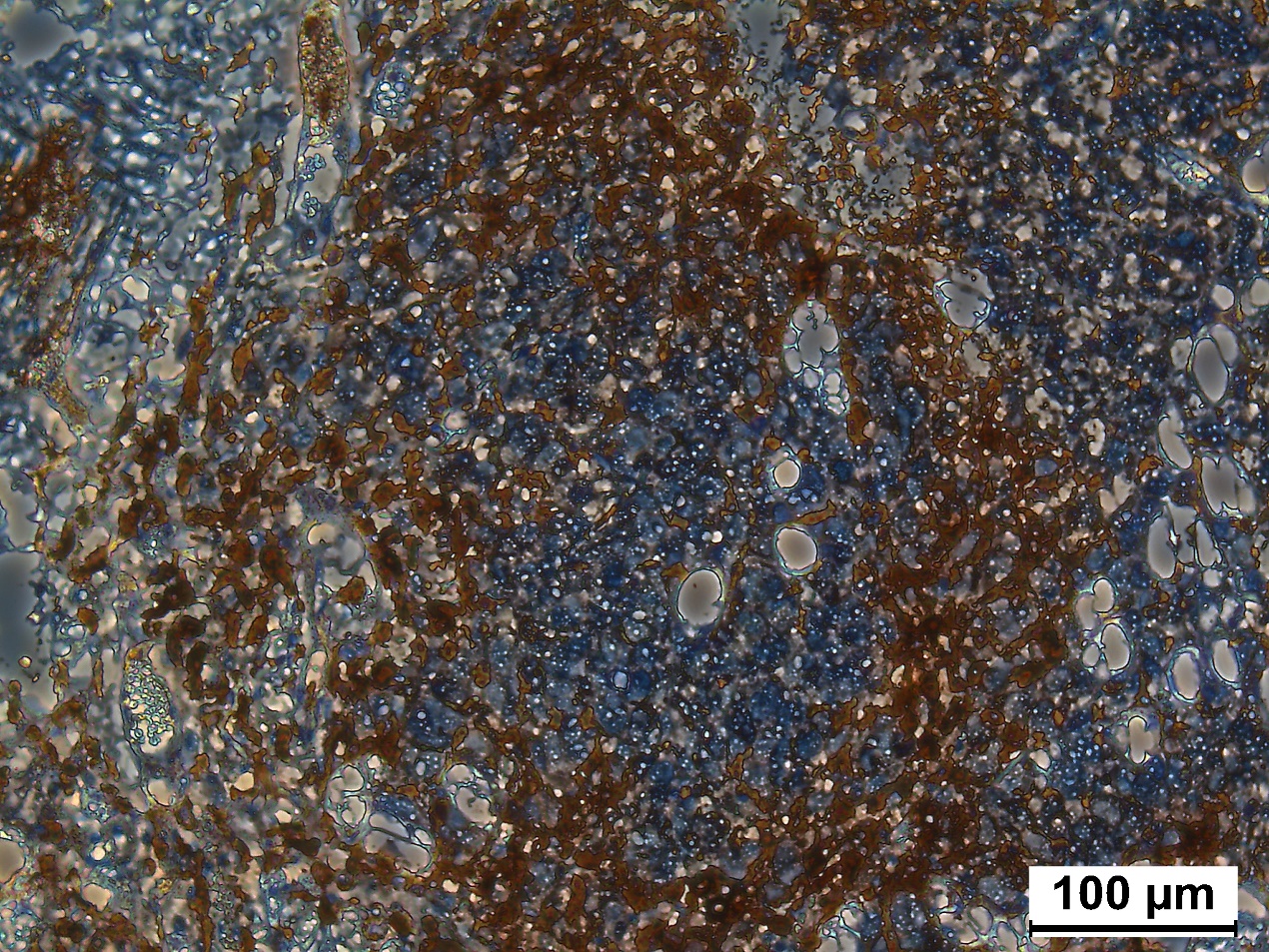


CD206 expression in control group (200X)


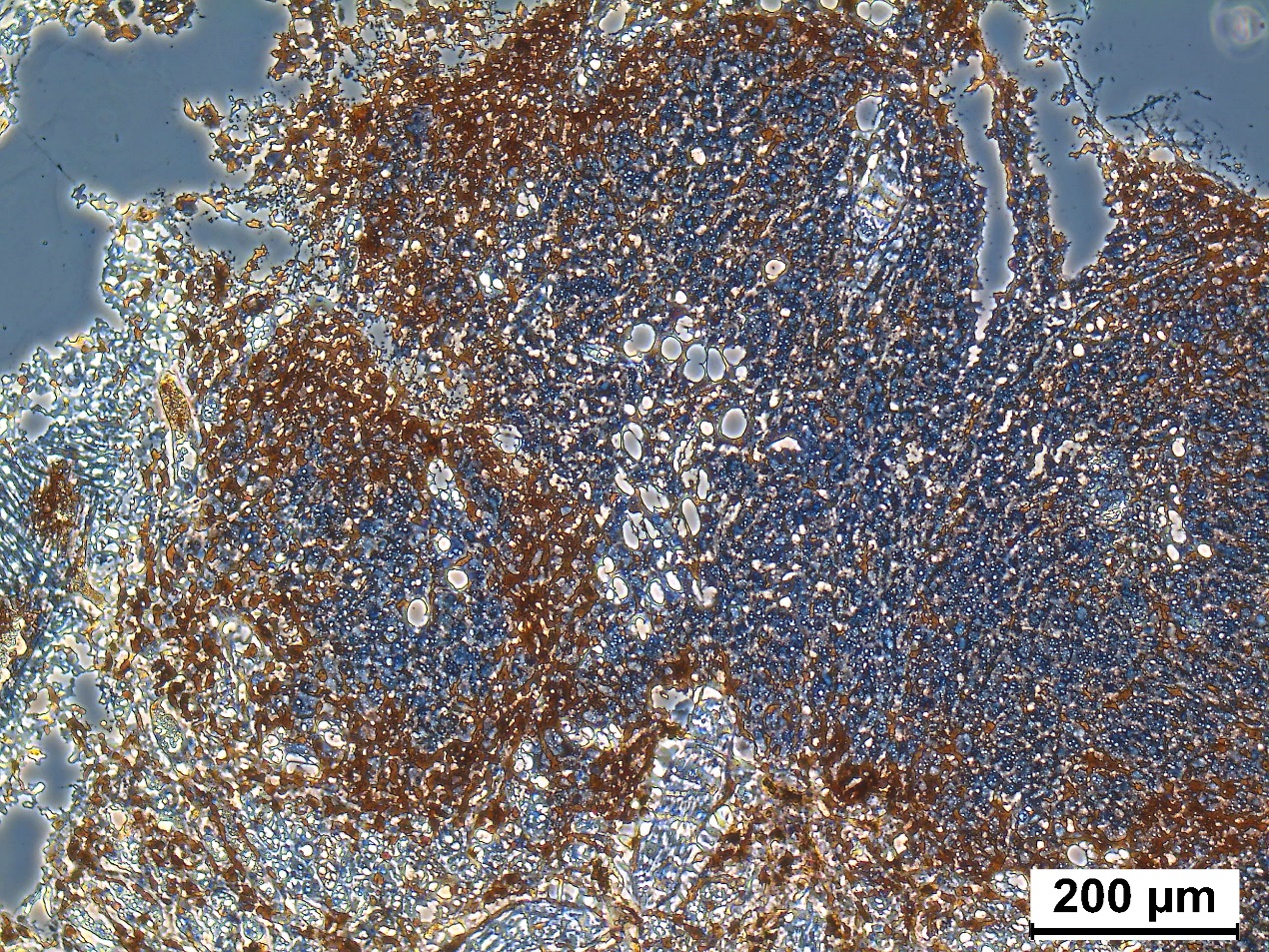


CD206 expression in GW4869 group (100X)


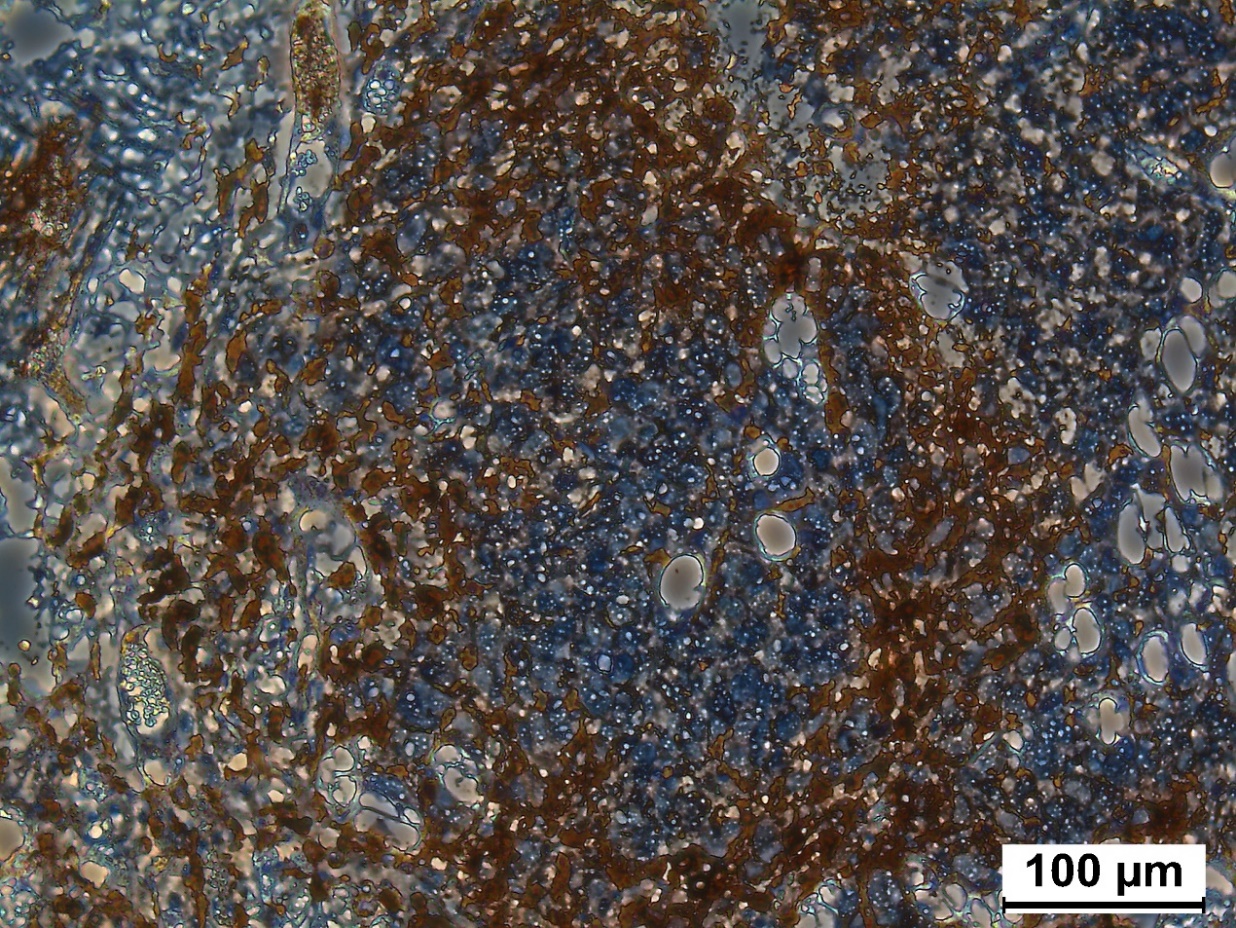


CD206 expression in GW4869 group (200X)
